# When details matter: Integrative revision of Holarctic *Coelophthinia* Edwards (Diptera, Mycetophilidae), including mapping of its mitogenome, leads to the description of four new pseudocryptic species

**DOI:** 10.3897/BDJ.11.e98741

**Published:** 2023-02-14

**Authors:** Jostein Kjærandsen, Peter H. Kerr, Jon Peder Lindemann, Olavi Kurina

**Affiliations:** 1 UiT – The Arctic University of Norway, Tromsø, Norway UiT – The Arctic University of Norway Tromsø Norway; 2 California State Collection of Arthropods, Sacramento, United States of America California State Collection of Arthropods Sacramento United States of America; 3 Institute of Agricultural and Environmental Sciences, Tartu, Estonia Institute of Agricultural and Environmental Sciences Tartu Estonia

**Keywords:** *Coelophthinia*, morphology, DNA barcoding, integrative taxonomy, new species, new mitogenome

## Abstract

**Background:**

The small genus *Coelophthinia* Edwards, 1941 of the subfamily Gnoristinae (Diptera, Mycetophilidae) is so far known to harbour four species from the Palaearctic, Nearctic and Neotropical Regions. Extensive DNA barcoding of fungus gnats of the family Mycetophilidae through the International Barcode of Life project (iBOL) have initiated integrative studies resulting in taxonomic upgrades and a better understanding of many species and their delimitation. The opportunity was also taken to describe the mitogenome of a member of *Coelophthinia* for the first time.

**New information:**

The integrative studies give evidence for splitting the European species *C.thoracica* Edwards, 1941 into three different species. Four new species are described from the USA, Japan and the Nordic Region in Europe, *Coelophthiniacirra* Kerr **sp. n.**, *Coelophthiniaitoae* Kurina **sp. n.**, *Coelophthinialata* Kjaerandsen **sp. n.** and *Coelophthinialoraasi* Kjaerandsen **sp. n.**, raising the number of Holarctic species from two to six. The mitogenome of *Coelophthinialoraasi* sp. n. is described and analysed.

## Introduction

The small genus *Coelophthinia* Edwards, 1941 of the subfamily Gnoristinae (Diptera, Mycetophilidae) is so far known to harbour four species, viz. *C.thoracica* (Winnertz, 1964) from the Palaearctic Region, *C.curta* (Johannsen, 1912) from the eastern Nearctic Region and *C.accita* (Plassmann & Vogel, 1990) and *C.flavithorax* (Freeman, 1951) from the temperate zone of the Neotropical Region (Oliveira and Amorim 2014). Søli (1997)further transferred a Brazilian species, *Coelosianeotropica* Lane, 1959, to *Coelophthinia*, but the original status of the species in *Coelosia* Winnertz, 1864 was reinstated by Oliveira and Amorim (2014).

**Figure 1.**
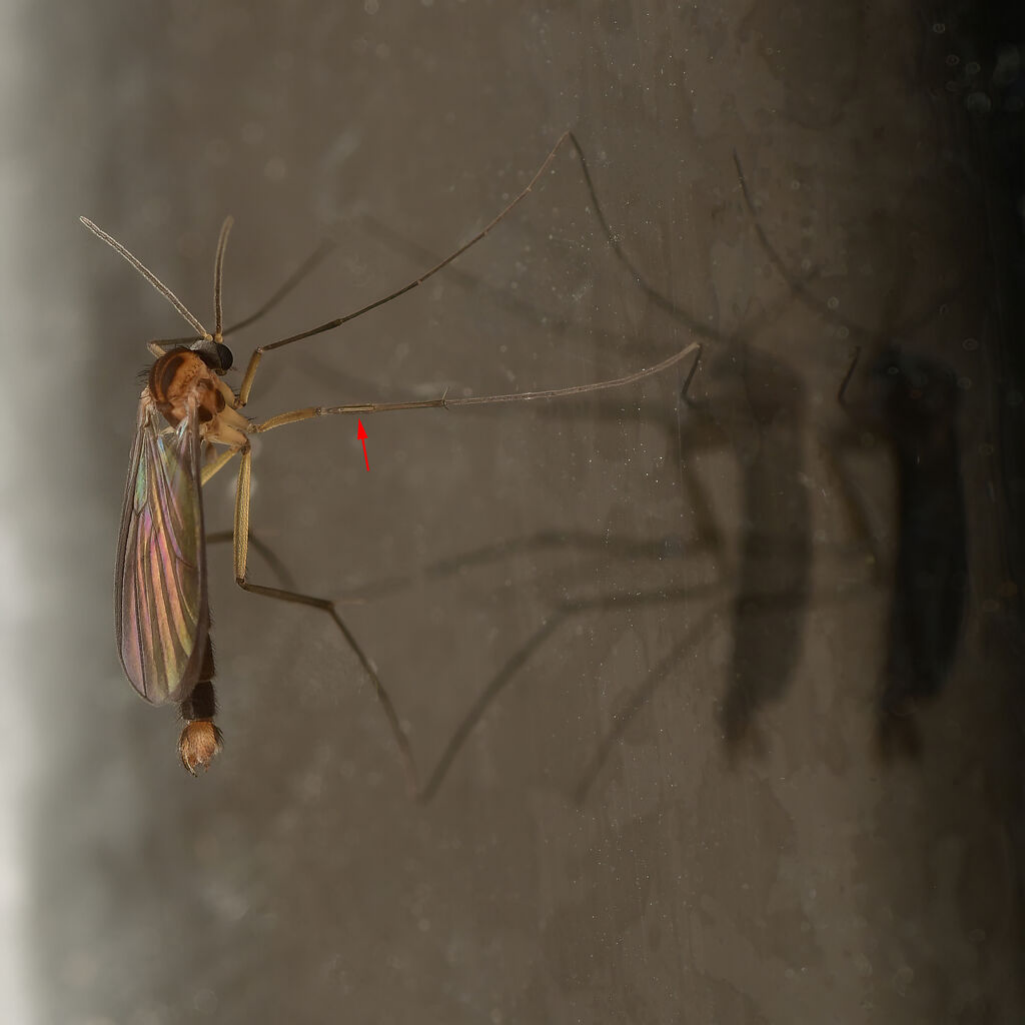
Live photo of the type species for the genus, a *Coelophthiniathoracica* (Winnertz, 1864) male resting on a window in a barn in western Norway. The photographed specimen was subsequently collected, DNA barcoded and assigned to Barcode Index Number (BIN) BOLD:ACJ0721 (Specimen ID TSZD-JKJ-111214). The mid-tibial organ is visible and marked with a red arrow. The 180 deg. torsion of the male terminalia is also visible.

Due to their slender appearance (Fig. 1), species of the genus *Coelophthinia* were placed together with *Phthinia* Winnertz, 1864, until Edwards (1925)moved the type species *C.thoracica* to *Coelosia* Winnertz, 1864. Edwards, however, later realised that this species was distinct enough to warrant separate generic status and, with a summary note, erected the genus *Coelophthinia* Edwards, 1941. Similar to what is found in several other species and genera of the family Mycetophilidae, *Coelophthinia* has developed a large, special sensory organ in their mid-tibia (Fig. 1), consisting of a long groove filled with sensory hairs. In *Coelophthinia*, both sexes have this structure developed, while in most of the other genera, a similar structure is only found in the males. Its possible function remains unclear, pending further histological examination (Kallweit 2013).

Extensive DNA barcoding of fungus gnats of the family Mycetophilidae through the International Barcode of Life project (iBOL, see Hebert et al. (2016) and Kjærandsen (2022)) and local initiatives like the Norwegian Barcode of Life project (NorBOL, see Kjærandsen and Søli (2020)) and the Finish Barcode of Life (FinBOL, see Roslin et al. (2021)) have contributed to taxonomic upgrades and a better understanding of many species and their delimitation. With support from the Barcode Index Number (BIN) system on BOLD, new evidence for splitting old species interpretations into two or multiple species emerges for many taxa. This kind of evidence must, however, be used with care (see, Ahrens et al. (2021)) and combined with morphological analyses in integrative studies. Using such an integrative approach here, we argue for splitting the European species *C.thoracica* into three different species, the North American species *C.curta* into two species and we describe one further, new species from Japan. This raises the number of Holarctic *Coelophthinia* from two to six species. We have also sequenced the mitogenome of one of the new species through genomic skimming and present its organisation and gene order as a representative for the genus.

## Materials and methods

### Specimen preparation and storage

The studied material has accumulated over the last 50 years, the majority during the last decade and is deposited in the insect collections of Tromsø University Museum, Norway (TMU), Estonian University of Life Sciences, Tartu, Estonia (IZBE) and California State Collection of Arthropods, Sacramento, California, USA (CSCA). Additionally, DNA barcoded material was borrowed from the Centre for Biodiversity Genomics, University of Guelph, Canada (BOLD). Being initially stored in 70-95% ethanol, the majority of the fresh specimens were dried through baths of hexamethyldisilazane (HMDS, Brown (1993)) and pinned during the study. A few specimens are mounted in Canada balsam on slides. Terminalia were detached from the abdomen and treated by standard methods (macerated either in warm lactic acid or in a solution of potassium hydroxide (KOH), cleaned in distilled water or neutralised in acetic acid) and transferred to glycerol. Images of specimens and their terminalia were captured with different microscopes. Z-stacked image series were processed into extended focus images by the Helicon Focus software enabling some manual editing of layers for increased visibility of specific characters. Extended focus images were further processed with Adobe Photoshop to adjust levels and contrast, reduce shadows, remove dust particles and clean up the background. Individual images were then processed by the Topaz Sharpen AI software to remove blur and suppress noise for enhanced sharpness. Finally, individual images were arranged into species plates, with identical angles of view for each species to ease comparison among the species. After detailed study and imaging, the terminalia were placed into micro-vials with glycerine and pinned together with the rest of the specimen.

### DNA barcoding

The 658 bp fragment of the mitochondrial protein-encoding cytochrome c oxidase subunit I (COI) has been sequenced from a total of 46 *Coelophthinia* specimens on BOLD, 25 of them submitted by us during this study. One leg from each fresh specimen was sent to the Canadian Centre for DNA barcoding, BIO (Guelph, Ontario, Canada), for DNA extraction and bi-directional Sanger sequencing as a part of the Norwegian Barcode of Life (NorBOL) and Finnish Barcode of Life (FinBOL) initiatives, both branches of the International Barcode of Life project (iBOL). The new sequences are publicly available from The Barcode of Life Data System (BOLD) and referred to below with external links to their Barcode Index Numbers (BINs) on BOLD for each of the barcoded species.

### Mitogenomic analysis

**DNA extraction, library preparation and DNA sequencing**

One specimen of *C.loraasi* sp. n. (TSZD-JKJ-105417) was selected for further DNA sequencing. DNA was extracted from the whole individual, except its terminalia, using the E.Z.N.A. Insect DNA Kit (Omega bio-tek), following the manufacturer’s protocol. The terminalia was dissected and preserved in glycerine as voucher. DNA library preparation and sequencing were carried out at The Norwegian Sequencing Centre (NSC). The DNA sample was fragmented to a target of 350 bp average size. Library preparation was performed using the Kapa Hyper library prep kit (Roche), with cycles of PCR with the Kapa Library amplification mix (Roche) and two rounds of bead clean-up (both on PE Sciclone). The average fragment length after library preparation was measured to 538 bp. The samples were sequenced on the HiSeq 4000 system (Illumina inc.) with 150 bp paired-end sequencing following the manufacturer’s recommendations.

**Assembly and gene annotation**

Filtering of raw reads was carried out in Trimmomatic 0.39 (Bolger et al. 2014) with parameters MINLEN: 110 and SLIDINGWINDOW: 5:20. Filtered reads were error corrected using BayesHammer (Nikolenko et al. 2013) implemented in SPAdes v.3.15.0, before assembled in MEGAHIT v.1.1.4 (Li et al. 2015). A reference for the mitochondrial genome of *Acnemianitidicollis* (accession: NC_050318.1) was downloaded from GenBank. The assembly was searched against the reference using the *BLASTn* function from BLAST+ v.2.8.1 (Camacho et al. 2009). The mitochondrial region was obtained from the assembly as a single fragment and annotated using MITOS2 (Donath et al. 2019) on the MITOS web server. The tRNA trnH was annotated using tRNAscan-SE v.2.0.7 (Chan and Lowe 2019). The control region (CR) was identified as the longest sequence of intergeneric nucleotides. A map of the circular mitochondrial genome was created in OGDRAW (Greiner et al. 2019) on the OGDRAW server (https://chlorobox.mpimp-golm.mpg.de/OGDraw.html). The annotated genome of *C.loraasi* sp. n. is available in GenBank under the accession MZ853147.

## Taxon treatments

### Coelophthinia

Edwards, 1941

BEA6C6B1-2D37-5807-A461-2ACCC4DE0065

Coelophthinia Coelophthinia thoracica (Winnertz, 1864)

#### Description

A Gnoristinae genus with slender and medium-sized (2.8–4.3, 3.8 mm) species (Figs 1, 2a). Coloration quite uniformly brown, darker on head and preterminal, abdominal segments, three brown thoracic stripes distinctly contrasted against yellow humeral areas (Fig. 2b), abdominal tergites II-IV sometimes apically paler, legs and terminalia mostly yellow. Head (Fig. 2b, c) round, eyes slightly kidney-shaped, but without tendency of dorsal eye-bridge expansion, inter-ommatidia pubescent. Antenna moderately slender, with 16 segments, medium-sized, semi-globular scape and pedicel and flagellar segments 3-4 times as long as wide. Frons with greatly reduced, incomplete frontal furrow and relatively broad frontal tubercle (Fig. 2c). Mouthparts average, with five, gradually longer palpal segments, no clear sensory pit discernible in third segment (without slide mounting). Clypeus horseshoe-shaped, with scattered, long setae. Three ocelli in a near straight line, middle ocellus half the size of the lateral ocellus, lateral ocellus less than its diameter from eye. Thorax (Fig. 2a, b, e). Antepronotum with pair of medium-sized antepronotal setae. Mesonotum scattered with short setae, only moderately defined into rows, but rich in larger setae laterally. Mesopleurites without setae, mediotergite with row of strong setae basally. Wings (Fig. 2d) unpatterned hyaline with a slight brown tinge, with wing interference colours (Fig. 1, see Shevtsova et al. (2011)) in first order Newton colour scale, indicating very thin (less than 200 nanometres thickness) and even membrane beyond the wing base, wing membrane with irregularly arranged microtrichia. Costa produced slightly beyond to 1/4 between **R_4+5_** and **M_1_**, subcosta long, ending in **C** proximal to crossvein **Rs**, midway with crossvein **sc–r**. Radial sector with oblique crossvein **Rs**, without **R_2+3_**. Anterior fork long, with short stem subequal to **r–m**. Posterior fork short, widely divergent. Radial sector and both forks with setae on dorsal surface beyond base. Legs (Fig. 2a, e) with irregularly arranged setulae. Fore tarsus subequal in length to fore tibia, tarsal ratios can be extracted from Fig. 2e. A distinct sense organ present dorsally on the basal part of mid-tibia (Fig. 2e, f), of variable length between species.

Male terminalia (Fig. 3) apparently always positioned with a 180 degrees torsion in relation to abdomen (Fig. 1). Tergite 9 (Fig. 3a, c, d) short, wide rectangular, strongly setose, medially slightly constricted, with basal margin concave. Tergite 10 unusually well developed (Fig. 3c, d), with medial, densely setose, dorsally protrusive lobe and lateral extension armed with three strong setae. Hypoproct and cerci (Fig. 3a, c) forming elongate, basally fused lobes arising from underside of tergite 9, apically setose. Gonocoxites separated ventrally (Fig. 3b), strongly setose, except bare posterolateral lobes, which have one subapical, internal, medially directed small and stout seta (Fig. 3e). Setae on dorsal side of gonocoxite similar to those on ventral side. Posterolateral lobe of gonocoxite situated midway between dorsal and ventral edges (Fig. 3a, b, e), narrow, shape varies between species, always with one stiff, short seta apicointernally (Fig. 3e). Ventral medial margin of gonocoxite with a narrow, long, spathulate lobe (Fig. 3b, e), whose shape varies between species. Aedeagal guide (Fig. 3c) forming a knob with 3–5 medially directed setae deviating from other setae anteriorly on the base of the gonocoxal lobe. Gonostylus (Fig. 3b, e, f) small, internal with two branches, dorsal branch semicircular with normal setae, ventral branch bifurcated into two lobes (lb 1 & lb 2 in Fig. 3f), with truncated, blunt setae. Aedeagal apparatus (Fig. 3b, c) large, elongated, with strong, downcurved tip, basally attached to gonocoxite via strip like gonocoxal apodemes (Fig. 3c).

Female terminalia (Fig. 4) rather truncated, with short tergites 8 and 9. Tergite 9 wide, subrectangular, with some setae extending towards epiproct dorsally. Cercus one-segmented, large ovate, evenly covered with setae. Hypoproct/Tergite 10 forming narrow process along underside of cerci, with some setae. Gonocoxite 8 moderately split ventrally, with free, sclerotised, pointed lamellae. Sternite 9 small, retracted within terminalia.

#### Diagnosis

Males of the genus *Coelophthinia* are easily distinguished from all other Gnoristinae genera by the characteristic shape of their torsioned terminalia (Figs 1, 3), especially the open gonocoxite with long apicolateral and medioventral projections in combination with a small, internal gonostylus armed with a fan of blunt setae. The protrusive lobe of tergite 10 in the males (Fig. 3c, d) is a further unique characteristic for the genus. Both sexes can be distinguished from other Gnoristinae genera also by the wing venation (Fig. 2d) where the wide posterior fork is similar only to that of genus *Coelosia* Winnertz, 1864. From *Coelosia*, *Coelophthinia* differs in having crossvein **sc–r** present and in having a row of setae on the basal part of the mediotergite.

### Coelophthinia thoracica

(Winnertz, 1864)

F6351532-C8D3-59D0-8B5C-019C19F793CD

http://sciaroidea.info/taxonomy/41876

http://www.boldsystems.org/index.php/Public_BarcodeCluster?clusteruri=BOLD:ACJ0721

*Phthiniathoracica* Winnertz, 1964
*Coelosiathoracica* sensu Edwards (1925)
*Coelophthiniathoracica* sensu Edwards (1941)

#### Materials

- **Type status:** Other material. **Occurrence:** catalogNumber: TSZD-JKJ-106186; recordedBy: J. Kjærandsen, J. P. Lindemann & P. Dominiak; individualCount: 1; sex: female; lifeStage: imago; preparations: Pinned (HMDS-dried from ethanol); associatedOccurrences: urn:uuid:823656f0-abb9-4827-ae0e-8f26f9c86dcd; occurrenceID: 062469D4-2D81-5D69-BF10-418C05D439D7; **Taxon:** scientificName: *Coelophthiniathoracica*; order: Diptera; family: Mycetophilidae; genus: Coelophthinia; specificEpithet: *thoracica*; scientificNameAuthorship: (Winnertz); **Location:** country: Norway; stateProvince: Nordland (NSI); municipality: Hattfjelldal; locality: Auster-Vefsna NR, Stilleelva E; decimalLatitude: 65.54083; decimalLongitude: 13.75028; coordinateUncertaintyInMeters: 10; **Identification:** identifiedBy: J. Kjærandsen; dateIdentified: Feb-11-2019; **Event:** samplingProtocol: Malaise trap; eventDate: 2018-08-02 to 2018-10-05; eventRemarks: MT 6; **Record Level:** institutionCode: TSZ; collectionCode: TMU-JKJ-COL-000807; basisOfRecord: Preserved specimen
- **Type status:** Other material. **Occurrence:** catalogNumber: TSZD-JKJ-106504; recordedBy: J. Kjærandsen, J. P. Lindemann & P. Dominiak; individualCount: 1; sex: female; lifeStage: imago; preparations: Pinned (HMDS-dried from ethanol); associatedOccurrences: urn:uuid:fcf4435f-ca39-42db-b6f7-0631601c13d5; occurrenceID: 84BAD5D4-F4B8-521A-B762-1916DDC301DA; **Taxon:** scientificName: *Coelophthiniathoracica*; order: Diptera; family: Mycetophilidae; genus: Coelophthinia; specificEpithet: *thoracica*; scientificNameAuthorship: (Winnertz); **Location:** country: Norway; stateProvince: Nordland (NSI); municipality: Grane; locality: Holmvassdalen NR, riparian forest at Holmvasselva; decimalLatitude: 65.33556; decimalLongitude: 13.32222; coordinateUncertaintyInMeters: 10; **Identification:** identifiedBy: J. Kjærandsen; dateIdentified: Feb-14-2019; **Event:** samplingProtocol: window trap; eventDate: 2018-05-30 to 2018-08-01; eventRemarks: WT 4; **Record Level:** institutionCode: TSZ; collectionCode: TMU-JKJ-COL-000637; basisOfRecord: Preserved specimen
- **Type status:** Other material. **Occurrence:** catalogNumber: TSZD-JKJ-105793; recordedBy: J. Kjærandsen, J. P. Lindemann & P. Dominiak; individualCount: 1; sex: female; lifeStage: imago; preparations: Pinned (HMDS-dried) + terminalia in glycerine microvial; associatedOccurrences: urn:uuid:b87d27c6-a46d-4ccc-ab44-43396323b648; occurrenceID: B43CE1FF-B067-59C8-A78F-35B5BE8C5D11; **Taxon:** scientificName: *Coelophthiniathoracica*; order: Diptera; family: Mycetophilidae; genus: Coelophthinia; specificEpithet: *thoracica*; scientificNameAuthorship: (Winnertz); **Location:** country: Norway; stateProvince: Nordland (NSI); municipality: Grane; locality: Stormobekken; decimalLatitude: 65.595; decimalLongitude: 13.40306; coordinateUncertaintyInMeters: 10; **Identification:** identifiedBy: J. Kjærandsen; dateIdentified: Jan-25-2019; **Event:** samplingProtocol: Malaise trap; eventDate: 2018-07-31 to 2018-10-05; eventRemarks: MT 3; **Record Level:** institutionCode: TSZ; collectionCode: TMU-JKJ-COL-000798; basisOfRecord: Preserved specimen
- **Type status:** Other material. **Occurrence:** catalogNumber: TSZD-JKJ-105815; recordedBy: J. Kjærandsen, J. P. Lindemann & P. Dominiak; individualCount: 1; sex: male; lifeStage: imago; preparations: Pinned (HMDS-dried) + terminalia in glycerine microvial; associatedOccurrences: urn:uuid:68f4dfed-5c50-4b47-a76e-9bf14ba1c0ee; occurrenceID: F1F05E45-FB6C-5C37-9A8F-930E3FD8A030; **Taxon:** scientificName: *Coelophthiniathoracica*; order: Diptera; family: Mycetophilidae; genus: Coelophthinia; specificEpithet: *thoracica*; scientificNameAuthorship: (Winnertz); **Location:** country: Norway; stateProvince: Nordland (NSI); municipality: Grane; locality: Stormobekken; decimalLatitude: 65.595; decimalLongitude: 13.40306; coordinateUncertaintyInMeters: 10; **Identification:** identifiedBy: J. Kjærandsen; dateIdentified: Jan-25-2019; **Event:** samplingProtocol: Malaise trap; eventDate: 2018-07-31 to 2018-10-05; eventRemarks: MT 3; **Record Level:** institutionCode: TSZ; collectionCode: TMU-JKJ-COL-000798; basisOfRecord: Preserved specimen
- **Type status:** Other material. **Occurrence:** catalogNumber: TSZD-JKJ-105837; recordedBy: J. Kjærandsen, J. P. Lindemann & P. Dominiak; individualCount: 1; sex: male; lifeStage: imago; preparations: Pinned (HMDS-dried) + terminalia in glycerine microvial; associatedOccurrences: urn:uuid:f7596cdd-0996-4c71-9875-0d87f6ffb4ff; occurrenceID: 9DEAB8B1-E427-5215-891D-B9843C6BA1E8; **Taxon:** scientificName: *Coelophthiniathoracica*; order: Diptera; family: Mycetophilidae; genus: Coelophthinia; specificEpithet: *thoracica*; scientificNameAuthorship: (Winnertz); **Location:** country: Norway; stateProvince: Nordland (NSI); municipality: Grane; locality: Stormobekken; decimalLatitude: 65.595; decimalLongitude: 13.40333; coordinateUncertaintyInMeters: 10; **Identification:** identifiedBy: J. Kjærandsen; dateIdentified: Jan-28-2019; **Event:** samplingProtocol: window trap; eventDate: 2018-07-31 to 2018-10-05; eventRemarks: WT 3; **Record Level:** institutionCode: TSZ; collectionCode: TMU-JKJ-COL-000799; basisOfRecord: Preserved specimen
- **Type status:** Other material. **Occurrence:** catalogNumber: TSZD-JKJ-106712; recordedBy: J. Kjærandsen, J. P. Lindemann & P. Dominiak; individualCount: 1; sex: male; lifeStage: imago; preparations: Pinned (HMDS-dried) + terminalia in glycerine microvial; associatedOccurrences: urn:uuid:5aeec5c8-908d-4d1a-9825-dc9dbf8e50bf; occurrenceID: 40190A39-45C9-5D1A-9339-F319C515DACD; **Taxon:** scientificName: *Coelophthiniathoracica*; order: Diptera; family: Mycetophilidae; genus: Coelophthinia; specificEpithet: *thoracica*; scientificNameAuthorship: (Winnertz); **Location:** country: Norway; stateProvince: Nordland (NSI); municipality: Grane; locality: Stormobekken; decimalLatitude: 65.595; decimalLongitude: 13.40333; coordinateUncertaintyInMeters: 10; **Identification:** identifiedBy: J. Kjærandsen; dateIdentified: Feb-20-2019; **Event:** samplingProtocol: window trap; eventDate: 2018-07-31 to 2018-10-05; eventRemarks: WT 3; **Record Level:** institutionCode: TSZ; collectionCode: TMU-JKJ-COL-000799; basisOfRecord: Preserved specimen
- **Type status:** Other material. **Occurrence:** catalogNumber: TSZD-JKJ-106720; recordedBy: J. Kjærandsen, J. P. Lindemann & P. Dominiak; individualCount: 1; sex: male; lifeStage: imago; preparations: Pinned (HMDS-dried) + terminalia in glycerine microvial; associatedOccurrences: urn:uuid:30033165-bbc5-4d2e-b028-68e66ff88cc3; occurrenceID: 7AA75332-A497-5CDE-BF04-F1A04C03D8C0; **Taxon:** scientificName: *Coelophthiniathoracica*; order: Diptera; family: Mycetophilidae; genus: Coelophthinia; specificEpithet: *thoracica*; scientificNameAuthorship: (Winnertz); **Location:** country: Norway; stateProvince: Nordland (NSI); municipality: Grane; locality: Stormobekken; decimalLatitude: 65.595; decimalLongitude: 13.40333; coordinateUncertaintyInMeters: 10; **Identification:** identifiedBy: J. Kjærandsen; dateIdentified: Feb-20-2019; **Event:** samplingProtocol: window trap; eventDate: 2018-07-31 to 2018-10-05; eventRemarks: WT 3; **Record Level:** institutionCode: TSZ; collectionCode: TMU-JKJ-COL-000799; basisOfRecord: Preserved specimen
- **Type status:** Other material. **Occurrence:** catalogNumber: TSZD-JKJ-106721; recordedBy: J. Kjærandsen, J. P. Lindemann & P. Dominiak; individualCount: 1; sex: male; lifeStage: imago; preparations: Pinned (HMDS-dried) + terminalia in glycerine microvial; associatedOccurrences: urn:uuid:8c3a90fb-0ece-44e0-9514-75075f2dff30; occurrenceID: CEDF7D02-9AB8-57B2-9447-F854BB49B63D; **Taxon:** scientificName: *Coelophthiniathoracica*; order: Diptera; family: Mycetophilidae; genus: Coelophthinia; specificEpithet: *thoracica*; scientificNameAuthorship: (Winnertz); **Location:** country: Norway; stateProvince: Nordland (NSI); municipality: Grane; locality: Stormobekken; decimalLatitude: 65.595; decimalLongitude: 13.40333; coordinateUncertaintyInMeters: 10; **Identification:** identifiedBy: J. Kjærandsen; dateIdentified: Feb-20-2019; **Event:** samplingProtocol: window trap; eventDate: 2018-07-31 to 2018-10-05; eventRemarks: WT 3; **Record Level:** institutionCode: TSZ; collectionCode: TMU-JKJ-COL-000799; basisOfRecord: Preserved specimen
- **Type status:** Other material. **Occurrence:** catalogNumber: TSZD-JKJ-106722; recordedBy: J. Kjærandsen, J. P. Lindemann & P. Dominiak; individualCount: 1; sex: male; lifeStage: imago; preparations: Pinned (HMDS-dried) + terminalia in glycerine microvial; associatedOccurrences: urn:uuid:ac95fe77-bc60-4329-9434-0233eb8d3160; occurrenceID: B8F4B96F-27A2-553E-9C4C-523EBC78461C; **Taxon:** scientificName: *Coelophthiniathoracica*; order: Diptera; family: Mycetophilidae; genus: Coelophthinia; specificEpithet: *thoracica*; scientificNameAuthorship: (Winnertz); **Location:** country: Norway; stateProvince: Nordland (NSI); municipality: Grane; locality: Stormobekken; decimalLatitude: 65.595; decimalLongitude: 13.40333; coordinateUncertaintyInMeters: 10; **Identification:** identifiedBy: J. Kjærandsen; dateIdentified: Feb-20-2019; **Event:** samplingProtocol: window trap; eventDate: 2018-07-31 to 2018-10-05; eventRemarks: WT 3; **Record Level:** institutionCode: TSZ; collectionCode: TMU-JKJ-COL-000799; basisOfRecord: Preserved specimen
- **Type status:** Other material. **Occurrence:** catalogNumber: TSZD-JKJ-106723; recordedBy: J. Kjærandsen, J. P. Lindemann & P. Dominiak; individualCount: 1; sex: female; lifeStage: imago; preparations: Pinned (HMDS-dried) + terminalia in glycerine microvial; associatedOccurrences: urn:uuid:388f25e3-58a2-4d5f-9dc5-8a48aa746a24; occurrenceID: 8E8AE664-B606-572E-9797-3130C28757E8; **Taxon:** scientificName: *Coelophthiniathoracica*; order: Diptera; family: Mycetophilidae; genus: Coelophthinia; specificEpithet: *thoracica*; scientificNameAuthorship: (Winnertz); **Location:** country: Norway; stateProvince: Nordland (NSI); municipality: Grane; locality: Stormobekken; decimalLatitude: 65.595; decimalLongitude: 13.40333; coordinateUncertaintyInMeters: 10; **Identification:** identifiedBy: J. Kjærandsen; dateIdentified: Feb-20-2019; **Event:** samplingProtocol: window trap; eventDate: 2018-07-31 to 2018-10-05; eventRemarks: WT 3; **Record Level:** institutionCode: TSZ; collectionCode: TMU-JKJ-COL-000799; basisOfRecord: Preserved specimen
- **Type status:** Other material. **Occurrence:** catalogNumber: TSZD-JKJ-211962; recordedBy: J. B. Jordal; individualCount: 1; sex: male; lifeStage: imago; preparations: Pinned (HMDS-dried) + terminalia in glycerine microvial; associatedOccurrences: urn:uuid:f1a7f914-3e68-41ea-8ddc-24aacf96b03f; occurrenceID: 665866ED-AD67-5E05-A4D1-A1667AD557E2; **Taxon:** scientificName: *Coelophthiniathoracica*; order: Diptera; family: Mycetophilidae; genus: Coelophthinia; specificEpithet: *thoracica*; scientificNameAuthorship: (Winnertz); **Location:** country: Norway; stateProvince: Møre og Romsdal (MRI); municipality: Sunndal; locality: Jordalsgrenda, Jordalsøra, Hamrene; minimumElevationInMeters: 140; decimalLatitude: 62.77167; decimalLongitude: 8.32; coordinateUncertaintyInMeters: 50; **Identification:** identifiedBy: J. Kjærandsen; dateIdentified: Jun-21-2021; **Event:** samplingProtocol: window trap; eventDate: 2005-08-25 to 2005-09-15; **Record Level:** institutionCode: TSZ; collectionCode: COL-002881; basisOfRecord: Preserved specimen
- **Type status:** Other material. **Occurrence:** catalogNumber: TSZD-JKJ-211963; recordedBy: J. B. Jordal; individualCount: 1; sex: female; lifeStage: imago; preparations: Pinned (HMDS-dried) + terminalia in glycerine microvial; associatedOccurrences: urn:uuid:375b90a8-3770-418c-874e-6a1da807df11; occurrenceID: E6F76F3E-D289-5F67-8F47-8AB244027381; **Taxon:** scientificName: *Coelophthiniathoracica*; order: Diptera; family: Mycetophilidae; genus: Coelophthinia; specificEpithet: *thoracica*; scientificNameAuthorship: (Winnertz); **Location:** country: Norway; stateProvince: Møre og Romsdal (MRI); municipality: Sunndal; locality: Jordalsgrenda, Jordalsøra, Hamrene; minimumElevationInMeters: 140; decimalLatitude: 62.77167; decimalLongitude: 8.32; coordinateUncertaintyInMeters: 50; **Identification:** identifiedBy: J. Kjærandsen; dateIdentified: Jun-21-2021; **Event:** samplingProtocol: window trap; eventDate: 2005-08-25 to 2005-09-15; **Record Level:** institutionCode: TSZ; collectionCode: COL-002881; basisOfRecord: Preserved specimen
- **Type status:** Other material. **Occurrence:** catalogNumber: TSZD-JKJ-212829; recordedBy: J. B. Jordal; individualCount: 1; sex: female; lifeStage: imago; preparations: 80% alc.; associatedOccurrences: urn:uuid:0c44d198-d721-468f-ad0b-618c82fa7aa3; occurrenceID: D8394023-A8C5-5879-AEFA-FB057E6FBEC9; **Taxon:** scientificName: *Coelophthiniathoracica*; order: Diptera; family: Mycetophilidae; genus: Coelophthinia; specificEpithet: *thoracica*; scientificNameAuthorship: (Winnertz); **Location:** country: Norway; stateProvince: Møre og Romsdal (MRI); municipality: Sunndal; locality: Jordalsgrenda, Jordalsøra, Hamrene; minimumElevationInMeters: 140; decimalLatitude: 62.77167; decimalLongitude: 8.32; coordinateUncertaintyInMeters: 50; **Identification:** identifiedBy: J. Kjærandsen; dateIdentified: Feb-10-2006; **Event:** samplingProtocol: window trap; eventDate: 2005-09-15 to 2005-10-06; **Record Level:** institutionCode: TSZ; collectionCode: COL-002882; basisOfRecord: Preserved specimen
- **Type status:** Other material. **Occurrence:** catalogNumber: TSZD-JKJ-213004; recordedBy: J. B. Jordal; individualCount: 1; sex: female; lifeStage: imago; preparations: 80% alc.; associatedOccurrences: urn:uuid:f213ef59-8ba3-4be4-a647-c5f8333d6d30; occurrenceID: D965A3C9-7598-50DA-8A19-EFA1BCB5187B; **Taxon:** scientificName: *Coelophthiniathoracica*; order: Diptera; family: Mycetophilidae; genus: Coelophthinia; specificEpithet: *thoracica*; scientificNameAuthorship: (Winnertz); **Location:** country: Norway; stateProvince: Møre og Romsdal (MRI); municipality: Sunndal; locality: Jordalsgrenda, Jordalsøra, Hamrene; minimumElevationInMeters: 140; decimalLatitude: 62.77167; decimalLongitude: 8.32; coordinateUncertaintyInMeters: 5; **Identification:** identifiedBy: J. Kjærandsen; dateIdentified: Mar-28-2006; **Event:** samplingProtocol: window trap; eventDate: 2005-10-06 to 2005-10-23; **Record Level:** institutionCode: TSZ; collectionCode: COL-002903; basisOfRecord: Preserved specimen
- **Type status:** Other material. **Occurrence:** catalogNumber: TSZD-JKJ-215439; recordedBy: J. B. Jordal; individualCount: 1; sex: female; lifeStage: imago; preparations: 80% alc.; associatedOccurrences: urn:uuid:717ce56d-3141-4e11-a0f5-ea68b5ece1a7; occurrenceID: D5416AD5-8A81-5A30-A95C-970ADF5FC2FF; **Taxon:** scientificName: *Coelophthiniathoracica*; order: Diptera; family: Mycetophilidae; genus: Coelophthinia; specificEpithet: *thoracica*; scientificNameAuthorship: (Winnertz); **Location:** country: Norway; stateProvince: Møre og Romsdal (MRI); municipality: Sunndal; locality: Jordalsgrenda, Jordalsøra, Hamrene; minimumElevationInMeters: 140; decimalLatitude: 62.77167; decimalLongitude: 8.32; coordinateUncertaintyInMeters: 50; **Identification:** identifiedBy: J. Kjærandsen; dateIdentified: Apr-28-2007; **Event:** samplingProtocol: Malaisetrap; eventDate: 2006-10-06 to 2006-10-23; **Record Level:** institutionCode: TSZ; collectionCode: COL-003343; basisOfRecord: Preserved specimen
- **Type status:** Other material. **Occurrence:** catalogNumber: TSZD-JKJ-258439; recordedBy: J. B. Jordal; individualCount: 1; sex: male; lifeStage: imago; preparations: Pinned body+Term. on slide; associatedOccurrences: urn:uuid:01556e8c-6261-4762-9e7d-6a5a334339e6; occurrenceID: BFD6E0EE-0486-5C05-BFC1-67ADDD457F35; **Taxon:** scientificName: *Coelophthiniathoracica*; order: Diptera; family: Mycetophilidae; genus: Coelophthinia; specificEpithet: *thoracica*; scientificNameAuthorship: (Winnertz); **Location:** country: Norway; stateProvince: Møre og Romsdal (MRI); municipality: Sunndal; locality: Jordalsgrenda, Jordalsøra, Hamrene; minimumElevationInMeters: 140; decimalLatitude: 62.77167; decimalLongitude: 8.32; coordinateUncertaintyInMeters: 50; **Identification:** identifiedBy: J. Kjærandsen; dateIdentified: Nov-19-2005; **Event:** samplingProtocol: window trap; eventDate: 2005-08-25 to 2005-09-15; **Record Level:** institutionCode: TSZ; collectionCode: COL-002881; basisOfRecord: Preserved specimen
- **Type status:** Other material. **Occurrence:** catalogNumber: TSZD-JKJ-111214; recordedBy: J. Kjærandsen; individualCount: 1; sex: male; lifeStage: imago; preparations: Pinned (HMDS) + term in glycerine; associatedOccurrences: urn:uuid:85481bfd-bf00-4f48-8ada-0e29cf4cf501; occurrenceID: 6909E658-B3CB-552C-BA18-831EF2E7AB7B; **Taxon:** scientificName: *Coelophthiniathoracica*; order: Diptera; family: Mycetophilidae; genus: Coelophthinia; specificEpithet: *thoracica*; scientificNameAuthorship: (Winnertz); **Location:** country: Norway; stateProvince: Vestland (HOY); municipality: Sveio; locality: Førde, Solheimshaugen, in barn; minimumElevationInMeters: 35; decimalLatitude: 59.61505; decimalLongitude: 5.47556; coordinateUncertaintyInMeters: 10; **Identification:** identifiedBy: J. Kjærandsen; dateIdentified: Aug-04-2020; **Event:** samplingProtocol: pooter; eventDate: 07/26/2020; eventRemarks: in barn window post; **Record Level:** institutionCode: TSZ - BOLD voucher; collectionCode: TMU-JKJ-COL-001476; basisOfRecord: Preserved specimen
- **Type status:** Other material. **Occurrence:** catalogNumber: TSZD-JKJ-207257; recordedBy: A. Fjeldså; individualCount: 1; sex: male; lifeStage: imago; preparations: Ethanol (80%); associatedOccurrences: urn:uuid:b024d273-f7d6-46da-ac7c-66804fa224bb; occurrenceID: 3569311D-E6DA-58C0-82C7-7279A2E8D61E; **Taxon:** scientificName: *Coelophthiniathoracica*; order: Diptera; family: Mycetophilidae; genus: Coelophthinia; specificEpithet: *thoracica*; scientificNameAuthorship: (Winnertz); **Location:** country: Norway; stateProvince: Vestland (HOY); municipality: Osterøy; locality: Kleppe; decimalLatitude: 60.52579; decimalLongitude: 5.55333; coordinateUncertaintyInMeters: 500; **Identification:** identifiedBy: J. Kjærandsen; dateIdentified: Sep-21-2004; **Event:** samplingProtocol: yellow traps; eventDate: 1992-10-10 to 1992-10-31; **Record Level:** institutionCode: TSZ [transferred from MZLU 2014]; collectionCode: COL-001708; basisOfRecord: Preserved specimen
- **Type status:** Other material. **Occurrence:** catalogNumber: TSZD-JKJ-107949; recordedBy: J. Birkeland; individualCount: 1; sex: male; lifeStage: imago; preparations: Pinned (HMDS-dried from ethanol); associatedOccurrences: urn:uuid:9454a831-252b-4192-a00c-677cef5f23c2; occurrenceID: DBA8E29D-03E4-51FA-B368-1A6FD31C1030; **Taxon:** scientificName: *Coelophthiniathoracica*; order: Diptera; family: Mycetophilidae; genus: Coelophthinia; specificEpithet: *thoracica*; scientificNameAuthorship: (Winnertz); **Location:** country: Norway; stateProvince: Rogaland (RY); municipality: Sokndal; locality: Skitmyr; minimumElevationInMeters: 22; decimalLatitude: 58.35056; decimalLongitude: 6.30556; coordinateUncertaintyInMeters: 50; **Identification:** identifiedBy: J. Kjærandsen; dateIdentified: May-25-2020; **Event:** samplingProtocol: Malaise trap; eventDate: 2019-09-07 to 2019-10-27; **Record Level:** institutionCode: TSZ - BOLD voucher; collectionCode: TMU-JKJ-COL-001079; basisOfRecord: Preserved specimen
- **Type status:** Other material. **Occurrence:** catalogNumber: TSZD-JKJ-111485; recordedBy: J. Birkeland; individualCount: 1; sex: female; lifeStage: imago; preparations: Pinned (HMDS-dried from ethanol); associatedOccurrences: urn:uuid:4c4cc150-575a-440b-9800-e2c1a8d578c0; occurrenceID: F043D0C5-3FA0-5D6C-86FB-0E5A9408B653; **Taxon:** scientificName: *Coelophthiniathoracica*; order: Diptera; family: Mycetophilidae; genus: Coelophthinia; specificEpithet: *thoracica*; scientificNameAuthorship: (Winnertz); **Location:** country: Norway; stateProvince: Rogaland (RY); municipality: Sokndal; locality: Årstad; minimumElevationInMeters: 6; decimalLatitude: 58.33806; decimalLongitude: 6.3; coordinateUncertaintyInMeters: 10; **Identification:** identifiedBy: J. Kjærandsen; dateIdentified: Oct-10-2020; **Event:** samplingProtocol: Malaise trap; eventDate: 2020-08-09 to 2020-09-05; **Record Level:** institutionCode: TSZ - BOLD voucher; collectionCode: TMU-JKJ-COL-001549; basisOfRecord: Preserved specimen
- **Type status:** Other material. **Occurrence:** catalogNumber: TSZD-JKJ-259894; recordedBy: M. Lindström; individualCount: 1; sex: female; lifeStage: imago; preparations: Pinned (HMDS-dried) + terminalia in glycerine microvial; associatedOccurrences: urn:uuid:ef86403f-2c21-41df-ae4b-17ea4bc13333; occurrenceID: 4FD961DD-D5A5-5F4F-8B3B-1986FB65F203; **Taxon:** scientificName: *Coelophthiniathoracica*; order: Diptera; family: Mycetophilidae; genus: Coelophthinia; specificEpithet: *thoracica*; scientificNameAuthorship: (Winnertz); **Location:** country: Sweden; stateProvince: Halland (HA); municipality: Halmstads kommun.; locality: Biskopstorp S; decimalLatitude: 56.78444; decimalLongitude: 12.87; coordinateUncertaintyInMeters: 50; **Identification:** identifiedBy: J. Kjærandsen; dateIdentified: Apr-09-2013; **Event:** samplingProtocol: Malaise trap; eventDate: 2011-05-15 to 2011-07-16; **Record Level:** institutionCode: TSZ [transferred from MZLU 2014]; collectionCode: COL-009388; basisOfRecord: Preserved specimen
- **Type status:** Other material. **Occurrence:** catalogNumber: SPM-008187; recordedBy: R. Rova; individualCount: 4; sex: males; lifeStage: imago; preparations: 80% alc.; associatedOccurrences: urn:uuid:7d2e875a-da11-45e8-abfc-33897aeb3073; occurrenceID: 0CD3DEC2-BD54-55EE-A7C0-C535595AFC97; **Taxon:** scientificName: *Coelophthiniathoracica*; order: Diptera; family: Mycetophilidae; genus: Coelophthinia; specificEpithet: *thoracica*; scientificNameAuthorship: (Winnertz); **Location:** country: Sweden; stateProvince: Norrbottens län (LU); municipality: Gällivare; locality: Haapavaara/Annavaara, 8 km WNW Vettasjärvi; decimalLatitude: 67.40622; decimalLongitude: 21.406111; coordinateUncertaintyInMeters: 1000; **Identification:** identifiedBy: J. Kjærandsen; dateIdentified: Feb-10-2005; **Event:** samplingProtocol: yellow pan traps; eventDate: 1994-06-01 to 1994-07-26; eventRemarks: Burk 21; **Record Level:** institutionCode: NHRS; collectionCode: COL-002153; basisOfRecord: Preserved specimen
- **Type status:** Other material. **Occurrence:** catalogNumber: TSZD-JKJ-216023; recordedBy: J. Kjærandsen; individualCount: 1; sex: male; lifeStage: imago; preparations: DNA-voucher, cleared terminalia in glycerine; associatedOccurrences: urn:uuid:4b524878-0d9b-4eea-8d4d-3c03ee4bc12a; occurrenceID: 5D54D99F-8DA5-5F68-907E-BF8F3C6BC0FC; **Taxon:** scientificName: *Coelophthiniathoracica*; order: Diptera; family: Mycetophilidae; genus: Coelophthinia; specificEpithet: *thoracica*; scientificNameAuthorship: (Winnertz); **Location:** country: Sweden; stateProvince: Skåne (SK); municipality: Genarp; locality: Häckeberga; decimalLatitude: 55.593056; decimalLongitude: 13.425556; coordinateUncertaintyInMeters: 100; **Identification:** identifiedBy: J. Kjærandsen; dateIdentified: Aug-27-2007; **Event:** samplingProtocol: sweep net; eventDate: 08/26/2007; eventRemarks: East of Genarp idrettsplass; **Record Level:** institutionCode: TSZ [transferred from MZLU 2014]; collectionCode: COL-004141; basisOfRecord: Preserved specimen
- **Type status:** Other material. **Occurrence:** catalogNumber: SPM-010078; recordedBy: Swedish Malaise Trap Project, NHRS; individualCount: 1; sex: male; lifeStage: imago; preparations: 80% alc.; associatedOccurrences: urn:uuid:165b5596-10a7-4603-b311-794effd00996; occurrenceID: 1F7FD0BE-6BEB-565A-92C5-7253E1183C6E; **Taxon:** scientificName: *Coelophthiniathoracica*; order: Diptera; family: Mycetophilidae; genus: Coelophthinia; specificEpithet: *thoracica*; scientificNameAuthorship: (Winnertz); **Location:** country: Sweden; stateProvince: Stockholms län (SÖ); municipality: Haninge; locality: Tyresta, Urskogsslingan, granskog; decimalLatitude: 59.1759; decimalLongitude: 18.24758; coordinateUncertaintyInMeters: 10; **Identification:** identifiedBy: J. Kjærandsen; dateIdentified: Jun-20-2005; **Event:** samplingProtocol: Malaise trap; eventDate: 2003-07-02 to 2003-07-21; eventRemarks: trap id. 4-88; **Record Level:** institutionCode: NHRS; collectionCode: SMTP-0088; basisOfRecord: Preserved specimen
- **Type status:** Other material. **Occurrence:** catalogNumber: SPM-010199; recordedBy: B. Viklund, L. O. Wikars & H. Ahnlund; individualCount: 8; sex: males; lifeStage: imago; preparations: 80% alc.; associatedOccurrences: urn:uuid:d658f27b-9750-4e83-bd83-f9eee6c56bde; occurrenceID: 9D1B95B1-A2AA-58EB-A73C-53762B8E4829; **Taxon:** scientificName: *Coelophthiniathoracica*; order: Diptera; family: Mycetophilidae; genus: Coelophthinia; specificEpithet: *thoracica*; scientificNameAuthorship: (Winnertz); **Location:** country: Sweden; stateProvince: Stockholms län (SÖ); municipality: Haninge; locality: Tyresta National Park; decimalLatitude: 59.18639; decimalLongitude: 18.30528; coordinateUncertaintyInMeters: 1000; **Identification:** identifiedBy: J. Kjærandsen; dateIdentified: Jun-24-2005; **Event:** samplingProtocol: Yellow traps; eventDate: 2000-07-28 to 2000-09-20; eventRemarks: Site 07; **Record Level:** institutionCode: NHRS; collectionCode: COL-002625; basisOfRecord: Preserved specimen
- **Type status:** Other material. **Occurrence:** catalogNumber: SPM-010216; recordedBy: B. Viklund, L. O. Wikars & H. Ahnlund; individualCount: 1; sex: female; lifeStage: imago; preparations: 80% alc.; associatedOccurrences: urn:uuid:5309c85b-5f22-4f68-9f22-f6051976340c; occurrenceID: 9C5722E4-C041-5F8B-ABFF-2D62C5A502B4; **Taxon:** scientificName: *Coelophthiniathoracica*; order: Diptera; family: Mycetophilidae; genus: Coelophthinia; specificEpithet: *thoracica*; scientificNameAuthorship: (Winnertz); **Location:** country: Sweden; stateProvince: Stockholms län (SÖ); municipality: Haninge; locality: Tyresta National Park; decimalLatitude: 59.18639; decimalLongitude: 18.30528; coordinateUncertaintyInMeters: 1000; **Identification:** identifiedBy: J. Kjærandsen; dateIdentified: Jun-24-2005; **Event:** samplingProtocol: Yellow traps; eventDate: 2000-07-28 to 2000-09-20; eventRemarks: Site 07; **Record Level:** institutionCode: NHRS; collectionCode: COL-002624; basisOfRecord: Preserved specimen
- **Type status:** Other material. **Occurrence:** catalogNumber: SPM-010222; recordedBy: B. Viklund, L. O. Wikars & H. Ahnlund; individualCount: 1; sex: male; lifeStage: imago; preparations: 80% alc.; associatedOccurrences: urn:uuid:f477d532-e63b-4b55-848c-0a5e59e0a288; occurrenceID: F75A619F-FCE2-5892-AABB-333BC0C1F1CA; **Taxon:** scientificName: *Coelophthiniathoracica*; order: Diptera; family: Mycetophilidae; genus: Coelophthinia; specificEpithet: *thoracica*; scientificNameAuthorship: (Winnertz); **Location:** country: Sweden; stateProvince: Stockholms län (SÖ); municipality: Haninge; locality: Tyresta National Park; decimalLatitude: 59.18639; decimalLongitude: 18.30528; coordinateUncertaintyInMeters: 1000; **Identification:** identifiedBy: J. Kjærandsen; dateIdentified: Jun-24-2005; **Event:** samplingProtocol: Yellow traps; eventDate: 2000-07-28 to 2000-09-20; eventRemarks: Site 07; **Record Level:** institutionCode: NHRS; collectionCode: COL-002626; basisOfRecord: Preserved specimen
- **Type status:** Other material. **Occurrence:** catalogNumber: SPM-010679; recordedBy: B. Viklund, L. O. Wikars & H. Ahnlund; individualCount: 1; sex: male; lifeStage: imago; preparations: 80% alc.; associatedOccurrences: urn:uuid:047bbcca-d09a-460d-9518-514fe6f46602; occurrenceID: 4BCE3817-F8D2-54B7-B8CA-A9E09CFE0F54; **Taxon:** scientificName: *Coelophthiniathoracica*; order: Diptera; family: Mycetophilidae; genus: Coelophthinia; specificEpithet: *thoracica*; scientificNameAuthorship: (Winnertz); **Location:** country: Sweden; stateProvince: Stockholms län (SÖ); municipality: Haninge; locality: Tyresta National Park; decimalLatitude: 59.18639; decimalLongitude: 18.30528; coordinateUncertaintyInMeters: 1000; **Identification:** identifiedBy: J. Jakovlev; dateIdentified: Jun-08-2005; **Event:** samplingProtocol: Malaise trap; eventDate: 2000-06-05 to 2000-07-15; eventRemarks: Site 04; **Record Level:** institutionCode: NHRS; collectionCode: COL-T-04BA00; basisOfRecord: Preserved specimen
- **Type status:** Other material. **Occurrence:** catalogNumber: SPM-015937; recordedBy: Swedish Malaise Trap Project, NHRS; individualCount: 1; sex: male; lifeStage: imago; preparations: 80% alc.; associatedOccurrences: urn:uuid:f2cb03c4-0e35-4d84-9e58-414780e77187; occurrenceID: 5896C867-2940-54B1-A0AE-AF44BB6E993F; **Taxon:** scientificName: *Coelophthiniathoracica*; order: Diptera; family: Mycetophilidae; genus: Coelophthinia; specificEpithet: *thoracica*; scientificNameAuthorship: (Winnertz); **Location:** country: Sweden; stateProvince: Östergötlands län (ÖG); municipality: Ödeshögs kommun; locality: Omberg, Storpissan; decimalLatitude: 58.33491667; decimalLongitude: 14.655; coordinateUncertaintyInMeters: 50; **Identification:** identifiedBy: J. Kjærandsen; dateIdentified: Jul-23-2007; **Event:** samplingProtocol: Malaise trap; eventDate: 2005-03-03 to 2005-05-28; eventRemarks: trap id. 15-1658; **Record Level:** institutionCode: NHRS; collectionCode: SMTP-1658; basisOfRecord: Preserved specimen
- **Type status:** Other material. **Occurrence:** catalogNumber: TSZD-JKJ-215250; recordedBy: T. Munk & S. Kjeldgaard; individualCount: 1; sex: male; lifeStage: imago; preparations: 80% alc.; associatedOccurrences: urn:uuid:c243a63b-72be-46a6-9937-56f2a29afef9; occurrenceID: 0859DB2A-56E5-55C4-864E-4FB710F3C9DD; **Taxon:** scientificName: *Coelophthiniathoracica*; order: Diptera; family: Mycetophilidae; genus: Coelophthinia; specificEpithet: *thoracica*; scientificNameAuthorship: (Winnertz); **Location:** country: Denmark; stateProvince: East Jutland (EJ); municipality: Norddjurs; locality: Anholdt; decimalLatitude: 56.70978; decimalLongitude: 11.56551; coordinateUncertaintyInMeters: 4000; **Identification:** identifiedBy: J. Kjærandsen; dateIdentified: Dec-14-2006; **Event:** samplingProtocol: Malaise trap; eventDate: 2006-09-24 to 2006-11-10; eventRemarks: in Pinus scrub; **Record Level:** institutionCode: TSZ; collectionCode: COL-003311; basisOfRecord: Preserved specimen
- **Type status:** Other material. **Occurrence:** catalogNumber: IZBE0251600; recordedBy: Ilmar Süda; individualCount: 1; sex: male; lifeStage: imago; preparations: specimen in 70% ethanol; associatedOccurrences: BDJ_12308_1; occurrenceID: DB662280-BD4A-5821-8E2D-BD2983F55862; **Taxon:** scientificName: *Coelophthiniathoracica*; order: Diptera; family: Mycetophilidae; genus: Coelophthinia; specificEpithet: *thoracica*; scientificNameAuthorship: (Winnertz); **Location:** country: Estonia; county: Mulgi; locality: Muti NR; decimalLatitude: 58.1403; decimalLongitude: 25.6808; **Identification:** identifiedBy: Olavi Kurina; **Event:** samplingProtocol: window trap; eventDate: 2017-06-21 to 2017-07-24; **Record Level:** institutionCode: IZBE; basisOfRecord: Preserved specimen
- **Type status:** Other material. **Occurrence:** catalogNumber: IZBE0251601; recordedBy: Villu Soon; individualCount: 1; sex: male; lifeStage: imago; preparations: specimen in 70% ethanol; associatedOccurrences: BDJ_12308_2; occurrenceID: 343F738B-AF79-5320-809B-64140BE9E933; **Taxon:** scientificName: *Coelophthiniathoracica*; order: Diptera; family: Mycetophilidae; genus: Coelophthinia; specificEpithet: *thoracica*; scientificNameAuthorship: (Winnertz); **Location:** country: Estonia; county: Tartu; locality: Palupőhja, Kaha; decimalLatitude: 58.4318; decimalLongitude: 26.2413; **Identification:** identifiedBy: Olavi Kurina; **Event:** samplingProtocol: Malaise trap; eventDate: 2009-08-4 to 2009-08-18; **Record Level:** institutionCode: IZBE; basisOfRecord: Preserved specimen
- **Type status:** Other material. **Occurrence:** catalogNumber: IZBE0251604; individualCount: 1; sex: female; lifeStage: imago; associatedReferences: Kurina, O.; Grootaert, P. (2016). Fungus gnats in the Botanical garden Jean Massart on the outskirts of Brussels: 52 new country records and a pictorial atlas of the genera (Diptera: Sciaroidea). Belgian Journal of Entomology, 44, 1−44.; associatedOccurrences: BDJ_12308_5; occurrenceID: 8AF32458-97C9-5BF6-B724-CFF22AB81695; **Taxon:** scientificName: *Coelophthiniathoracica*; order: Diptera; family: Mycetophilidae; genus: Coelophthinia; specificEpithet: *thoracica*; scientificNameAuthorship: (Winnertz); **Location:** country: Belgium; stateProvince: Brussels; county: Auderghem; locality: Jardin botanique Jean Massart; decimalLatitude: 50.814; decimalLongitude: 4.4394; **Identification:** identifiedBy: Olavi Kurina; **Event:** samplingProtocol: Malaise trap; eventDate: 2015-10-20 to 2015-10-30; **Record Level:** institutionCode: IZBE; basisOfRecord: Preserved specimen
- **Type status:** Other material. **Occurrence:** catalogNumber: TSZD-JKJ-106938; recordedBy: O. Kurina; individualCount: 1; sex: male; lifeStage: imago; preparations: Legs consumed for barcoding; associatedReferences: Kurina, O.; Grootaert, P. (2016). Fungus gnats in the Botanical garden Jean Massart on the outskirts of Brussels: 52 new country records and a pictorial atlas of the genera (Diptera: Sciaroidea). Belgian Journal of Entomology, 44, 1−44.; associatedOccurrences: urn:uuid:fc1dd876-6672-4877-9419-f0988733b8d0; occurrenceID: 8AC189EE-4269-536C-87A6-4580ABB3221E; **Taxon:** scientificName: *Coelophthiniathoracica*; order: Diptera; family: Mycetophilidae; genus: Coelophthinia; specificEpithet: *thoracica*; scientificNameAuthorship: (Winnertz); **Location:** country: Belgium; stateProvince: Bruxelles; municipality: Suderghem; locality: Jardin Massart; decimalLatitude: 50.8134; decimalLongitude: 4.43645; coordinateUncertaintyInMeters: 250; **Identification:** identifiedBy: J. Kjærandsen; dateIdentified: Mar-28-2019; **Event:** samplingProtocol: Malaise trap; eventDate: 2015-10-20 to 2015-10-30; **Record Level:** institutionCode: ICBE - BOLD voucher; collectionCode: TMU-JKJ-COL-000838; basisOfRecord: Preserved specimen
- **Type status:** Other material. **Occurrence:** catalogNumber: IZBE025160; recordedBy: Olavi Kurina; individualCount: 1; sex: male; lifeStage: imago; preparations: slide mounted in Euparal; associatedReferences: Ševčík, J.; Kurina, O. (2011). Fungus gnats (Diptera: Sciaroidea) of the Gemer region (Central Slovakia): Part 2 - Mycetophilidae. Casopis Slezského Zemského Muzea (A), 60, 97−126. DOI: 10.2478/v10210-011-0011-x.; associatedOccurrences: BDJ_12308_4; occurrenceID: 9F856CF2-F149-5BFC-B8F3-9B0909AAB999; **Taxon:** scientificName: *Coelophthiniathoracica*; order: Diptera; family: Mycetophilidae; genus: Coelophthinia; specificEpithet: *thoracica*; scientificNameAuthorship: (Winnertz); **Location:** country: Slovakia; county: Banská Bystrica Region; municipality: Revúca District; locality: NP Muránska planina, Maretkiná; verbatimElevation: 1002 m; decimalLatitude: 48.7705; decimalLongitude: 20.0287; **Identification:** identifiedBy: Olavi Kurina; **Event:** samplingProtocol: sweep net; eventDate: 05/26/2009; **Record Level:** institutionCode: IZBE; basisOfRecord: Preserved specimen
- **Type status:** Other material. **Occurrence:** catalogNumber: IZBE0251602; recordedBy: C. Lange & J. Ziegler; individualCount: 1; sex: male; lifeStage: imago; preparations: pinned specimen (mounted from ethanol); associatedReferences: Kurina, O. 2008. Sciaroidea excl. Sciaridae. In Ziegler, J. (ed.) Diptera Stelviana. A dipterological perspective on a changing alpine landscape. Volume 1. Studia Dipterologica. Supplements, 16, 245−293.; associatedOccurrences: BDJ_12308_3; occurrenceID: 3BC0A359-04E8-5D03-A666-E28477F1DFFD; **Taxon:** scientificName: *Coelophthiniathoracica*; order: Diptera; family: Mycetophilidae; genus: Coelophthinia; specificEpithet: *thoracica*; scientificNameAuthorship: (Winnertz); **Location:** country: Italy; stateProvince: Trentino-South Tyrol; county: South Tyrol; locality: N. Park Stilfser Joch, Suldental (O von Gomagoi); verbatimElevation: 1220 m; decimalLatitude: 46.576; decimalLongitude: 10.5475; **Identification:** identifiedBy: Olavi Kurina; **Event:** samplingProtocol: Malaise trap; eventDate: 2005-09-05 to 2005-09-19; **Record Level:** institutionCode: IZBE; basisOfRecord: Preserved specimen

#### Description

Male (Figs 1, 3, 5).

Coloration and most body characteristics as in the genus description. Body length 3.6–4.2 mm. Wing length 2.8–3.1 mm; ratio of length to width 3.1–3.3. Sensory organ dorsally on the basal half of mid-tibia elongate oval, 6.3–8.1 times longer than wide, length 0.26–0.29 that of tibial length.

Terminalia. Tergite 9 rectangular in dorsal view, about 1.3 times as long as wide, medially somewhat constricted, curved like a hood in lateral view. Medial protrusion of tergite 10 short and rounded, about as high as wide in posterior view, densely setose. Gonocoxites in lateral view with a somewhat elongated outline, with straight or slightly convex ventral margin. Posterolateral lobe of gonocoxite long and narrow, constricted section about 3x longer than wide, rounded apically, laterally bare for about 3x apical width. Spathulate gonocoxal lobe 7–7.5 times as long as wide, curved slightly medially, with constricted base, basad of base with a group of 3–5 longer setae. Dorsal branch of gonostylus small, simple, semicircular, dorsally setose with one extra long seta deviating from others. The broad lobe 1 (cf. Fig. 5d) fan-shaped, carrying 7–8 blunt setae along rim and 5 normal very long setae subapically on inner side. The narrow, acute tipped lobe 2 (cf. Fig. 5c, d) medially somewhat expanding, with strong, blunt seta placed nearer to the tip than its length; followed by one short and one very long seta in mid-section. Aedeagus 1.2x longer than spathulate gonocoxal lobe in lateral view, with broadest medial section, somewhat narrowed basal section and apically sharply narrowed into downcurved hook; apically with a short spike at the outer side at the curving point; downcurved hook not longer than width of medial section of aedeagus.

Female (Fig. 4).

Coloration as for male. Sensory organ dorsally on the basal half of mid-tibia elongate oval, 7.4 times longer than wide, length 0.28 that of tibial length.

Terminalia as described for genus. Tergite 8 short, posteriorly emarginated in dorsal view, bare, except 1–2 small setae posterolaterally. Tergite 9 short, posteriorly emarginated in dorsal view, bare, except apical margin with row of 7–8 long setae, which are longer than those on cercus. Cercus about two times as long as wide medially, evenly covered with setae. Sternite 8 longer than tergites 8 and 9 together, posterolaterally rounded, posterior half setose, in ventral view hypogynal valves separated by V-shaped deep incision, about one-third as deep as segment length. Gonapophysis 8 elongate, about 6 times as long as wide, extending beyond mid-cercus, ventrally setose, with one deviating strong subapical seta.

#### Diagnosis

*Coelophthiniathoracica* can be distinguished from other species of the genus by the combination of having a somewhat elongated shape of the gonocoxite as seen in lateral view, with a straight or slightly convex ventral margin, a long and slender posterolateral lobe and a short and rounded medial protrusion of tergite 10. The aedeagus is uniquely shaped, long, with ventrally curved hook not longer than width of medial section of aedeagus. The length of the mid-tibial organ is about 0.3 of tibial length.

### Coelophthinia lata

Kjaerandsen sp. nov.

C8E20621-2754-5471-AD44-3E743C8C93BD

http://www.boldsystems.org/index.php/Public_BarcodeCluster?clusteruri=BOLD:ACZ6758

78CB434E-611B-434E-A51C-56070B74C9E2

#### Materials

- **Type status:** Holotype. **Occurrence:** catalogNumber: TSZD-JKJ-105617; recordedBy: J. Kjærandsen, J. P. Lindemann & P. Dominiak; individualCount: 1; sex: male; lifeStage: adult; preparations: Pinned (HMDS-dried from ethanol); associatedOccurrences: urn:uuid:a6b455a7-12ad-422d-b4cd-fced8d1e9e98; occurrenceID: 1AF4B55D-2594-5560-8335-43201D913260; **Taxon:** scientificName: *Coelophthinialata*; order: Diptera; family: Mycetophilidae; genus: Coelophthinia; specificEpithet: *lata*; scientificNameAuthorship: Kjaerandsen; **Location:** country: Norway; stateProvince: Nordland (NSI); municipality: Grane; locality: Auster-Vefsna NR, Stilleelva W; decimalLatitude: 65.53278; decimalLongitude: 13.72667; coordinateUncertaintyInMeters: 10; **Identification:** identifiedBy: J. Kjærandsen; dateIdentified: Aug-06-2020; **Event:** samplingProtocol: window trap; eventDate: 2018-05-28 to 2018-07-30; eventRemarks: WT 1; **Record Level:** institutionCode: TSZ - BOLD voucher; collectionCode: TMU-JKJ-COL-000628
- **Type status:** Paratype. **Occurrence:** catalogNumber: TSZD-JKJ-101623; recordedBy: J. Kjærandsen & M. T. Dahl; individualCount: 1; sex: male; lifeStage: adult; preparations: Pinned (HMDS-dried from ethanol); associatedOccurrences: urn:uuid:67eaed1c-e27e-4527-8c39-55233258f134; occurrenceID: 736FC47B-EC12-5F3E-8ABC-835BF0D840A6; **Taxon:** scientificName: *Coelophthinialata*; order: Diptera; family: Mycetophilidae; genus: Coelophthinia; specificEpithet: *lata*; scientificNameAuthorship: Kjaerandsen; **Location:** country: Norway; stateProvince: Troms (TRI); municipality: Målselv; locality: Skaktardalen N, Øvre Dividal LVN, WT-1; decimalLatitude: 68.76306; decimalLongitude: 19.72417; coordinateUncertaintyInMeters: 10; **Identification:** identifiedBy: J. Kjærandsen; dateIdentified: Aug-06-2020; **Event:** samplingProtocol: Triangle window trap w/camo-roof; eventDate: 2015-08-24 to 2015-09-15; eventRemarks: WT-2; **Record Level:** institutionCode: TSZ - BOLD voucher; collectionCode: TMU-JKJ-COL-000298
- **Type status:** Paratype. **Occurrence:** catalogNumber: TSZD-JKJ-101624; recordedBy: J. Kjæandsen & M. T. Dahl; individualCount: 1; sex: male; lifeStage: adult; preparations: Pinned (HMDS-dried from ethanol); associatedOccurrences: urn:uuid:6e690999-3826-46ba-b1c9-0420a0319827; occurrenceID: 07B88691-4500-5BC1-A783-92E4ED3FE92B; **Taxon:** scientificName: *Coelophthinialata*; order: Diptera; family: Mycetophilidae; genus: Coelophthinia; specificEpithet: *lata*; scientificNameAuthorship: Kjaerandsen; **Location:** country: Norway; stateProvince: Troms (TRI); municipality: Målselv; locality: Skaktardalen N, Øvre Dividal LVN, WT-2; decimalLatitude: 68.76306; decimalLongitude: 19.72417; coordinateUncertaintyInMeters: 10; **Identification:** identifiedBy: J. Kjærandsen; dateIdentified: Aug-06-2020; **Event:** samplingProtocol: Triangle window trap w/camo-roof; eventDate: 2015-08-24 to 2015-09-15; eventRemarks: WT-2; **Record Level:** institutionCode: TSZ - BOLD voucher; collectionCode: TMU-JKJ-COL-000298
- **Type status:** Paratype. **Occurrence:** catalogNumber: TSZD-JKJ-102314; recordedBy: J. Kjærandsen; individualCount: 1; sex: male; lifeStage: adult; preparations: Pinned (HMDS-dried from ethanol); associatedOccurrences: urn:uuid:14f7ff65-17a4-4976-b2fc-3234ef57b681; occurrenceID: 9BF840DC-5A38-5496-B1E7-190E97CA973B; **Taxon:** scientificName: *Coelophthinialata*; order: Diptera; family: Mycetophilidae; genus: Coelophthinia; specificEpithet: *lata*; scientificNameAuthorship: Kjaerandsen; **Location:** country: Norway; stateProvince: Troms (TRI); municipality: Nordreisa; locality: Imofossen and Imoroavvi, Reisa NP; decimalLatitude: 69.29889; decimalLongitude: 22.00389; coordinateUncertaintyInMeters: 250; **Identification:** identifiedBy: J. Kjærandsen; dateIdentified: Aug-06-2020; **Event:** samplingProtocol: sweep net; eventDate: 07/20/2016; eventRemarks: around and N Imofossen; **Record Level:** institutionCode: TSZ - BOLD voucher; collectionCode: TMU-JKJ-COL-000395
- **Type status:** Paratype. **Occurrence:** catalogNumber: TSZD-JKJ-110150; recordedBy: J. Kjærandsen & M. T. Dahl; individualCount: 1; sex: male; lifeStage: adult; preparations: Pinned (HMDS-dried from ethanol); associatedOccurrences: urn:uuid:aa7b5ed0-1427-458a-88a3-dade239d363a; occurrenceID: DD4E7B98-D023-54D5-B1B2-744C995C0C7B; **Taxon:** scientificName: *Coelophthinialata*; order: Diptera; family: Mycetophilidae; genus: Coelophthinia; specificEpithet: *lata*; scientificNameAuthorship: Kjaerandsen; **Location:** country: Norway; stateProvince: Troms (TRI); municipality: Målselv; locality: Skaktardalen N, Øvre Dividal LVN, MT-2; decimalLatitude: 68.76389; decimalLongitude: 19.72361; coordinateUncertaintyInMeters: 50; **Identification:** identifiedBy: J. Kjærandsen; dateIdentified: Apr-05-2020; **Event:** samplingProtocol: Malaise trap; eventDate: 2015-08-24 to 2015-09-15; eventRemarks: MT-2-DOWN; **Record Level:** institutionCode: TSZ; collectionCode: TMU-JKJ-COL-000300
- **Type status:** Paratype. **Occurrence:** catalogNumber: TSZD-JKJ-104763; recordedBy: J. Kjærandsen, J. P. Lindemann & P. Dominiak; individualCount: 1; sex: male; lifeStage: adult; preparations: Pinned (HMDS-dried from ethanol); associatedOccurrences: urn:uuid:90e15539-0d53-4b84-a45f-87848d925fdc; occurrenceID: 1F57ACB8-1488-53D2-940B-A389D43D40D8; **Taxon:** scientificName: *Coelophthinialata*; order: Diptera; family: Mycetophilidae; genus: Coelophthinia; specificEpithet: *lata*; scientificNameAuthorship: Kjaerandsen; **Location:** country: Norway; stateProvince: Nordland (NSI); municipality: Grane; locality: Auster-Vefsna NR, Stilleelva W; decimalLatitude: 65.53194; decimalLongitude: 13.725; coordinateUncertaintyInMeters: 10; **Identification:** identifiedBy: J. Kjærandsen; dateIdentified: Aug-06-2020; **Event:** samplingProtocol: window trap; eventDate: 2018-05-28 to 2018-07-30; eventRemarks: WT 2; **Record Level:** institutionCode: TSZ - BOLD voucher; collectionCode: TMU-JKJ-COL-000631
- **Type status:** Paratype. **Occurrence:** catalogNumber: TSZD-JKJ-105025; recordedBy: J. Kjærandsen, J. P. Lindemann & P. Dominiak; individualCount: 1; sex: male; lifeStage: adult; preparations: Pinned (HMDS-dried from ethanol); associatedOccurrences: urn:uuid:2de1b742-dc94-4067-8612-8c162cf25c67; occurrenceID: 2BDAADE0-D894-594C-9BA3-0BBFF7794513; **Taxon:** scientificName: *Coelophthinialata*; order: Diptera; family: Mycetophilidae; genus: Coelophthinia; specificEpithet: *lata*; scientificNameAuthorship: Kjaerandsen; **Location:** country: Norway; stateProvince: Nordland (NSI); municipality: Grane; locality: Auster-Vefsna NR, Stilleelva W; decimalLatitude: 65.53278; decimalLongitude: 13.72667; coordinateUncertaintyInMeters: 10; **Identification:** identifiedBy: J. Kjærandsen; dateIdentified: Aug-06-2020; **Event:** samplingProtocol: window trap; eventDate: 2018-07-30 to 2018-10-05; eventRemarks: WT 1; **Record Level:** institutionCode: TSZ - BOLD voucher; collectionCode: TMU-JKJ-COL-000793
- **Type status:** Paratype. **Occurrence:** catalogNumber: TSZD-JKJ-107563; recordedBy: J. Kjærandsen, J. P. Lindemann & P. Dominiak; individualCount: 1; sex: male; lifeStage: adult; preparations: Pinned (HMDS-dried from ethanol); associatedOccurrences: urn:uuid:33e45671-f7a2-468b-a226-976191619090; occurrenceID: A18C2DC3-05FC-580E-9882-2B4C1EDA4920; **Taxon:** scientificName: *Coelophthinialata*; order: Diptera; family: Mycetophilidae; genus: Coelophthinia; specificEpithet: *lata*; scientificNameAuthorship: Kjaerandsen; **Location:** country: Norway; stateProvince: Nordland (NSI); municipality: Saltdal; locality: Rognan, Fiskvågmo; minimumElevationInMeters: 35; decimalLatitude: 67.0925; decimalLongitude: 15.36083; coordinateUncertaintyInMeters: 10; **Identification:** identifiedBy: J. Kjærandsen; dateIdentified: Aug-06-2020; **Event:** samplingProtocol: window trap; eventDate: 2019-05-28 to 2019-07-22; eventRemarks: WT 2 - 2019 - 1; **Record Level:** institutionCode: TSZ - BOLD voucher; collectionCode: TMU-JKJ-COL-000933
- **Type status:** Paratype. **Occurrence:** catalogNumber: TSZD-JKJ-112682; recordedBy: M. Jaschhof; individualCount: 1; sex: male; lifeStage: adult; preparations: Pinned (HMDS-dried from ethanol); occurrenceID: 2704BD26-6D7C-5F3E-9511-9E02EE192B48; **Taxon:** scientificName: *Coelophthinialata*; order: Diptera; family: Mycetophilidae; genus: Coelophthinia; specificEpithet: *lata*; scientificNameAuthorship: Kjaerandsen; **Location:** country: Sweden; stateProvince: Uppsala (UP); municipality: Östhammar; locality: Andersby NR SW Österbybruk; verbatimCoordinates: 60.09N, 17.50E; decimalLatitude: 60.10; decimalLongitude: 17.6; **Identification:** identifiedBy: J. Kjærandsen; dateIdentified: 03/22/2022; **Event:** eventDate: 09/10/2005; **Record Level:** institutionCode: TSZ; collectionCode: COL-003196
- **Type status:** Paratype. **Occurrence:** catalogNumber: TSZD-JKJ-207664; recordedBy: K. Müller; individualCount: 1; sex: male; lifeStage: adult; preparations: Slide in Canada Balsam; associatedOccurrences: urn:uuid:c1332da6-89a8-486a-aea6-e0b77faf223c; occurrenceID: B0A30B83-77C5-5E7E-A33B-7A4835A62254; **Taxon:** scientificName: *Coelophthinialata*; order: Diptera; family: Mycetophilidae; genus: Coelophthinia; specificEpithet: *lata*; scientificNameAuthorship: Kjaerandsen; **Location:** country: Sweden; stateProvince: Norrbottens län (LU); municipality: Jokkmokk; locality: Messaure; minimumElevationInMeters: 175; decimalLatitude: 66.68262; decimalLongitude: 20.36322; coordinateUncertaintyInMeters: 1000; **Identification:** identifiedBy: J. Kjærandsen; dateIdentified: May-31-2021; **Event:** samplingProtocol: Barber traps; eventDate: 1971-09-02 to 1971-10-04; **Record Level:** institutionCode: TSZ; collectionCode: COL-001968
- **Type status:** Paratype. **Occurrence:** catalogNumber: TSZD-JKJ-111325; recordedBy: M. & C. Jaschhof; individualCount: 1; sex: male; lifeStage: adult; preparations: 80% alc.; associatedOccurrences: urn:uuid:83e15cc1-97e5-4d07-990b-069faea0300b; occurrenceID: D06677DF-D110-5D35-82DA-26C521934915; **Taxon:** scientificName: *Coelophthinialata*; order: Diptera; family: Mycetophilidae; genus: Coelophthinia; specificEpithet: *lata*; scientificNameAuthorship: Kjaerandsen; **Location:** country: Finland; stateProvince: South Karelia; municipality: Parikkala; locality: Lake Siikalahti, W Kaukola; decimalLatitude: 61.55917; decimalLongitude: 29.57056; coordinateUncertaintyInMeters: 250; **Identification:** identifiedBy: J. Kjærandsen; dateIdentified: Aug-12-2020; **Event:** eventDate: 2004-06-24 to 2004-08-19; **Record Level:** institutionCode: IZBE - donation to O. Kurina 12/8-20; collectionCode: COL-008021
- **Type status:** Paratype. **Occurrence:** catalogNumber: TSZD-JKJ-111326; recordedBy: M. & C. Jaschhof; individualCount: 1; sex: male; lifeStage: adult; preparations: 80% alc.; associatedOccurrences: urn:uuid:aea3b040-40bb-4f3e-babe-ed5a5617afae; occurrenceID: E1C19AC8-FE4F-55D1-9999-66207D256187; **Taxon:** scientificName: *Coelophthinialata*; order: Diptera; family: Mycetophilidae; genus: Coelophthinia; specificEpithet: *lata*; scientificNameAuthorship: Kjaerandsen; **Location:** country: Finland; stateProvince: South Karelia; municipality: Parikkala; locality: Lake Siikalahti, W Kaukola; decimalLatitude: 61.55917; decimalLongitude: 29.57056; coordinateUncertaintyInMeters: 250; **Identification:** identifiedBy: J. Kjærandsen; dateIdentified: Aug-06-2020; **Event:** eventDate: 2004-06-24 to 2004-08-19; **Record Level:** institutionCode: IZBE - donation to O. Kurina 12/8-20; collectionCode: COL-008021
- **Type status:** Paratype. **Occurrence:** catalogNumber: TSZD-JKJ-111327; recordedBy: M. & C. Jaschhof; individualCount: 1; sex: male; lifeStage: adult; preparations: Pinned (HMDS-dried from ethanol); associatedOccurrences: urn:uuid:1c270c2d-9ac7-48bb-bb72-981a34671761; occurrenceID: 25C13508-0390-5375-84A5-1CB3CF59CE9B; **Taxon:** scientificName: *Coelophthinialata*; order: Diptera; family: Mycetophilidae; genus: Coelophthinia; specificEpithet: *lata*; scientificNameAuthorship: Kjaerandsen; **Location:** country: Finland; stateProvince: South Karelia; municipality: Parikkala; locality: Lake Siikalahti, W Kaukola; decimalLatitude: 61.55917; decimalLongitude: 29.57056; coordinateUncertaintyInMeters: 250; **Identification:** identifiedBy: J. Kjærandsen; dateIdentified: Aug-06-2020; **Event:** eventDate: 2004-06-24 to 2004-08-19; **Record Level:** institutionCode: TSZ; collectionCode: COL-008021
- **Type status:** Other material. **Occurrence:** catalogNumber: TSZD-JKJ-102400; recordedBy: J. Kjærandsen; individualCount: 1; sex: male; lifeStage: adult; preparations: Pinned (HMDS-dried from ethanol); associatedOccurrences: urn:uuid:a1eef325-00ed-4687-93c3-0c4c873c9064; occurrenceID: 03A469E8-757D-5135-B0C8-7A36DCCA388F; **Taxon:** scientificName: *Coelophthinialata*; order: Diptera; family: Mycetophilidae; genus: Coelophthinia; specificEpithet: *lata*; scientificNameAuthorship: Kjaerandsen; **Location:** country: Norway; stateProvince: Troms (TRI); municipality: Nordreisa; locality: Swamp forest S Lorrioholmen, Reisa NP W Naustneset; minimumElevationInMeters: 130; decimalLatitude: 69.3362; decimalLongitude: 21.9394; coordinateUncertaintyInMeters: 10; **Identification:** identifiedBy: J. Kjærandsen; dateIdentified: Aug-06-2020; **Event:** samplingProtocol: Triangle window trap w/camo-roof; eventDate: 2016-07-19 to 2016-09-20; eventRemarks: WT-5, flomskog; **Record Level:** institutionCode: TSZ - BOLD voucher; collectionCode: TMU-JKJ-COL-000392
- **Type status:** Other material. **Occurrence:** catalogNumber: TSZD-JKJ-102401; recordedBy: J. Kjærandsen; individualCount: 1; sex: male; lifeStage: adult; preparations: Pinned (HMDS-dried from ethanol); associatedOccurrences: urn:uuid:5d83ad61-b801-4050-a82c-fc9d98c544c5; occurrenceID: CA97F5EE-06A2-56EA-9BEC-EC2665E9B46D; **Taxon:** scientificName: *Coelophthinialata*; order: Diptera; family: Mycetophilidae; genus: Coelophthinia; specificEpithet: *lata*; scientificNameAuthorship: Kjaerandsen; **Location:** country: Norway; stateProvince: Troms (TRI); municipality: Nordreisa; locality: Swamp forest S Lorrioholmen, Reisa NP W Naustneset; minimumElevationInMeters: 130; decimalLatitude: 69.3362; decimalLongitude: 21.9394; coordinateUncertaintyInMeters: 10; **Identification:** identifiedBy: J. Kjærandsen; dateIdentified: Aug-06-2020; **Event:** samplingProtocol: Triangle window trap w/camo-roof; eventDate: 2016-07-19 to 2016-09-20; eventRemarks: WT-5, flomskog; **Record Level:** institutionCode: TSZ - BOLD voucher; collectionCode: TMU-JKJ-COL-000392
- **Type status:** Other material. **Occurrence:** catalogNumber: TSZD-JKJ-215016; recordedBy: M. Jaschhof; individualCount: 1; sex: male; lifeStage: adult; preparations: 80% alc.; associatedOccurrences: urn:uuid:b03cfe6f-0001-428b-89e7-8ba096e5965d; occurrenceID: 40541669-1B4C-5DD3-B336-7C66A312F53D; **Taxon:** scientificName: *Coelophthinialata*; order: Diptera; family: Mycetophilidae; genus: Coelophthinia; specificEpithet: *lata*; scientificNameAuthorship: Kjaerandsen; **Location:** country: Sweden; stateProvince: Uppsala län (UP); municipality: Uppsala; locality: Fiby NR; decimalLatitude: 59.53; decimalLongitude: 17.21; coordinateUncertaintyInMeters: 1000; **Identification:** identifiedBy: J. Kjærandsen; dateIdentified: Jun-21-2021; **Event:** samplingProtocol: sweep net & aspirator; eventDate: 09/11/2005; **Record Level:** institutionCode: TSZ [transferred from MZLU 2014]; collectionCode: COL-003197
- **Type status:** Other material. **Occurrence:** catalogNumber: TSZD-JKJ-261215; recordedBy: K. Müller; individualCount: 1; sex: male; lifeStage: adult; preparations: Ethanol (80%); associatedOccurrences: urn:uuid:55cde649-afef-4920-ba6d-e004ef7394d2; occurrenceID: 6F8229DF-CC2F-531B-A9D1-9A5010D8E0DE; **Taxon:** scientificName: *Coelophthinialata*; order: Diptera; family: Mycetophilidae; genus: Coelophthinia; specificEpithet: *lata*; scientificNameAuthorship: Kjaerandsen; **Location:** country: Sweden; stateProvince: Norrbottens län (TO); municipality: Kiruna; locality: Abisko; decimalLatitude: 68.35027; decimalLongitude: 18.83047; coordinateUncertaintyInMeters: 1000; **Identification:** identifiedBy: J. Kjærandsen; dateIdentified: Jun-21-2021; **Event:** samplingProtocol: light trap; eventDate: 1976-07-19 to 1976-07-26; eventRemarks: LF-05, 150-500 m W Naturv. stn.; **Record Level:** institutionCode: TSZ; collectionCode: COL-008061
- **Type status:** Other material. **Occurrence:** catalogNumber: TSZD-JKJ-112869; recordedBy: M. Jaschhof; individualCount: 1; sex: male; lifeStage: adult; preparations: Pinned (HMDS-dried from ethanol); occurrenceID: 80910862-4CD2-5F09-80BE-BE874E54D7DC; **Taxon:** scientificName: *Coelophthinialata*; order: Diptera; family: Mycetophilidae; genus: Coelophthinia; specificEpithet: *lata*; scientificNameAuthorship: Kjaerandsen; **Location:** country: Sweden; stateProvince: Uppsala (UP); municipality: Östhammar; locality: Andersby NR SW Österbybruk; verbatimCoordinates: 60.09N, 17.50E; decimalLatitude: 60.10; decimalLongitude: 17.6; **Identification:** identifiedBy: J. Kjærandsen; dateIdentified: 03/22/2022; **Event:** eventDate: 09/10/2005; **Record Level:** institutionCode: TSZ; collectionCode: COL-003196
- **Type status:** Other material. **Occurrence:** catalogNumber: TSZD-JKJ-236791; recordedBy: M. & C. Jaschhof; individualCount: 1; sex: male; lifeStage: adult; preparations: 80% alc.; associatedOccurrences: urn:uuid:b80308eb-2b8e-4014-923c-cb1b4c4b45a6; occurrenceID: 8917B6FA-4346-5D0E-801D-375C711F223A; **Taxon:** scientificName: *Coelophthinialata*; order: Diptera; family: Mycetophilidae; genus: Coelophthinia; specificEpithet: *lata*; scientificNameAuthorship: Kjaerandsen; **Location:** country: Finland; stateProvince: South Karelia; municipality: Parikkala; locality: Lake Siikalahti, W Kaukola; decimalLatitude: 61.55917; decimalLongitude: 29.57056; coordinateUncertaintyInMeters: 250; **Identification:** identifiedBy: J. Kjærandsen; dateIdentified: Aug-06-2020; **Event:** eventDate: 2004-06-24 to 2004-08-19; **Record Level:** institutionCode: MZLU; collectionCode: COL-008021
- **Type status:** Other material. **Occurrence:** catalogNumber: TSZD-JKJ-111328; recordedBy: M. & C. Jaschhof; individualCount: 1; sex: male; lifeStage: adult; preparations: Pinned (HMDS-dried from ethanol); associatedOccurrences: urn:uuid:ac337cda-18f3-44e8-98ca-02a0400ea17e; occurrenceID: 934E1C33-5AE1-5957-BFB7-0AACFA911086; **Taxon:** scientificName: *Coelophthinialata*; order: Diptera; family: Mycetophilidae; genus: Coelophthinia; specificEpithet: *lata*; scientificNameAuthorship: Kjaerandsen; **Location:** country: Finland; stateProvince: South Karelia; municipality: Parikkala; locality: Lake Siikalahti, W Kaukola; decimalLatitude: 61.55917; decimalLongitude: 29.57056; coordinateUncertaintyInMeters: 250; **Identification:** identifiedBy: J. Kjærandsen; dateIdentified: Aug-06-2020; **Event:** eventDate: 2004-06-24 to 2004-08-19; **Record Level:** institutionCode: TSZ; collectionCode: COL-008021
- **Type status:** Other material. **Occurrence:** catalogNumber: TSZD-JKJ-111329; recordedBy: M. & C. Jaschhof; individualCount: 1; sex: male; lifeStage: adult; preparations: Pinned (HMDS-dried from ethanol); associatedOccurrences: urn:uuid:07658590-4ff7-41f6-a9cc-5224f55f8ae3; occurrenceID: 42EDF7EB-939D-5F7C-8B69-D21700DD80DE; **Taxon:** scientificName: *Coelophthinialata*; order: Diptera; family: Mycetophilidae; genus: Coelophthinia; specificEpithet: *lata*; scientificNameAuthorship: Kjaerandsen; **Location:** country: Finland; stateProvince: South Karelia; municipality: Parikkala; locality: Lake Siikalahti, W Kaukola; decimalLatitude: 61.55917; decimalLongitude: 29.57056; coordinateUncertaintyInMeters: 250; **Identification:** identifiedBy: J. Kjærandsen; dateIdentified: Aug-06-2020; **Event:** eventDate: 2004-06-24 to 2004-08-19; **Record Level:** institutionCode: TSZ; collectionCode: COL-008021
- **Type status:** Other material. **Occurrence:** catalogNumber: TSZD-JKJ-111330; recordedBy: M. & C. Jaschhof; individualCount: 1; sex: male; lifeStage: adult; preparations: Pinned (HMDS-dried from ethanol); associatedOccurrences: urn:uuid:1ac60672-b049-4d6f-99b6-e284a0b4053d; occurrenceID: C8094261-52AA-59A0-B612-2FAEC7E4191C; **Taxon:** scientificName: *Coelophthinialata*; order: Diptera; family: Mycetophilidae; genus: Coelophthinia; specificEpithet: *lata*; scientificNameAuthorship: Kjaerandsen; **Location:** country: Finland; stateProvince: South Karelia; municipality: Parikkala; locality: Lake Siikalahti, W Kaukola; decimalLatitude: 61.55917; decimalLongitude: 29.57056; coordinateUncertaintyInMeters: 250; **Identification:** identifiedBy: J. Kjærandsen; dateIdentified: Aug-06-2020; **Event:** eventDate: 2004-06-24 to 2004-08-19; **Record Level:** institutionCode: TSZ; collectionCode: COL-008021
- **Type status:** Other material. **Occurrence:** catalogNumber: TSZD-JKJ-111331; recordedBy: M. & C. Jaschhof; individualCount: 1; sex: male; lifeStage: adult; preparations: Pinned (HMDS-dried from ethanol); associatedOccurrences: urn:uuid:8e6d498c-79d2-4f0a-ac55-add2cd03027f; occurrenceID: 3A33BEAF-4277-583A-9A1C-7D1F79D21346; **Taxon:** scientificName: *Coelophthinialata*; order: Diptera; family: Mycetophilidae; genus: Coelophthinia; specificEpithet: *lata*; scientificNameAuthorship: Kjaerandsen; **Location:** country: Finland; stateProvince: South Karelia; municipality: Parikkala; locality: Lake Siikalahti, W Kaukola; decimalLatitude: 61.55917; decimalLongitude: 29.57056; coordinateUncertaintyInMeters: 250; **Identification:** identifiedBy: J. Kjærandsen; dateIdentified: Aug-06-2020; **Event:** eventDate: 2004-06-24 to 2004-08-19; **Record Level:** institutionCode: TSZ; collectionCode: COL-008021
- **Type status:** Other material. **Occurrence:** catalogNumber: TSZD-JKJ-111332; recordedBy: M. & C. Jaschhof; individualCount: 1; sex: male; lifeStage: adult; preparations: Pinned (HMDS-dried from ethanol); associatedOccurrences: urn:uuid:8331c262-c7fb-4fba-8f69-3463f2139bdc; occurrenceID: FF10C98D-8D83-5B04-8530-D9FA6FFCD257; **Taxon:** scientificName: *Coelophthinialata*; order: Diptera; family: Mycetophilidae; genus: Coelophthinia; specificEpithet: *lata*; scientificNameAuthorship: Kjaerandsen; **Location:** country: Finland; stateProvince: South Karelia; municipality: Parikkala; locality: Lake Siikalahti, W Kaukola; decimalLatitude: 61.55917; decimalLongitude: 29.57056; coordinateUncertaintyInMeters: 250; **Identification:** identifiedBy: J. Kjærandsen; dateIdentified: Aug-06-2020; **Event:** eventDate: 2004-06-24 to 2004-08-19; **Record Level:** institutionCode: TSZ; collectionCode: COL-008021

#### Description

Male (Fig. 6, n = 8 for measurements).

Coloration and general body characteristics as in the genus description. Body length 3.5–4.1 mm. Wing length 2.9–3.1 mm; ratio of length to width 2.6. Sensory organ dorsally on the basal half of mid-tibia elongate oval, 7.5 times longer than wide, length 0.25 that of tibial length.

Terminalia. Tergite 9 rectangular in dorsal view, about 1.5 times as long as wide, curved like a hood in lateral view. Medial protrusion of tergite 10 short and rounded, about as high as wide in posterior view, densely setose. Gonocoxites in lateral view with a rounded outline, with distinctly convex ventral margin. Posterolateral lobe of gonocoxite short and wide, constricted section about 1.5x longer than wide, evenly rounded apically, laterally bare for about 2x apical width. Spathulate gonocoxal lobe about seven times as long as wide, without constricted base, basad of base with a row of 3 long setae. Dorsal branch of gonostylus small, simple, oblong and semicircular, dorsally setose with some extra long setae. The broad lobe 1 (cf. Fig. 3f) fan-shaped, carrying 8 blunt setae along rim and 5 normal setae subapically on inner side. The narrow, acute tipped lobe 2 (cf. Fig. 3f) without tiny stiff seta apically; with strong, blunt seta placed nearer to the tip than its length; followed by one short and one long seta in mid-section. Aedeagus 1.5x longer than spathulate gonocoxal lobe in lateral view, with broadest basal section, somewhat narrowed middle section and apically sharply narrowed into downcurved hook; apically with a short spike at the outer side at the curving point; downcurved hook not longer than width of base of aedeagus.

Female unknown.

#### Diagnosis

*Coelophthinialata* can be distinguished from other species of the genus by the combination of having a rounded shape of gonocoxite in lateral view, with distinctly convex ventral margin, broad and short posteriolateral lobe and short and broad, semicircular medial protrusion of tergite 10. The aedeagus is uniquely shaped with apical hook not longer than the height of the base of aedeagus. The length of the mid-tibial organ is about 1/4 of tibial length.

#### Etymology

The species epithet is from the Latin word *lata*, which means broad, denoting the broad caudal extension from the gonocoxite. This feature is diagnostic for this species.

#### Distribution

Nordic, so far known only from Norway, Sweden and Finland.

### Coelophthinia loraasi

Kjaerandsen sp. nov.

57B6F747-3D1D-50DA-BA4B-0FC439003C82

http://www.boldsystems.org/index.php/Public_BarcodeCluster?clusteruri=BOLD:ADV7953

61C183B0-2E04-4791-AF4E-1F6E6FF1C4CF

#### Materials

- **Type status:** Holotype. **Occurrence:** catalogNumber: TSZD-JKJ-105034; recordedBy: J. Kjærandsen, J. P. Lindemann & P. Dominiak; individualCount: 1; sex: male; lifeStage: imago; preparations: Pinned (HMDS-dried from ethanol); associatedOccurrences: urn:uuid:c6175002-859a-43b7-a7c0-7b1aa7f4af3d; occurrenceID: 1723499B-47BF-5A3E-A7AC-78231ACB76EF; **Taxon:** scientificName: *Coelophthinialoraasi*; order: Diptera; family: Mycetophilidae; genus: Coelophthinia; specificEpithet: *loraasi*; scientificNameAuthorship: Kjaerandsen; **Location:** country: Norway; stateProvince: Nordland (NSI); municipality: Grane; locality: Stormobekken; decimalLatitude: 65.595; decimalLongitude: 13.40333; coordinateUncertaintyInMeters: 10; **Identification:** identifiedBy: J. Kjærandsen; dateIdentified: Aug-06-2020; **Event:** samplingProtocol: window trap; eventDate: 2018-07-31 to 2018-10-05; eventRemarks: WT 3; **Record Level:** institutionCode: TSZ - BOLD voucher; collectionCode: TMU-JKJ-COL-000799; basisOfRecord: Preserved specimen
- **Type status:** Paratype. **Occurrence:** catalogNumber: TSZD-JKJ-105026; recordedBy: J. Kjærandsen, J. P. Lindemann & P. Dominiak; individualCount: 1; sex: male; lifeStage: imago; preparations: Pinned (HMDS-dried from ethanol); associatedOccurrences: urn:uuid:d56bc457-f4d6-4bdf-ac79-202b3786d370; occurrenceID: A87FEA3A-47A9-58C8-A51E-0A710678D892; **Taxon:** scientificName: *Coelophthinialoraasi*; order: Diptera; family: Mycetophilidae; genus: Coelophthinia; specificEpithet: *loraasi*; scientificNameAuthorship: Kjaerandsen; **Location:** country: Norway; stateProvince: Nordland (NSI); municipality: Grane; locality: Holmvassdalen NR, Holmvassdalen 2 (Naturbase); decimalLatitude: 65.32472; decimalLongitude: 13.31806; coordinateUncertaintyInMeters: 10; **Identification:** identifiedBy: J. Kjærandsen; dateIdentified: Aug-06-2020; **Event:** samplingProtocol: Malaise trap; eventDate: 2018-08-01 to 2018-10-04; eventRemarks: MT 5; **Record Level:** institutionCode: TSZ - BOLD voucher; collectionCode: TMU-JKJ-COL-000804; basisOfRecord: Preserved specimen
- **Type status:** Paratype. **Occurrence:** catalogNumber: TSZD-JKJ-105027; recordedBy: J. Kjærandsen, J. P. Lindemann & P. Dominiak; individualCount: 1; sex: female; lifeStage: imago; preparations: Pinned (HMDS-dried from ethanol); associatedOccurrences: urn:uuid:8c498cff-4508-46cb-b671-91e22dd0af51; occurrenceID: B463381C-D17A-588A-AF6E-7559F5E06BB0; **Taxon:** scientificName: *Coelophthinialoraasi*; order: Diptera; family: Mycetophilidae; genus: Coelophthinia; specificEpithet: *loraasi*; scientificNameAuthorship: Kjaerandsen; **Location:** country: Norway; stateProvince: Nordland (NSI); municipality: Grane; locality: Holmvassdalen NR, Holmvassdalen 2 (Naturbase); decimalLatitude: 65.32472; decimalLongitude: 13.31806; coordinateUncertaintyInMeters: 10; **Identification:** identifiedBy: J. Kjærandsen; dateIdentified: Aug-06-2020; **Event:** samplingProtocol: Malaise trap; eventDate: 2018-08-01 to 2018-10-04; eventRemarks: MT 5; **Record Level:** institutionCode: TSZ - BOLD voucher; collectionCode: TMU-JKJ-COL-000804; basisOfRecord: Preserved specimen
- **Type status:** Paratype. **Occurrence:** catalogNumber: TSZD-JKJ-111198; recordedBy: J. Kjærandsen, J. P. Lindemann & P. Dominiak; individualCount: 1; sex: male; lifeStage: imago; preparations: Pinned (HMDS-dried from ethanol); associatedOccurrences: urn:uuid:15cb7618-2e04-4c7d-8607-44fa390ee4d2; occurrenceID: 6A975D2E-68D2-5068-BD8B-60301B0BF776; **Taxon:** scientificName: *Coelophthinialoraasi*; order: Diptera; family: Mycetophilidae; genus: Coelophthinia; specificEpithet: *loraasi*; scientificNameAuthorship: Kjaerandsen; **Location:** country: Norway; stateProvince: Nordland (NSI); municipality: Grane; locality: Holmvassdalen NR, Holmvassdalen 2 (Naturbase); decimalLatitude: 65.32472; decimalLongitude: 13.31806; coordinateUncertaintyInMeters: 10; **Identification:** identifiedBy: J. Kjærandsen; dateIdentified: Aug-04-2020; **Event:** samplingProtocol: Malaise trap; eventDate: 2018-08-01 to 2018-10-04; eventRemarks: MT 5; **Record Level:** institutionCode: TSZ - BOLD voucher; collectionCode: TMU-JKJ-COL-000804; basisOfRecord: Preserved specimen
- **Type status:** Paratype. **Occurrence:** catalogNumber: TSZD-JKJ-111128; recordedBy: J. Kjærandsen, J. P. Lindemann & P. Dominiak; individualCount: 1; sex: male; lifeStage: imago; preparations: Pinned (HMDS-dried from ethanol); associatedOccurrences: urn:uuid:9cc93295-3081-446f-9fe8-86b502cadbe2; occurrenceID: BF68D342-515E-503C-A010-4D220A1E35F0; **Taxon:** scientificName: *Coelophthinialoraasi*; order: Diptera; family: Mycetophilidae; genus: Coelophthinia; specificEpithet: *loraasi*; scientificNameAuthorship: Kjaerandsen; **Location:** country: Norway; stateProvince: Nordland (NSI); municipality: Grane; locality: Stormobekken; decimalLatitude: 65.595; decimalLongitude: 13.40333; coordinateUncertaintyInMeters: 10; **Identification:** identifiedBy: J. Kjærandsen; dateIdentified: Aug-06-2020; **Event:** samplingProtocol: window trap; eventDate: 2018-07-31 to 2018-10-05; eventRemarks: WT 3; **Record Level:** institutionCode: IZBE - donation to O. Kurina 12/8-20; collectionCode: TMU-JKJ-COL-000799; basisOfRecord: Preserved specimen
- **Type status:** Paratype. **Occurrence:** catalogNumber: TSZD-JKJ-112069; recordedBy: J. Kjærandsen, J. P. Lindemann & P. Dominiak; individualCount: 1; sex: male; lifeStage: imago; preparations: Pinned (HMDS-dried from ethanol); associatedOccurrences: urn:uuid:0cbb51fa-6fcb-49d0-aa8e-a3e7a796957c; occurrenceID: 307D80E4-3AB0-5857-9C4E-4DD14C6FCD18; **Taxon:** scientificName: *Coelophthinialoraasi*; order: Diptera; family: Mycetophilidae; genus: Coelophthinia; specificEpithet: *loraasi*; scientificNameAuthorship: Kjaerandsen; **Location:** country: Norway; stateProvince: Nordland (NSI); municipality: Grane; locality: Stormobekken; decimalLatitude: 65.595; decimalLongitude: 13.40333; coordinateUncertaintyInMeters: 10; **Identification:** identifiedBy: J. Kjærandsen; dateIdentified: Aug-06-2020; **Event:** samplingProtocol: window trap; eventDate: 2018-07-31 to 2018-10-05; eventRemarks: WT 3; **Record Level:** institutionCode: TSZ; collectionCode: TMU-JKJ-COL-000799; basisOfRecord: Preserved specimen
- **Type status:** Paratype. **Occurrence:** catalogNumber: TSZD-JKJ-112070; recordedBy: J. Kjæandsen, J. P. Lindemann & P. Dominiak; individualCount: 1; sex: male; lifeStage: imago; preparations: Pinned (HMDS-dried from ethanol); associatedOccurrences: urn:uuid:420f4efa-6c91-440b-9f88-9c339949b23d; occurrenceID: 6835A75B-7FF5-573F-A1F0-CDF2866D39EB; **Taxon:** scientificName: *Coelophthinialoraasi*; order: Diptera; family: Mycetophilidae; genus: Coelophthinia; specificEpithet: *loraasi*; scientificNameAuthorship: Kjaerandsen; **Location:** country: Norway; stateProvince: Nordland (NSI); municipality: Grane; locality: Stormobekken; decimalLatitude: 65.595; decimalLongitude: 13.40333; coordinateUncertaintyInMeters: 10; **Identification:** identifiedBy: J. Kjærandsen; dateIdentified: Aug-06-2020; **Event:** samplingProtocol: window trap; eventDate: 2018-07-31 to 2018-10-05; eventRemarks: WT 3; **Record Level:** institutionCode: TSZ; collectionCode: TMU-JKJ-COL-000799; basisOfRecord: Preserved specimen
- **Type status:** Paratype. **Occurrence:** catalogNumber: TSZD-JKJ-105035; recordedBy: J. Kjærandsen, J. P. Lindemann & P. Dominiak; individualCount: 1; sex: female; lifeStage: imago; preparations: Pinned (HMDS-dried from ethanol); associatedOccurrences: urn:uuid:a278486d-5957-4229-aa36-2cf6e7c18333; occurrenceID: 2F7086CD-E243-5EB3-9A48-BB5445EC9970; **Taxon:** scientificName: *Coelophthinialoraasi*; order: Diptera; family: Mycetophilidae; genus: Coelophthinia; specificEpithet: *loraasi*; scientificNameAuthorship: Kjaerandsen; **Location:** country: Norway; stateProvince: Nordland (NSI); municipality: Grane; locality: Stormobekken; decimalLatitude: 65.595; decimalLongitude: 13.40333; coordinateUncertaintyInMeters: 10; **Identification:** identifiedBy: J. Kjærandsen; dateIdentified: Aug-06-2020; **Event:** samplingProtocol: window trap; eventDate: 2018-07-31 to 2018-10-05; eventRemarks: WT 3; **Record Level:** institutionCode: TSZ - BOLD voucher; collectionCode: TMU-JKJ-COL-000799; basisOfRecord: Preserved specimen
- **Type status:** Paratype. **Occurrence:** catalogNumber: TSZD-JKJ-111132; recordedBy: J. Kjærandsen, J. P. Lindemann & P. Dominiak; individualCount: 1; sex: female; lifeStage: imago; preparations: Pinned (HMDS-dried from ethanol); associatedOccurrences: urn:uuid:89d1ac5c-2b81-4265-859f-691f98337f65; occurrenceID: AD42E95F-6B2C-5FEB-978E-2EBE8A70CC07; **Taxon:** scientificName: *Coelophthinialoraasi*; order: Diptera; family: Mycetophilidae; genus: Coelophthinia; specificEpithet: *loraasi*; scientificNameAuthorship: Kjaerandsen; **Location:** country: Norway; stateProvince: Nordland (NSI); municipality: Grane; locality: Stormobekken; decimalLatitude: 65.595; decimalLongitude: 13.40333; coordinateUncertaintyInMeters: 10; **Identification:** identifiedBy: J. Kjærandsen; dateIdentified: Aug-06-2020; **Event:** samplingProtocol: window trap; eventDate: 2018-07-31 to 2018-10-05; eventRemarks: WT 3; **Record Level:** institutionCode: TSZ; collectionCode: TMU-JKJ-COL-000799; basisOfRecord: Preserved specimen
- **Type status:** Other material. **Occurrence:** catalogNumber: TSZD-JKJ-105417; recordedBy: J. Kjærandsen, J. P. Lindemann & P. Dominiak; individualCount: 1; sex: male; lifeStage: imago; preparations: Genitalia voucher for DNA-skimming; associatedOccurrences: urn:uuid:61856d18-5772-4ae9-8117-0f5a6f1f58cc; occurrenceID: 7A9B04AB-B006-53F6-B4D3-904897784769; **Taxon:** scientificName: *Coelophthinialoraasi*; order: Diptera; family: Mycetophilidae; genus: Coelophthinia; specificEpithet: *loraasi*; scientificNameAuthorship: Kjaerandsen; **Location:** country: Norway; stateProvince: Nordland (NSI); municipality: Grane; locality: Auster-Vefsna NR, Stilleelva W; decimalLatitude: 65.53389; decimalLongitude: 13.72778; coordinateUncertaintyInMeters: 10; **Identification:** identifiedBy: J. Kjærandsen; dateIdentified: Jan-16-2019; **Event:** samplingProtocol: Malaise trap; eventDate: 2018-07-30 to 2018-10-05; eventRemarks: MT 1; **Record Level:** institutionCode: TSZ; collectionCode: TMU-JKJ-COL-000792; basisOfRecord: Preserved specimen
- **Type status:** Other material. **Occurrence:** catalogNumber: TSZD-JKJ-105418; recordedBy: J. Kjærandsen, J. P. Lindemann & P. Dominiak; individualCount: 1; sex: male; lifeStage: imago; preparations: Genitalia voucher for DNA-skimming; associatedOccurrences: urn:uuid:61856d18-5772-4ae9-8117-0f5a6f1f58cc; occurrenceID: F1F70A59-410B-5B5C-A40D-FE4DE8B0FC67; **Taxon:** scientificName: *Coelophthinialoraasi*; order: Diptera; family: Mycetophilidae; genus: Coelophthinia; specificEpithet: *loraasi*; scientificNameAuthorship: Kjaerandsen; **Location:** country: Norway; stateProvince: Nordland (NSI); municipality: Grane; locality: Auster-Vefsna NR, Stilleelva W; decimalLatitude: 65.53389; decimalLongitude: 13.72778; coordinateUncertaintyInMeters: 10; **Identification:** identifiedBy: J. Kjærandsen; dateIdentified: Jan-16-2019; **Event:** samplingProtocol: Malaise trap; eventDate: 2018-07-30 to 2018-10-05; eventRemarks: MT 1; **Record Level:** institutionCode: TSZ; collectionCode: TMU-JKJ-COL-000792; basisOfRecord: Preserved specimen
- **Type status:** Other material. **Occurrence:** catalogNumber: TSZD-JKJ-111133; recordedBy: J. Kjærandsen, J. P. Lindemann & P. Dominiak; individualCount: 1; sex: male; lifeStage: imago; preparations: Pinned (HMDS-dried from ethanol); associatedOccurrences: urn:uuid:d3d6685a-3cec-48ad-ba71-ce43ba9f614e; occurrenceID: 51512FBF-07EA-5E26-855C-AAD28DFF7EA6; **Taxon:** scientificName: *Coelophthinialoraasi*; order: Diptera; family: Mycetophilidae; genus: Coelophthinia; specificEpithet: *Coelophthinia*; scientificNameAuthorship: Kjaerandsen; **Location:** country: Norway; stateProvince: Nordland (NSI); municipality: Grane; locality: Holmvassdalen NR, Holmvassdalen 2 (Naturbase); decimalLatitude: 65.32472; decimalLongitude: 13.31806; coordinateUncertaintyInMeters: 10; **Identification:** identifiedBy: J. Kjærandsen; dateIdentified: Aug-06-2020; **Event:** samplingProtocol: Malaise trap; eventDate: 2018-08-01 to 2018-10-04; eventRemarks: MT 5; **Record Level:** institutionCode: TSZ; collectionCode: TMU-JKJ-COL-000804; basisOfRecord: Preserved specimen
- **Type status:** Other material. **Occurrence:** catalogNumber: TSZD-JKJ-111129; recordedBy: J. Kjærandsen, J. P. Lindemann & P. Dominiak; individualCount: 1; sex: male; lifeStage: imago; preparations: Pinned (HMDS-dried from ethanol); associatedOccurrences: urn:uuid:716c9c00-87c2-4c8a-8008-5a1b9b81a2b1; occurrenceID: 5529AAFC-A874-5B72-95C1-C81204C7A3F1; **Taxon:** scientificName: *Coelophthinialoraasi*; order: Diptera; family: Mycetophilidae; genus: Coelophthinia; specificEpithet: *loraasi*; scientificNameAuthorship: Kjaerandsen; **Location:** country: Norway; stateProvince: Nordland (NSI); municipality: Grane; locality: Stormobekken; decimalLatitude: 65.595; decimalLongitude: 13.40333; coordinateUncertaintyInMeters: 10; **Identification:** identifiedBy: J. Kjærandsen; dateIdentified: Aug-06-2020; **Event:** samplingProtocol: window trap; eventDate: 2018-07-31 to 2018-10-05; eventRemarks: WT 3; **Record Level:** institutionCode: TSZ; collectionCode: TMU-JKJ-COL-000799; basisOfRecord: Preserved specimen
- **Type status:** Other material. **Occurrence:** catalogNumber: TSZD-JKJ-111130; recordedBy: J. Kjærandsen, J. P. Lindemann & P. Dominiak; individualCount: 1; sex: male; lifeStage: imago; preparations: Pinned (HMDS-dried from ethanol); associatedOccurrences: urn:uuid:aa08ab08-222d-4e4a-8d1e-cde0fa500608; occurrenceID: 1E9DDBE0-A572-5131-B77C-29FC1333AD10; **Taxon:** scientificName: *Coelophthinialoraasi*; order: Diptera; family: Mycetophilidae; genus: Coelophthinia; specificEpithet: *loraasi*; scientificNameAuthorship: Kjaerandsen; **Location:** country: Norway; stateProvince: Nordland (NSI); municipality: Grane; locality: Stormobekken; decimalLatitude: 65.595; decimalLongitude: 13.40333; coordinateUncertaintyInMeters: 10; **Identification:** identifiedBy: J. Kjærandsen; dateIdentified: Aug-06-2020; **Event:** samplingProtocol: window trap; eventDate: 2018-07-31 to 2018-10-05; eventRemarks: WT 3; **Record Level:** institutionCode: TSZ; collectionCode: TMU-JKJ-COL-000799; basisOfRecord: Preserved specimen
- **Type status:** Other material. **Occurrence:** catalogNumber: TSZD-JKJ-111131; recordedBy: J. Kjærandsen, J. P. Lindemann & P. Dominiak; individualCount: 1; sex: male; lifeStage: imago; preparations: Pinned (HMDS-dried from ethanol); associatedOccurrences: urn:uuid:3038b308-8675-41dd-985f-fcc8a8e89745; occurrenceID: F8C79E7A-9530-5C37-B194-6F0A701C88BD; **Taxon:** scientificName: *Coelophthinialoraasi*; order: Diptera; family: Mycetophilidae; genus: Coelophthinia; specificEpithet: *loraasi*; scientificNameAuthorship: Kjaerandsen; **Location:** country: Norway; stateProvince: Nordland (NSI); municipality: Grane; locality: Stormobekken; decimalLatitude: 65.595; decimalLongitude: 13.40333; coordinateUncertaintyInMeters: 10; **Identification:** identifiedBy: J. Kjærandsen; dateIdentified: Aug-06-2020; **Event:** samplingProtocol: window trap; eventDate: 2018-07-31 to 2018-10-05; eventRemarks: WT 3; **Record Level:** institutionCode: TSZ; collectionCode: TMU-JKJ-COL-000799; basisOfRecord: Preserved specimen
- **Type status:** Other material. **Occurrence:** catalogNumber: TSZD-JKJ-112071; recordedBy: J. Kjærandsen, J. P. Lindemann & P. Dominiak; individualCount: 1; sex: male; lifeStage: imago; preparations: Pinned (HMDS-dried from ethanol); associatedOccurrences: urn:uuid:01ab91c1-b842-435b-8b45-82a0d6768bcb; occurrenceID: F1CE0E5B-A2AE-56EC-91A5-00D3E8AC33AF; **Taxon:** scientificName: *Coelophthinialoraasi*; order: Diptera; family: Mycetophilidae; genus: Coelophthinia; specificEpithet: *loraasi*; scientificNameAuthorship: Kjaerandsen; **Location:** country: Norway; stateProvince: Nordland (NSI); municipality: Grane; locality: Stormobekken; decimalLatitude: 65.595; decimalLongitude: 13.40333; coordinateUncertaintyInMeters: 10; **Identification:** identifiedBy: J. Kjærandsen; dateIdentified: Aug-06-2020; **Event:** samplingProtocol: window trap; eventDate: 2018-07-31 to 2018-10-05; eventRemarks: WT 3; **Record Level:** institutionCode: TSZ; collectionCode: TMU-JKJ-COL-000799; basisOfRecord: Preserved specimen
- **Type status:** Other material. **Occurrence:** catalogNumber: TSZD-JKJ-112072; recordedBy: J. Kjærandsen, J. P. Lindemann & P. Dominiak; individualCount: 1; sex: male; lifeStage: imago; preparations: Pinned (HMDS-dried from ethanol); associatedOccurrences: urn:uuid:cfb394b3-6acd-4a65-9d60-d1d950561db9; occurrenceID: 3B9D7021-8E22-5759-9A13-690E5EF4768A; **Taxon:** scientificName: *Coelophthinialoraasi*; order: Diptera; family: Mycetophilidae; genus: Coelophthinia; specificEpithet: *loraasi*; scientificNameAuthorship: Kjaerandsen; **Location:** country: Norway; stateProvince: Nordland (NSI); municipality: Grane; locality: Stormobekken; decimalLatitude: 65.595; decimalLongitude: 13.40333; coordinateUncertaintyInMeters: 10; **Identification:** identifiedBy: J. Kjærandsen; dateIdentified: Aug-06-2020; **Event:** samplingProtocol: window trap; eventDate: 2018-07-31 to 2018-10-05; eventRemarks: WT 3; **Record Level:** institutionCode: TSZ; collectionCode: TMU-JKJ-COL-000799; basisOfRecord: Preserved specimen

#### Description

Male (Fig. 7, n = 3 for measurements).

Coloration and general body characteristics as in the genus description. Body length 3.5–3.6 mm. Wing length 2.8–2.8 mm; ratio of length to width 2.6. Sensory organ dorsally on the basal half of mid-tibia elongate oval, 6.1 times longer than wide, length 0.20 that of tibial length.

Terminalia. Tergite 9 rectangular in dorsal view, about 1.5 times as long as wide, curved like a hood in lateral view. Medial protrusion of tergite 10 thin, height approximately 3x width in posterior view, curved posteriad, densely setose on apex and ventral side. Gonocoxites in lateral view with a narrow subsquare outline, with nearly straight ventral margin and posterolateral lobe situated along the ventral edge. Posterolateral lobe of gonocoxite long, slender and evenly tapering, about 4x longer than width at apex, rounded apically, laterally bare for about 2x apical width. Spathulate gonocoxal lobe about five times as long as wide, rounded, with distinctly constricted base, basad of base with a row of 5 long setae. Dorsal branch of gonostylus small, simple, semicircular, laterally setose with some extra long setae. The broad lobe 1 (cf. Fig. 3f) fan-shaped, carrying 8 blunt setae along rim and 5 normal setae subapically on inner side. The narrow, acute tipped lobe 2 (cf. Fig. 3f) with a tiny stiff seta subapically; with strong, blunt seta placed at its length to the tip; followed by one short and one long seta in mid-section. Aedeagus 1.5x longer than spathulate gonocoxal lobe in lateral view, evenly broad until apically sharply narrowed downcurved hook; apically with a short spike at the outer side below the curving point; downcurved hook more than twice as long as breadth of base of aedeagus.

Female (Fig. 8, n = 2 for measurements).

Coloration as for male. Body length 3.9–3.9 mm. Wing length 2.9–3.0 mm; ratio of length to width 2.8. Sensory organ dorsally on the basal half of mid-tibia elongate oval, 5.5 times longer than wide, 0.32 times of tibial length.

Terminalia as described for genus. Cercus slightly narrower than in other species, with fewer setae on ventral side. Gonapophysis 8 forming an equilateral triangle in ventral view. Sternite 9 forming a distinct circle in ventral view.

#### Diagnosis

*Coelophthinialoraasi* can be distinguished from other species of the genus by the combination of having a narrow, rectangular shape of the gonocoxite as seen in lateral view, with a near straight ventral margin, a narrow and long posteriolateral lobe and a long, narrow and curved medial protrusion of tergite 10. The aedeagus is uniquely shaped with apical hook much longer than the height of the base of aedeagus. The length of the mid-tibial organ is about 1/6 of tibial length.

#### Etymology

Named in honour of Professor Jostein Lorås, the local biologist who, for many years, has worked hard for and succeeded to protect areas of old growth coniferous forests in Grane Municipality of Nordland County, including those from where the type materials originate.

#### Distribution

So far known only from northern Norway.

### Coelophthinia itoae

Kurina sp. nov.

763CA553-05B4-586A-B042-13378766914F

http://www.boldsystems.org/index.php/Public_BarcodeCluster?clusteruri=BOLD:ADY9337

0CDADA2C-69FF-4479-88B1-D69D7BF1ECC2

#### Materials

- **Type status:** Holotype. **Occurrence:** catalogNumber: IZBE0251605; recordedBy: Olavi Kurina; individualCount: 1; sex: male; preparations: slide mounted in Euparal; terminalia in glycerine in separate microvial; occurrenceID: ECFA61EE-9402-54B6-923C-ED7DAEB0CC79; **Taxon:** scientificName: *Coelophthiniaitoae*; order: Diptera; family: Mycetophilidae; genus: Coelophthinia; specificEpithet: *itoae*; scientificNameAuthorship: Kurina; **Location:** country: Japan; stateProvince: HOKKAIDO: Hokkaido Prefecture; county: Kushiro-shi; municipality: Kushiro-shi; locality: Upper reach of Ibeshibetsu river, nearLake Akan, Akan-cho; verbatimElevation: 448 m; decimalLatitude: 43.4891; decimalLongitude: 144.1477; **Identification:** identifiedBy: Olavi Kurina; **Event:** samplingProtocol: sweep net; eventDate: 2006-10-03; **Record Level:** basisOfRecord: Preserved specimen
- **Type status:** Paratype. **Occurrence:** catalogNumber: IZBE0251606; recordedBy: Olavi Kurina; individualCount: 1; sex: male; preparations: slide mounted in Euparal; terminalia in glycerine in separate microvial; occurrenceID: 47CE4ED8-BB2B-5256-962C-8E9F8204E75B; **Taxon:** scientificName: *Coelophthiniaitoae*; order: Diptera; family: Mycetophilidae; genus: Coelophthinia; specificEpithet: *itoae*; scientificNameAuthorship: Kurina; **Location:** country: Japan; stateProvince: HOKKAIDO: Hokkaido Prefecture; municipality: Kushiro-shi; locality: Upper reach of Ibeshibetsu river, nearLake Akan, Akan-cho; verbatimElevation: 448 m; decimalLatitude: 43.4891; decimalLongitude: 144.1477; **Identification:** identifiedBy: Olavi Kurina; **Event:** samplingProtocol: sweep net; eventDate: 2006-10-03; **Record Level:** basisOfRecord: Preserved specimen
- **Type status:** Paratype. **Occurrence:** catalogNumber: IZBE0251607 & TSZD-JKJ-106937 (BOLD voucher); recordedBy: Olavi Kurina; individualCount: 1; sex: female; preparations: pinned, mounted from ethanol; terminalia in glycerine in separate microvial; occurrenceID: 850DA64F-51D7-502C-AE32-0BD4FEAD130B; **Taxon:** scientificName: *Coelophthiniaitoae*; order: Diptera; family: Mycetophilidae; genus: Coelophthinia; specificEpithet: *itoae*; scientificNameAuthorship: Kurina; **Location:** country: Japan; stateProvince: HOKKAIDO: Hokkaido Prefecture; municipality: Kushiro-shi; locality: Lower reach of Ibeshibetsu river, near Lake Akan, Akan-cho; verbatimElevation: 448 m; decimalLatitude: 43.4808; decimalLongitude: 144.1277; **Identification:** identifiedBy: Olavi Kurina; **Event:** samplingProtocol: sweep net; eventDate: 2006-10-04; **Record Level:** basisOfRecord: Preserved specimen
- **Type status:** Paratype. **Occurrence:** catalogNumber: TSZD-JKJ-102075; recordedBy: J. Kjærandsen; individualCount: 1; sex: male; lifeStage: adult; preparations: Pinned (HMDS-dried from ethanol); associatedOccurrences: urn:uuid:b26051ad-2aad-49f6-97b4-02f1d7836785; occurrenceID: 0FB19303-C857-5587-B6D7-6CB8E3F0826E; **Taxon:** scientificName: *Coelophthiniaitoae*; order: Diptera; family: Mycetophilidae; genus: Coelophthinia; specificEpithet: *itoae*; scientificNameAuthorship: Kurina; **Location:** country: Japan; stateProvince: HOKKAIDO: Hokkaido Prefecture; municipality: Chitose-shi; locality: Bifue-gawa at Bifue-no-taki falls; minimumElevationInMeters: 375; decimalLatitude: 42.72694; decimalLongitude: 141.19222; coordinateUncertaintyInMeters: 100; **Identification:** identifiedBy: J. Kjærandsen; dateIdentified: Sep-22-2007; **Event:** samplingProtocol: sweep net; eventDate: 2006-10-02; **Record Level:** institutionCode: TSZ; collectionCode: COL-003263
- **Type status:** Paratype. **Occurrence:** catalogNumber: TSZD-JKJ-111450; recordedBy: J. Kjærandsen; individualCount: 1; sex: female; lifeStage: adult; preparations: Pinned (HMDS-dried from ethanol); associatedOccurrences: urn:uuid:496836f0-e75d-4b59-99fa-36c44f47ec12; occurrenceID: 10142870-BF28-555F-9B5A-02B6395959C3; **Taxon:** scientificName: *Coelophthiniaitoae*; order: Diptera; family: Mycetophilidae; genus: Coelophthinia; specificEpithet: *itoae*; scientificNameAuthorship: Kurina; **Location:** country: Japan; stateProvince: HOKKAIDO: Hokkaido Prefecture; municipality: Kushiro-shi; locality: Middle-lower reach of Ibeshibetsu River near Lake Akan, Akan-cho; minimumElevationInMeters: 427; decimalLatitude: 43.48083; decimalLongitude: 144.13917; coordinateUncertaintyInMeters: 250; **Identification:** identifiedBy: J. Kjærandsen; dateIdentified: Sep-25-2020; **Event:** samplingProtocol: sweep net; eventDate: 2006-10-04; eventRemarks: Site 2; **Record Level:** institutionCode: TSZ - BOLD voucher; collectionCode: COL-003271
- **Type status:** Other material. **Occurrence:** catalogNumber: TSZD-JKJ-111497; recordedBy: J. Kjærandsen; individualCount: 1; sex: female; lifeStage: adult; preparations: 80% alc.; associatedOccurrences: urn:uuid:c2186363-d6ce-4fb3-a92f-5d3fb9c49ce5; occurrenceID: 939DC69F-ABCF-56B7-B4DB-649FCA9933B7; **Taxon:** scientificName: *Coelophthiniaitoae*; order: Diptera; family: Mycetophilidae; genus: Coelophthinia; specificEpithet: *itoae*; scientificNameAuthorship: Kurina; **Location:** country: Japan; stateProvince: HOKKAIDO: Hokkaido Prefecture; municipality: Kushiro-shi; locality: Middle-lower reach of Ibeshibetsu River near Lake Akan, Akan-cho; minimumElevationInMeters: 427; decimalLatitude: 43.48083; decimalLongitude: 144.13917; coordinateUncertaintyInMeters: 250; **Identification:** identifiedBy: J. Kjærandsen; dateIdentified: Sep-11-2020; **Event:** samplingProtocol: sweep net; eventDate: 2006-10-04; eventRemarks: Site 2; **Record Level:** institutionCode: TSZ; collectionCode: COL-003271

#### Description

Male. (Figs 9, 10)

Coloration and most body characteristics as in the genus description. Body length 3.2 mm. Wing length 2.70–2.77 mm; ratio of length to width 1.9–2.5. Sensory organ dorsally on the basal half of mid-tibia elongate oval, 6.7 times longer than wide, length 0.34 that of tibial length.

Terminalia. Tergite 9 rectangular in dorsal view, about 1.25 times as long as wide, curved like a hood in lateral view. Medial protrusion of tergite 10 remarkably long, height approximately 4-5x width in posterior view, curved posteriad, densely setose. Gonocoxites in lateral view with a rounded outline, with slightly convex ventral margin. Posterolateral lobe of gonocoxite short and wide, only somewhat longer than wide, truncated apically, laterally bare for about 1.5x apical width. Spathulate gonocoxal lobe about 3 times as long as wide, curved slightly medially, without constricted base, basad of base with a group of 3–4 longer setae. Dorsal branch small, simple, wider than long, apically rounded, dorsally setose, with one extra long seta deviating from others. The broad lobe 1 (cf. Fig. 9d) transversally extended, carrying 6 blunt setae along rim and 5 normal very long setae subapically on inner side. The narrow, acute tipped lobe 2 (cf. Fig. 9d) tapering, with strong, blunt seta placed subapically; with one short and one very long seta in mid-section. Aedeagus 1.5x longer than spathulate gonocoxal lobe in lateral view, with swelling medially; apically narrowed into downcurved hook; apically with a spike at the outer side at the curving point; downcurved hook about three times of width of aedeagus basally.

Female. (Fig. 11)

Coloration as for male. Sensory organ dorsally on the basal half of mid-tibia elongate oval, 9.2 times longer than wide, length 0.32 that of tibial length.

Terminalia as described for genus. Tergite 8 short, posteriorly emarginated in dorsal view, bare. Tergite 9 short, posteriorly emarginated in dorsal view, bare, except posterior margin with row of 7–8 long setae, which are longer than those on cercus. Cercus about two times as long as wide medially, evenly covered with setae. Sternite 8 longer than tergites 8 and 9 together, posterolaterally rounded, posterior half setose, in ventral view hypogynal valves separated by V-shaped deep incision, about one-third as deep as segment length. Gonapophysis 8 elongate, about 8 times as long as wide, extending to mid-cercus, setose, with one deviating strong subapical seta.

#### Diagnosis

*Coelophthiniaitoae* can be distinguished from other species of the genus by the combination of having a rounded shape of the gonocoxite as seen in lateral view, with a slightly convex ventral margin, a short and wide posterolateral lobe and a long and posteriad curved medial protrusion of tergite 10. The ventral branch of the gonostylus with broad lobe transversally extended. The aedeagus is uniquely shaped, short, with delimited swelling medially and with apical hook about three times longer than the width of the base of aedeagus. The length of the mid-tibial organ is about 0.3 of tibial length.

#### Etymology

Named after Tomiko Ito, who kindly guided JK and OK on a collecting trip around Hokkaidō, Japan, in 2006, when this species was collected and discovered.

#### Distribution

Eastern Palaearctic: Japan (Hokkaidō, Honshū).

### Coelophthinia curta

(Johannsen, 1912)

0A67908C-0CF1-5F0C-B1A8-28C661711D1A

http://sciaroidea.info/taxonomy/47769

http://www.boldsystems.org/index.php/Public_BarcodeCluster?clusteruri=BOLD:ACI7210

http://www.boldsystems.org/index.php/Public_BarcodeCluster?clusteruri=BOLD:AAM9005

*Phthiniacurta* Johannsen, 1912

#### Materials

- **Type status:** Other material. **Occurrence:** catalogNumber: 12J163; recordedBy: Peter H. Kerr; individualCount: 1; sex: male; lifeStage: Adult; occurrenceID: 4687B2F6-B274-5D61-998B-906EA0CF1550; **Taxon:** scientificName: *Coelophthiniacurta*; order: Diptera; family: Mycetophilidae; genus: Coelophthinia; specificEpithet: *curta*; scientificNameAuthorship: (Johannsen); **Location:** country: United States; countryCode: USA; stateProvince: California; county: Alpine; locality: Grover Hot Springs St. Pk.; verbatimLocality: nr. Hoffman house; verbatimElevation: 1800m; verbatimCoordinateSystem: decimal degrees; decimalLatitude: 38.6952; decimalLongitude: -119.838; **Identification:** identificationID: 12J163; identifiedBy: Peter H. Kerr; dateIdentified: 04/23/2012; **Event:** eventID: 06LOT476; samplingProtocol: Malaise trap (2m); eventDate: 14.viii–3.ix.2006; **Record Level:** type: Preserved Specimen; collectionID: urn:lsid:biocol.org:col:33169; institutionCode: CSCA; basisOfRecord: PreservedSpecimen
- **Type status:** Other material. **Occurrence:** catalogNumber: 07Y536; recordedBy: Peter H. Kerr; individualCount: 1; sex: male; lifeStage: Adult; occurrenceID: 839E40EC-E712-5B07-96F1-6AFDA24D1AC5; **Taxon:** scientificName: *Coelophthiniacurta*; order: Diptera; family: Mycetophilidae; genus: Coelophthinia; specificEpithet: *curta*; scientificNameAuthorship: (Johannsen); **Location:** country: United States; countryCode: USA; stateProvince: California; county: Sonoma; locality: Annadel State Park; verbatimLocality: ravine nr. Warren Richardson trail; verbatimElevation: 220m; verbatimCoordinateSystem: decimal degrees; decimalLatitude: 38.4351; decimalLongitude: -122.6111; **Identification:** identificationID: 07Y536; identifiedBy: Peter H. Kerr; dateIdentified: 08/07/2007; **Event:** eventID: 07LOT196; samplingProtocol: Malaise trap (6m); eventDate: 17.v–7.vi.2007; **Record Level:** type: Preserved Specimen; collectionID: urn:lsid:biocol.org:col:33156; institutionCode: CSCA; basisOfRecord: PreservedSpecimen
- **Type status:** Other material. **Occurrence:** catalogNumber: 11G706; recordedBy: Peter H. Kerr; individualCount: 1; sex: male; lifeStage: Adult; occurrenceID: BC5DA562-7E14-5546-9568-23359E800E2F; **Taxon:** scientificName: *Coelophthiniacurta*; order: Diptera; family: Mycetophilidae; genus: Coelophthinia; specificEpithet: *curta*; scientificNameAuthorship: (Johannsen); **Location:** country: United States; countryCode: USA; stateProvince: California; county: Sonoma; locality: Annadel State Park; verbatimLocality: ravine nr. Warren Richardson trail; verbatimElevation: 220m; verbatimCoordinateSystem: decimal degrees; decimalLatitude: 38.4351; decimalLongitude: -122.6111; **Identification:** identificationID: 11G706; identifiedBy: Peter H. Kerr; dateIdentified: 06/01/2011; **Event:** eventID: 07LOT196; samplingProtocol: Malaise trap (6m); eventDate: 17.v–7.vi.2007; **Record Level:** type: Preserved Specimen; collectionID: urn:lsid:biocol.org:col:33163; institutionCode: CSCA; basisOfRecord: PreservedSpecimen
- **Type status:** Other material. **Occurrence:** catalogNumber: 11G707; recordedBy: Peter H. Kerr; individualCount: 1; sex: male; lifeStage: Adult; occurrenceID: FA87D9B0-7E17-5C0A-A189-0888A731C692; **Taxon:** scientificName: *Coelophthiniacurta*; order: Diptera; family: Mycetophilidae; genus: Coelophthinia; specificEpithet: *curta*; scientificNameAuthorship: (Johannsen); **Location:** country: United States; countryCode: USA; stateProvince: California; county: Humboldt; locality: Humboldt Bay National Wildlife Refuge; verbatimLocality: Lanphere Dunes; verbatimElevation: 6m; verbatimCoordinateSystem: decimal degrees; decimalLatitude: 40.8914; decimalLongitude: -124.143; **Identification:** identificationID: 11G707; identifiedBy: Peter H. Kerr; dateIdentified: 06/01/2011; **Event:** eventID: 07LOT636; samplingProtocol: Malaise trap (6m); eventDate: 28.ix–2.xi.2007; **Record Level:** type: Preserved Specimen; collectionID: urn:lsid:biocol.org:col:33164; institutionCode: CSCA; basisOfRecord: PreservedSpecimen
- **Type status:** Other material. **Occurrence:** catalogNumber: 11G708; recordedBy: Peter H. Kerr; individualCount: 1; sex: male; lifeStage: Adult; occurrenceID: AA06313A-D072-5FF6-976B-CAD345AE6897; **Taxon:** scientificName: *Coelophthiniacurta*; order: Diptera; family: Mycetophilidae; genus: Coelophthinia; specificEpithet: *curta*; scientificNameAuthorship: (Johannsen); **Location:** country: United States; countryCode: USA; stateProvince: California; county: Sonoma; locality: Annadel State Park; verbatimLocality: ravine nr. Warren Richardson trail; verbatimElevation: 220m; verbatimCoordinateSystem: decimal degrees; decimalLatitude: 38.4351; decimalLongitude: -122.6111; **Identification:** identificationID: 11G708; identifiedBy: Peter H. Kerr; dateIdentified: 06/01/2011; **Event:** eventID: 07LOT762; samplingProtocol: Malaise trap (6m); eventDate: 29.xi.2007-10.i.2008; **Record Level:** type: Preserved Specimen; collectionID: urn:lsid:biocol.org:col:33165; institutionCode: CSCA; basisOfRecord: PreservedSpecimen
- **Type status:** Other material. **Occurrence:** catalogNumber: 11G778; recordedBy: Peter H. Kerr; individualCount: 1; sex: male; lifeStage: Adult; occurrenceID: F02E1752-791A-5784-900E-3300D450B987; **Taxon:** scientificName: *Coelophthiniacurta*; order: Diptera; family: Mycetophilidae; genus: Coelophthinia; specificEpithet: *curta*; scientificNameAuthorship: (Johannsen); **Location:** country: United States; countryCode: USA; stateProvince: California; county: Sonoma; locality: Annadel State Park; verbatimLocality: ravine nr. Warren Richardson trail; verbatimElevation: 220m; verbatimCoordinateSystem: decimal degrees; decimalLatitude: 38.4351; decimalLongitude: -122.6111; **Identification:** identificationID: 11G778; identifiedBy: Peter H. Kerr; dateIdentified: 08/03/2011; **Event:** eventID: 07LOT762; samplingProtocol: Malaise trap (6m); eventDate: 29.xi.2007-10.i.2008; **Record Level:** type: Preserved Specimen; collectionID: urn:lsid:biocol.org:col:33167; institutionCode: CSCA; basisOfRecord: PreservedSpecimen
- **Type status:** Other material. **Occurrence:** catalogNumber: 11G779; recordedBy: Peter H. Kerr; individualCount: 1; sex: male; lifeStage: Adult; occurrenceID: A4F93DFF-EB3F-59F2-AC84-74D132E51EE4; **Taxon:** scientificName: *Coelophthiniacurta*; order: Diptera; family: Mycetophilidae; genus: Coelophthinia; specificEpithet: *curta*; scientificNameAuthorship: (Johannsen); **Location:** country: United States; countryCode: USA; stateProvince: California; county: Sonoma; locality: Annadel State Park; verbatimLocality: ravine nr. Warren Richardson trail; verbatimElevation: 220m; verbatimCoordinateSystem: decimal degrees; decimalLatitude: 38.4351; decimalLongitude: -122.6111; **Identification:** identificationID: 11G779; identifiedBy: Peter H. Kerr; dateIdentified: 08/03/2011; **Event:** eventID: 07LOT762; samplingProtocol: Malaise trap (6m); eventDate: 29.xi.2007-10.i.2008; **Record Level:** type: Preserved Specimen; collectionID: urn:lsid:biocol.org:col:33168; institutionCode: CSCA; basisOfRecord: PreservedSpecimen
- **Type status:** Other material. **Occurrence:** catalogNumber: 12K705; recordedBy: Peter H. Kerr; individualCount: 1; sex: male; lifeStage: Adult; occurrenceID: E926E5CD-904C-5236-938F-B7BFBEC51AC9; **Taxon:** scientificName: *Coelophthiniacurta*; order: Diptera; family: Mycetophilidae; genus: Coelophthinia; specificEpithet: *curta*; scientificNameAuthorship: (Johannsen); **Location:** country: United States; countryCode: USA; stateProvince: California; county: Humboldt; locality: Patrick’s Point State Park; verbatimLocality: forest behind visitor center; verbatimElevation: 10m; verbatimCoordinateSystem: decimal degrees; decimalLatitude: 41.1351; decimalLongitude: -124.1546; **Identification:** identificationID: 12K705; identifiedBy: Peter H. Kerr; dateIdentified: 11/28/2012; **Event:** eventID: 07LOT816; samplingProtocol: Malaise trap (6m); eventDate: 11.xi-19.xii.2007; **Record Level:** type: Preserved Specimen; collectionID: urn:lsid:biocol.org:col:33175; institutionCode: CSCA; basisOfRecord: PreservedSpecimen
- **Type status:** Other material. **Occurrence:** catalogNumber: 20i608; recordedBy: Peter H. Kerr; individualCount: 1; sex: male; lifeStage: Adult; occurrenceID: 23683977-097A-58AF-B31B-B710CFAB63F3; **Taxon:** scientificName: *Coelophthiniacurta*; order: Diptera; family: Mycetophilidae; genus: Coelophthinia; specificEpithet: *curta*; scientificNameAuthorship: (Johannsen); **Location:** country: United States; countryCode: USA; stateProvince: California; county: Humboldt; locality: Patrick’s Point State Park; verbatimLocality: forest behind visitor center; verbatimElevation: 10m; verbatimCoordinateSystem: decimal degrees; decimalLatitude: 41.1351; decimalLongitude: -124.1546; **Identification:** identificationID: 20i608; identifiedBy: Peter H. Kerr; dateIdentified: 11/20/2020; **Event:** eventID: 07LOT816; samplingProtocol: Malaise trap (6m); eventDate: 11.xi-19.xii.2007; **Record Level:** type: Preserved Specimen; collectionID: urn:lsid:biocol.org:col:33188; institutionCode: CSCA; basisOfRecord: PreservedSpecimen
- **Type status:** Other material. **Occurrence:** catalogNumber: 21M154; recordedBy: Peter H. Kerr; individualCount: 1; sex: male; lifeStage: Adult; occurrenceID: 86BB8BA2-7AED-594F-A612-2E1AB8B9EDEC; **Taxon:** scientificName: *Coelophthiniacurta*; order: Diptera; family: Mycetophilidae; genus: Coelophthinia; specificEpithet: *curta*; scientificNameAuthorship: (Johannsen); **Location:** country: United States; countryCode: USA; stateProvince: California; county: Humboldt; locality: Patrick’s Point State Park; verbatimLocality: forest behind visitor center; verbatimElevation: 10m; verbatimCoordinateSystem: decimal degrees; decimalLatitude: 41.1351; decimalLongitude: -124.1546; **Identification:** identificationID: 21M154; identifiedBy: Peter H. Kerr; dateIdentified: 03/01/2021; **Event:** eventID: 07LOT816; samplingProtocol: Malaise trap (6m); eventDate: 11.xi-19.xii.2007; **Record Level:** type: Preserved Specimen; collectionID: urn:lsid:biocol.org:col:33192; institutionCode: CSCA; basisOfRecord: PreservedSpecimen
- **Type status:** Other material. **Occurrence:** catalogNumber: 10F088; recordedBy: Peter H. Kerr; individualCount: 1; sex: male; lifeStage: Adult; occurrenceID: 0D04A3E9-ED66-5F96-8D1E-7111A3118BA0; **Taxon:** scientificName: *Coelophthiniacurta*; order: Diptera; family: Mycetophilidae; genus: Coelophthinia; specificEpithet: *curta*; scientificNameAuthorship: (Johannsen); **Location:** country: United States; countryCode: USA; stateProvince: California; county: Sonoma; locality: Annadel State Park; verbatimLocality: ravine nr. Warren Richardson trail; verbatimElevation: 220m; verbatimCoordinateSystem: decimal degrees; decimalLatitude: 38.4351; decimalLongitude: -122.6111; **Identification:** identificationID: 10F088; identifiedBy: Peter H. Kerr; dateIdentified: 03/08/2010; **Event:** eventID: 08LOT008; samplingProtocol: Malaise trap (6m); eventDate: 10.i-18.iii.2008; **Record Level:** type: Preserved Specimen; collectionID: urn:lsid:biocol.org:col:33159; institutionCode: CSCA; basisOfRecord: PreservedSpecimen
- **Type status:** Other material. **Occurrence:** catalogNumber: 10F749; recordedBy: Peter H. Kerr; individualCount: 1; sex: male; lifeStage: Adult; occurrenceID: 561008A1-A56B-58E4-B57B-502B5C283609; **Taxon:** scientificName: *Coelophthiniacurta*; order: Diptera; family: Mycetophilidae; genus: Coelophthinia; specificEpithet: *curta*; scientificNameAuthorship: (Johannsen); **Location:** country: United States; countryCode: USA; stateProvince: California; county: Sonoma; locality: Annadel State Park; verbatimLocality: ravine nr. Warren Richardson trail; verbatimElevation: 220m; verbatimCoordinateSystem: decimal degrees; decimalLatitude: 38.4351; decimalLongitude: -122.6111; **Identification:** identificationID: 10F749; identifiedBy: Peter H. Kerr; dateIdentified: 08/03/2010; **Event:** eventID: 08LOT008; samplingProtocol: Malaise trap (6m); eventDate: 10.i-18.iii.2008; **Record Level:** type: Preserved Specimen; collectionID: urn:lsid:biocol.org:col:33160; institutionCode: CSCA; basisOfRecord: PreservedSpecimen
- **Type status:** Other material. **Occurrence:** catalogNumber: 20i609; recordedBy: Peter H. Kerr; individualCount: 1; sex: male; lifeStage: Adult; occurrenceID: B4C3D13C-30D6-54BF-8E2E-D41E7DB8ED9C; **Taxon:** scientificName: *Coelophthiniacurta*; order: Diptera; family: Mycetophilidae; genus: Coelophthinia; specificEpithet: *curta*; scientificNameAuthorship: (Johannsen); **Location:** country: United States; countryCode: USA; stateProvince: California; county: Sonoma; locality: Annadel State Park; verbatimLocality: ravine nr. Warren Richardson trail; verbatimElevation: 220m; verbatimCoordinateSystem: decimal degrees; decimalLatitude: 38.4351; decimalLongitude: -122.6111; **Identification:** identificationID: 20i609; identifiedBy: Peter H. Kerr; dateIdentified: 11/20/2020; **Event:** eventID: 08LOT008; samplingProtocol: Malaise trap (6m); eventDate: 10.i-18.iii.2008; **Record Level:** type: Preserved Specimen; collectionID: urn:lsid:biocol.org:col:33189; institutionCode: CSCA; basisOfRecord: PreservedSpecimen
- **Type status:** Other material. **Occurrence:** catalogNumber: 21P218; recordedBy: Peter H. Kerr; individualCount: 1; sex: male; lifeStage: Adult; occurrenceID: A1EA7117-CFA7-57A8-A29B-0464FAB0D72F; **Taxon:** scientificName: *Coelophthiniacurta*; order: Diptera; family: Mycetophilidae; genus: Coelophthinia; specificEpithet: *curta*; scientificNameAuthorship: (Johannsen); **Location:** country: United States; countryCode: USA; stateProvince: California; county: Sonoma; locality: Annadel State Park; verbatimLocality: ravine nr. Warren Richardson trail; verbatimElevation: 220m; verbatimCoordinateSystem: decimal degrees; decimalLatitude: 38.4351; decimalLongitude: -122.6111; **Identification:** identificationID: 21P218; identifiedBy: Peter H. Kerr; dateIdentified: 11/24/2021; **Event:** eventID: 08LOT008; samplingProtocol: Malaise trap (6m); eventDate: 10.i-18.iii.2008; **Record Level:** type: Preserved Specimen; collectionID: urn:lsid:biocol.org:col:33196; institutionCode: CSCA; basisOfRecord: PreservedSpecimen
- **Type status:** Other material. **Occurrence:** catalogNumber: 21M584; recordedBy: Peter H. Kerr; individualCount: 1; sex: male; lifeStage: Adult; occurrenceID: 069E4CDC-C46C-5BFC-BD90-3AB8B94E3F65; **Taxon:** scientificName: *Coelophthiniacurta*; order: Diptera; family: Mycetophilidae; genus: Coelophthinia; specificEpithet: *curta*; scientificNameAuthorship: (Johannsen); **Location:** country: United States; countryCode: USA; stateProvince: California; county: Sonoma; locality: Annadel State Park; verbatimLocality: ravine nr. Warren Richardson trail; verbatimElevation: 220m; verbatimCoordinateSystem: decimal degrees; decimalLatitude: 38.4351; decimalLongitude: -122.6111; **Identification:** identificationID: 21M584; identifiedBy: Peter H. Kerr; dateIdentified: 03/12/2021; **Event:** eventID: CSCA08L283; samplingProtocol: Malaise trap (6m); eventDate: 18.iii-2.v.2008; **Record Level:** type: Preserved Specimen; collectionID: urn:lsid:biocol.org:col:33193; institutionCode: CSCA; basisOfRecord: PreservedSpecimen
- **Type status:** Other material. **Occurrence:** catalogNumber: 12J164; recordedBy: Peter H. Kerr; individualCount: 1; sex: male; lifeStage: Adult; occurrenceID: DCB4283F-3756-5890-B132-6D4CF777321A; **Taxon:** scientificName: *Coelophthiniacurta*; order: Diptera; family: Mycetophilidae; genus: Coelophthinia; specificEpithet: *curta*; scientificNameAuthorship: (Johannsen); **Location:** country: United States; countryCode: USA; stateProvince: California; county: Humboldt; locality: Patrick’s Point State Park; verbatimLocality: forest behind visitor center; verbatimElevation: 10m; verbatimCoordinateSystem: decimal degrees; decimalLatitude: 41.1351; decimalLongitude: -124.1546; **Identification:** identificationID: 12J164; identifiedBy: Peter H. Kerr; dateIdentified: 04/25/2012; **Event:** eventID: CSCA08L358; samplingProtocol: Malaise trap (6m); eventDate: 19.xii.07-3.iii.2008; **Record Level:** type: Preserved Specimen; collectionID: urn:lsid:biocol.org:col:33170; institutionCode: CSCA; basisOfRecord: PreservedSpecimen
- **Type status:** Other material. **Occurrence:** catalogNumber: 20i432; recordedBy: Peter H. Kerr; individualCount: 1; sex: male; lifeStage: Adult; occurrenceID: 5EDF84C1-0634-565A-BF0B-AB2D3A89E39D; **Taxon:** scientificName: *Coelophthiniacurta*; order: Diptera; family: Mycetophilidae; genus: Coelophthinia; specificEpithet: *curta*; scientificNameAuthorship: (Johannsen); **Location:** country: United States; countryCode: USA; stateProvince: California; county: Humboldt; locality: Patrick’s Point State Park; verbatimLocality: forest behind visitor center; verbatimElevation: 10m; verbatimCoordinateSystem: decimal degrees; decimalLatitude: 41.1351; decimalLongitude: -124.1546; **Identification:** identificationID: 20i432; identifiedBy: Peter H. Kerr; dateIdentified: 10/20/2020; **Event:** eventID: CSCA08L358; samplingProtocol: Malaise trap (6m); eventDate: 19.xii.07-3.iii.2008; **Record Level:** type: Preserved Specimen; collectionID: urn:lsid:biocol.org:col:33187; institutionCode: CSCA; basisOfRecord: PreservedSpecimen
- **Type status:** Other material. **Occurrence:** catalogNumber: 21P217; recordedBy: Peter H. Kerr; individualCount: 1; sex: male; lifeStage: Adult; occurrenceID: 1C6BE6CC-A75F-5603-979C-FEB071F2DC4C; **Taxon:** scientificName: *Coelophthiniacurta*; order: Diptera; family: Mycetophilidae; genus: Coelophthinia; specificEpithet: *curta*; scientificNameAuthorship: (Johannsen); **Location:** country: United States; countryCode: USA; stateProvince: California; county: Amador; locality: Indian Grinding Rock State Historical Park; verbatimLocality: dry wash/stream bed within South Nature trail; verbatimElevation: 715m; verbatimCoordinateSystem: decimal degrees; decimalLatitude: 38.4216; decimalLongitude: -120.645; **Identification:** identificationID: 21P217; identifiedBy: Peter H. Kerr; dateIdentified: 11/24/2021; **Event:** eventID: CSCA08L596; samplingProtocol: Malaise trap (6m); eventDate: 15.v–18.vi.2008; **Record Level:** type: Preserved Specimen; collectionID: urn:lsid:biocol.org:col:33195; institutionCode: CSCA; basisOfRecord: PreservedSpecimen
- **Type status:** Other material. **Occurrence:** catalogNumber: 09D923; recordedBy: Peter H. Kerr; individualCount: 1; sex: male; lifeStage: Adult; occurrenceID: AC53A3EC-CAB4-5D77-BDF7-5EBF65F438D4; **Taxon:** scientificName: *Coelophthiniacurta*; order: Diptera; family: Mycetophilidae; genus: Coelophthinia; specificEpithet: *curta*; scientificNameAuthorship: (Johannsen); **Location:** country: United States; countryCode: USA; stateProvince: California; county: Humboldt; locality: Redwoods National Park; verbatimLocality: Redwood Crk Rd., 100m E. Bald Hills Rd.; verbatimElevation: 15m; verbatimCoordinateSystem: decimal degrees; decimalLatitude: 41.3023; decimalLongitude: -124.0406; **Identification:** identificationID: 09D923; identifiedBy: Peter H. Kerr; dateIdentified: 09/16/2009; **Event:** eventID: CSCA09L522; samplingProtocol: Malaise trap (2m); eventDate: 2.vi-25.vii.2009; **Record Level:** type: Preserved Specimen; collectionID: urn:lsid:biocol.org:col:33157; institutionCode: CSCA; basisOfRecord: PreservedSpecimen
- **Type status:** Other material. **Occurrence:** catalogNumber: 09E154; recordedBy: Peter H. Kerr; individualCount: 1; sex: male; lifeStage: Adult; occurrenceID: 2E93AB5B-25DA-5CF8-920A-27D5B0F78DAE; **Taxon:** scientificName: *Coelophthiniacurta*; order: Diptera; family: Mycetophilidae; genus: Coelophthinia; specificEpithet: *curta*; scientificNameAuthorship: (Johannsen); **Location:** country: United States; countryCode: USA; stateProvince: California; county: Del Norte; locality: Six Rivers National Forest; verbatimLocality: ForRoute16N02, 0.3miE BearBasin Rd., nr. BearBasin Outlook; verbatimElevation: 1500m; verbatimCoordinateSystem: decimal degrees; decimalLatitude: 41.8016; decimalLongitude: -123.7369; **Identification:** identificationID: 09E154; identifiedBy: Peter H. Kerr; dateIdentified: 09/29/2009; **Event:** eventID: CSCA09L526; samplingProtocol: Malaise trap (6m); eventDate: 3.vi–24.vii.2009; **Record Level:** type: Preserved Specimen; collectionID: urn:lsid:biocol.org:col:33158; institutionCode: CSCA; basisOfRecord: PreservedSpecimen
- **Type status:** Other material. **Occurrence:** catalogNumber: 13M168; recordedBy: Peter H. Kerr; individualCount: 3; sex: male; lifeStage: Adult; occurrenceID: 91592254-13ED-5501-8315-AB4D29BAFF11; **Taxon:** scientificName: *Coelophthiniacurta*; order: Diptera; family: Mycetophilidae; genus: Coelophthinia; specificEpithet: *curta*; scientificNameAuthorship: (Johannsen); **Location:** country: United States; countryCode: USA; stateProvince: California; county: Sonoma; locality: Annadel State Park; verbatimLocality: ravine nr. Warren Richardson trail; verbatimElevation: 220m; verbatimCoordinateSystem: decimal degrees; decimalLatitude: 38.4351; decimalLongitude: -122.6111; **Identification:** identificationID: 13M168; identifiedBy: Peter H. Kerr; dateIdentified: 04/04/2013; **Event:** eventID: CSCA10L011; samplingProtocol: Malaise trap (6m); eventDate: 16.iii–5.v.2010; **Record Level:** type: Preserved Specimen; collectionID: urn:lsid:biocol.org:col:33177; institutionCode: CSCA; basisOfRecord: PreservedSpecimen
- **Type status:** Other material. **Occurrence:** catalogNumber: 13M648; recordedBy: Peter H. Kerr; individualCount: 1; sex: male; lifeStage: Adult; occurrenceID: F08D8E49-58E1-540B-9CE3-E72BC50EEF8B; **Taxon:** scientificName: *Coelophthiniacurta*; order: Diptera; family: Mycetophilidae; genus: Coelophthinia; specificEpithet: *curta*; scientificNameAuthorship: (Johannsen); **Location:** country: United States; countryCode: USA; stateProvince: California; county: Sonoma; locality: Annadel State Park; verbatimLocality: ravine nr. Warren Richardson trail; verbatimElevation: 220m; verbatimCoordinateSystem: decimal degrees; decimalLatitude: 38.4351; decimalLongitude: -122.6111; **Identification:** identificationID: 13M648; identifiedBy: Peter H. Kerr; dateIdentified: 10/08/2013; **Event:** eventID: CSCA10L011; samplingProtocol: Malaise trap (6m); eventDate: 16.iii–5.v.2010; **Record Level:** type: Preserved Specimen; collectionID: urn:lsid:biocol.org:col:33178; institutionCode: CSCA; basisOfRecord: PreservedSpecimen
- **Type status:** Other material. **Occurrence:** catalogNumber: 20i322; recordedBy: Peter H. Kerr; individualCount: 1; sex: male; lifeStage: Adult; occurrenceID: 45F32441-C235-5ACA-B298-F2AC1E8AD4A1; **Taxon:** scientificName: *Coelophthiniacurta*; order: Diptera; family: Mycetophilidae; genus: Coelophthinia; specificEpithet: *curta*; scientificNameAuthorship: (Johannsen); **Location:** country: United States; countryCode: USA; stateProvince: California; county: Sonoma; locality: Annadel State Park; verbatimLocality: ravine nr. Warren Richardson trail; verbatimElevation: 220m; verbatimCoordinateSystem: decimal degrees; decimalLatitude: 38.4351; decimalLongitude: -122.6111; **Identification:** identificationID: 20i322; identifiedBy: Peter H. Kerr; dateIdentified: 09/30/2020; **Event:** eventID: CSCA10L011; samplingProtocol: Malaise trap (6m); eventDate: 16.iii–5.v.2010; **Record Level:** type: Preserved Specimen; collectionID: urn:lsid:biocol.org:col:33182; institutionCode: CSCA; basisOfRecord: PreservedSpecimen
- **Type status:** Other material. **Occurrence:** catalogNumber: 14P293; recordedBy: Peter H. Kerr; individualCount: 1; sex: male; lifeStage: Adult; occurrenceID: D214BD80-73AC-53D4-BE2D-3BFB7508345C; **Taxon:** scientificName: *Coelophthiniacurta*; order: Diptera; family: Mycetophilidae; genus: Coelophthinia; specificEpithet: *curta*; scientificNameAuthorship: (Johannsen); **Location:** country: United States; countryCode: USA; stateProvince: California; county: Tulare; locality: Whitaker Forest, E. Eshom Cr. Drainage; verbatimLocality: nr. tree #142; verbatimElevation: 1650m; verbatimCoordinateSystem: decimal degrees; decimalLatitude: 36.7062; decimalLongitude: -118.9319; **Identification:** identificationID: 14P293; identifiedBy: Peter H. Kerr; dateIdentified: 02/25/2014; **Event:** eventID: CSCA10L174; samplingProtocol: Malaise trap (6m); eventDate: 3.vi–16.vii.2010; **Record Level:** type: Preserved Specimen; collectionID: urn:lsid:biocol.org:col:33179; institutionCode: CSCA; basisOfRecord: PreservedSpecimen
- **Type status:** Other material. **Occurrence:** catalogNumber: 20i431; recordedBy: Peter H. Kerr; individualCount: 1; sex: male; lifeStage: Adult; occurrenceID: 41D15E30-95AC-5168-9042-D4CFDBB48F6F; **Taxon:** scientificName: *Coelophthiniacurta*; order: Diptera; family: Mycetophilidae; genus: Coelophthinia; infraspecificEpithet: *curta*; scientificNameAuthorship: (Johannsen); **Location:** country: United States; countryCode: USA; stateProvince: California; county: Tulare; locality: Whitaker Forest, E. Eshom Cr. Drainage; verbatimLocality: nr. tree #142; verbatimElevation: 1650m; verbatimCoordinateSystem: decimal degrees; decimalLatitude: 36.7062; decimalLongitude: -118.9319; **Identification:** identificationID: 20i431; identifiedBy: Peter H. Kerr; dateIdentified: 10/20/2020; **Event:** eventID: CSCA10L174; samplingProtocol: Malaise trap (6m); eventDate: 3.vi–16.vii.2010; **Record Level:** type: Preserved Specimen; collectionID: urn:lsid:biocol.org:col:33186; institutionCode: CSCA; basisOfRecord: PreservedSpecimen
- **Type status:** Other material. **Occurrence:** catalogNumber: 21K695; recordedBy: Peter H. Kerr; individualCount: 2; sex: male; lifeStage: Adult; occurrenceID: 9CC8DAEB-540E-582F-ABB8-99685FC4F2C0; **Taxon:** scientificName: *Coelophthiniacurta*; order: Diptera; family: Mycetophilidae; genus: Coelophthinia; specificEpithet: *curta*; scientificNameAuthorship: (Johannsen); **Location:** country: United States; countryCode: USA; stateProvince: California; county: Tulare; locality: Whitaker Forest, E. Eshom Cr. Drainage; verbatimLocality: nr. tree #142; verbatimElevation: 1650m; verbatimCoordinateSystem: decimal degrees; decimalLatitude: 36.7062; decimalLongitude: -118.9319; **Identification:** identificationID: 21K695; identifiedBy: Peter H. Kerr; dateIdentified: 02/04/2021; **Event:** eventID: CSCA10L174; samplingProtocol: Malaise trap (6m); eventDate: 3.vi–16.vii.2010; **Record Level:** type: Preserved Specimen; collectionID: urn:lsid:biocol.org:col:33190; institutionCode: CSCA; basisOfRecord: PreservedSpecimen
- **Type status:** Other material. **Occurrence:** catalogNumber: 21K696; recordedBy: Peter H. Kerr; individualCount: 1; sex: female; lifeStage: Adult; occurrenceID: D5286190-FFA3-53D5-9E99-EB437BCCC5C0; **Taxon:** scientificName: *Coelophthiniacurta*; order: Diptera; family: Mycetophilidae; genus: Coelophthinia; specificEpithet: *curta*; scientificNameAuthorship: (Johannsen); **Location:** country: United States; countryCode: USA; stateProvince: California; county: Tulare; locality: Whitaker Forest, E. Eshom Cr. Drainage; verbatimLocality: nr. tree #142; verbatimElevation: 1650m; verbatimCoordinateSystem: decimal degrees; decimalLatitude: 36.7062; decimalLongitude: -118.9319; **Identification:** identificationID: 21K696; identifiedBy: Peter H. Kerr; dateIdentified: 02/04/2021; **Event:** eventID: CSCA10L174; samplingProtocol: Malaise trap (6m); eventDate: 3.vi–16.vii.2010; **Record Level:** type: Preserved Specimen; collectionID: urn:lsid:biocol.org:col:33191; institutionCode: CSCA; basisOfRecord: PreservedSpecimen
- **Type status:** Other material. **Occurrence:** catalogNumber: 22R074; recordedBy: Peter H. Kerr; individualCount: 1; sex: male; lifeStage: Adult; occurrenceID: F8A6451D-F7BF-5099-AA37-B7C6EF2BA3A2; **Taxon:** scientificName: *Coelophthiniacurta*; order: Diptera; family: Mycetophilidae; genus: Coelophthinia; specificEpithet: *curta*; scientificNameAuthorship: (Johannsen); **Location:** country: United States; countryCode: USA; stateProvince: California; county: Tulare; locality: Whitaker Forest, E. Eshom Cr. Drainage; verbatimLocality: nr. tree #142; verbatimElevation: 1650m; verbatimCoordinateSystem: decimal degrees; decimalLatitude: 36.7062; decimalLongitude: -118.9319; **Identification:** identificationID: 22R074; identifiedBy: Peter H. Kerr; dateIdentified: 11/22/2022; **Event:** eventID: CSCA10L174; samplingProtocol: Malaise trap (6m); eventDate: 3.vi–16.vii.2010; **Record Level:** type: Preserved Specimen; collectionID: urn:lsid:biocol.org:col:33197; institutionCode: CSCA; basisOfRecord: PreservedSpecimen
- **Type status:** Other material. **Occurrence:** catalogNumber: 11G245; recordedBy: Peter H. Kerr; individualCount: 5; sex: male; lifeStage: Adult; occurrenceID: 10D313D4-F5AC-5E90-9901-AC90C44AFFDD; **Taxon:** scientificName: *Coelophthiniacurta*; order: Diptera; family: Mycetophilidae; genus: Coelophthinia; specificEpithet: *curta*; scientificNameAuthorship: (Johannsen); **Location:** country: United States; countryCode: USA; stateProvince: California; county: Tulare; locality: Whitaker Forest, E. Eshom Cr. Drainage; verbatimLocality: nr. tree #142; verbatimElevation: 1650m; verbatimCoordinateSystem: decimal degrees; decimalLatitude: 36.7062; decimalLongitude: -118.9319; **Identification:** identificationID: 11G245; identifiedBy: Peter H. Kerr; dateIdentified: 01/31/2011; **Event:** eventID: CSCA10L258; samplingProtocol: yellow pan trap; eventDate: 3.vi–16.vii.2010; **Record Level:** type: Preserved Specimen; collectionID: urn:lsid:biocol.org:col:33162; institutionCode: CSCA; basisOfRecord: PreservedSpecimen
- **Type status:** Other material. **Occurrence:** catalogNumber: 11G710; recordedBy: Peter H. Kerr; individualCount: 1; sex: male; lifeStage: Adult; occurrenceID: 921E53B5-E4A8-531F-9DBE-183370129244; **Taxon:** scientificName: *Coelophthiniacurta*; order: Diptera; family: Mycetophilidae; genus: Coelophthinia; specificEpithet: *curta*; scientificNameAuthorship: (Johannsen); **Location:** country: United States; countryCode: USA; stateProvince: California; county: Tulare; locality: Whitaker Forest, E. Eshom Cr. Drainage; verbatimLocality: nr. tree #142; verbatimElevation: 1650m; verbatimCoordinateSystem: decimal degrees; decimalLatitude: 36.7062; decimalLongitude: -118.9319; **Identification:** identificationID: 11G710; identifiedBy: Peter H. Kerr; dateIdentified: 06/01/2011; **Event:** eventID: CSCA10L258; samplingProtocol: yellow pan trap; eventDate: 3.vi–16.vii.2010; **Record Level:** type: Preserved Specimen; collectionID: urn:lsid:biocol.org:col:33166; institutionCode: CSCA; basisOfRecord: PreservedSpecimen
- **Type status:** Other material. **Occurrence:** catalogNumber: 20i323; recordedBy: Peter H. Kerr; individualCount: 1; sex: male; lifeStage: Adult; occurrenceID: 7636D39C-8B47-59C0-A72B-36AC8BB7BC6F; **Taxon:** scientificName: *Coelophthiniacurta*; order: Diptera; family: Mycetophilidae; genus: Coelophthinia; specificEpithet: *curta*; scientificNameAuthorship: (Johannsen); **Location:** country: United States; countryCode: USA; stateProvince: California; county: Tulare; locality: Whitaker Forest, E. Eshom Cr. Drainage; verbatimLocality: nr. tree #142; verbatimElevation: 1650m; verbatimCoordinateSystem: decimal degrees; decimalLatitude: 36.7062; decimalLongitude: -118.9319; **Identification:** identificationID: 20i323; identifiedBy: Peter H. Kerr; dateIdentified: 09/30/2020; **Event:** eventID: CSCA10L258; samplingProtocol: yellow pan trap; eventDate: 3.vi–16.vii.2010; **Record Level:** type: Preserved Specimen; collectionID: urn:lsid:biocol.org:col:33183; institutionCode: CSCA; basisOfRecord: PreservedSpecimen
- **Type status:** Other material. **Occurrence:** catalogNumber: 11G228; recordedBy: Peter H. Kerr; individualCount: 1; sex: male; lifeStage: Adult; occurrenceID: 3F6E815B-F5FF-5BB0-B4BB-6B3A0F74118D; **Taxon:** scientificName: *Coelophthiniacurta*; order: Diptera; family: Mycetophilidae; genus: Coelophthinia; specificEpithet: *curta*; scientificNameAuthorship: (Johannsen); **Location:** country: United States; countryCode: USA; stateProvince: California; county: Tulare; locality: Whitaker Forest, E. Eshom Cr. Drainage; verbatimLocality: nr. tree #142; verbatimElevation: 1650m; verbatimCoordinateSystem: decimal degrees; decimalLatitude: 36.7062; decimalLongitude: -118.9319; **Identification:** identificationID: 11G228; identifiedBy: Peter H. Kerr; dateIdentified: 01/31/2011; **Event:** eventID: CSCA10L286; samplingProtocol: Malaise trap (6m); eventDate: 16.vii–12.viii.2010; **Record Level:** type: Preserved Specimen; collectionID: urn:lsid:biocol.org:col:33161; institutionCode: CSCA; basisOfRecord: PreservedSpecimen
- **Type status:** Other material. **Occurrence:** catalogNumber: 12J295; recordedBy: Peter H. Kerr; individualCount: 1; sex: male; lifeStage: Adult; occurrenceID: 4838DE00-CD18-57DD-A0E5-E64BDC07BA97; **Taxon:** scientificName: *Coelophthiniacurta*; order: Diptera; family: Mycetophilidae; genus: Coelophthinia; specificEpithet: *curta*; scientificNameAuthorship: (Johannsen); **Location:** country: United States; countryCode: USA; stateProvince: California; county: Marin; locality: Pt. Reyes National Seashore; verbatimLocality: Limantour Rd., near Sky trailhead; verbatimElevation: 220m; verbatimCoordinateSystem: decimal degrees; decimalLatitude: 38.05259; decimalLongitude: -122.8263; **Identification:** identificationID: 12J295; identifiedBy: Peter H. Kerr; dateIdentified: 05/08/2012; **Event:** eventID: CSCA12L022; samplingProtocol: Malaise trap (2m); eventDate: 13.iii–1.v.2012; **Record Level:** type: Preserved Specimen; collectionID: urn:lsid:biocol.org:col:33171; institutionCode: CSCA; basisOfRecord: PreservedSpecimen
- **Type status:** Other material. **Occurrence:** catalogNumber: 12J435; recordedBy: Peter H. Kerr; individualCount: 1; sex: male; lifeStage: Adult; occurrenceID: 396FDA04-2F3D-5225-8D39-450A809B76C4; **Taxon:** scientificName: *Coelophthiniacurta*; order: Diptera; family: Mycetophilidae; genus: Coelophthinia; specificEpithet: *curta*; scientificNameAuthorship: (Johannsen); **Location:** country: United States; countryCode: USA; stateProvince: California; county: Marin; locality: Pt. Reyes National Seashore; verbatimLocality: Mt. Vision Rd., 1.8mi E SFDrakeBlvd; verbatimElevation: 280m; verbatimCoordinateSystem: decimal degrees; decimalLatitude: 38.10134; decimalLongitude: -122.8877; **Identification:** identificationID: 12J435; identifiedBy: Peter H. Kerr; dateIdentified: 05/17/2012; **Event:** eventID: CSCA12L023; samplingProtocol: Malaise trap (6m); eventDate: 13.iii–1.v.2012; **Record Level:** type: Preserved Specimen; collectionID: urn:lsid:biocol.org:col:33172; institutionCode: CSCA; basisOfRecord: PreservedSpecimen
- **Type status:** Other material. **Occurrence:** catalogNumber: 12J573; recordedBy: Peter H. Kerr; individualCount: 1; sex: male; lifeStage: Adult; occurrenceID: B39BCF55-86B5-5FB7-BD6D-7961DCEA94B3; **Taxon:** scientificName: *Coelophthiniacurta*; order: Diptera; family: Mycetophilidae; genus: Coelophthinia; specificEpithet: *curta*; scientificNameAuthorship: (Johannsen); **Location:** country: United States; countryCode: USA; stateProvince: California; county: Marin; locality: Pt. Reyes National Seashore; verbatimLocality: Mt. Vision Rd., 1.8mi E SFDrakeBlvd; verbatimElevation: 280m; verbatimCoordinateSystem: decimal degrees; decimalLatitude: 38.10134; decimalLongitude: -122.8877; **Identification:** identificationID: 12J573; identifiedBy: Peter H. Kerr; dateIdentified: 06/01/2012; **Event:** eventID: CSCA12L023; samplingProtocol: Malaise trap (6m); eventDate: 13.iii–1.v.2012; **Record Level:** type: Preserved Specimen; collectionID: urn:lsid:biocol.org:col:33173; institutionCode: CSCA; basisOfRecord: PreservedSpecimen
- **Type status:** Other material. **Occurrence:** catalogNumber: 20i325; recordedBy: Peter H. Kerr; individualCount: 1; sex: male; lifeStage: Adult; occurrenceID: BE45D188-FACD-5ACD-9BD5-67E6B0983EE3; **Taxon:** scientificName: *Coelophthiniacurta*; order: Diptera; family: Mycetophilidae; genus: Coelophthinia; specificEpithet: *curta*; scientificNameAuthorship: (Johannsen); **Location:** country: United States; countryCode: USA; stateProvince: California; county: Marin; locality: Pt. Reyes National Seashore; verbatimLocality: Mt. Vision Rd., 1.8mi E SFDrakeBlvd; verbatimElevation: 280m; verbatimCoordinateSystem: decimal degrees; decimalLatitude: 38.10134; decimalLongitude: -122.8877; **Identification:** identificationID: 20i325; identifiedBy: Peter H. Kerr; dateIdentified: 09/30/2020; **Event:** eventID: CSCA12L023; samplingProtocol: Malaise trap (6m); eventDate: 13.iii–1.v.2012; **Record Level:** type: Preserved Specimen; collectionID: urn:lsid:biocol.org:col:33184; institutionCode: CSCA; basisOfRecord: PreservedSpecimen
- **Type status:** Other material. **Occurrence:** catalogNumber: 12K890; recordedBy: Peter H. Kerr; individualCount: 1; sex: male; lifeStage: Adult; occurrenceID: 805AF7B1-DB0C-50FD-A717-DC8D71A102D6; **Taxon:** scientificName: *Coelophthiniacurta*; order: Diptera; family: Mycetophilidae; genus: Coelophthinia; specificEpithet: *curta*; scientificNameAuthorship: (Johannsen); **Location:** country: United States; countryCode: USA; stateProvince: California; county: Marin; locality: Pt. Reyes National Seashore; verbatimLocality: Limantour Rd., near Sky trailhead; verbatimElevation: 220m; verbatimCoordinateSystem: decimal degrees; decimalLatitude: 38.0526; decimalLongitude: -122.8263; **Identification:** identificationID: 12K890; identifiedBy: Peter H. Kerr; dateIdentified: 12/27/2012; **Event:** eventID: CSCA12L256; samplingProtocol: Malaise trap (2m); eventDate: 1.v–4.vii.2012; **Record Level:** type: Preserved Specimen; collectionID: urn:lsid:biocol.org:col:33176; institutionCode: CSCA; basisOfRecord: PreservedSpecimen
- **Type status:** Other material. **Occurrence:** catalogNumber: 12K408; recordedBy: Peter H. Kerr; individualCount: 1; sex: male; lifeStage: Adult; occurrenceID: E60DCE64-547D-5E73-B9AB-C61B95CD9582; **Taxon:** scientificName: *Coelophthiniacurta*; order: Diptera; family: Mycetophilidae; genus: Coelophthinia; specificEpithet: *curta*; scientificNameAuthorship: (Johannsen); **Location:** country: United States; countryCode: USA; stateProvince: California; county: San Bernardino; locality: SBNF: Glass Rd.; verbatimCoordinateSystem: decimal degrees; decimalLatitude: 34.1744; decimalLongitude: -116.8971; **Identification:** identificationID: 12K408; identifiedBy: Peter H. Kerr; dateIdentified: 11/21/2022; **Event:** eventID: CSCA12L344; samplingProtocol: FIT; eventDate: 13–20.v.2006; **Record Level:** type: Preserved Specimen; collectionID: urn:lsid:biocol.org:col:33174; institutionCode: CSCA; basisOfRecord: PreservedSpecimen
- **Type status:** Other material. **Occurrence:** catalogNumber: 20i321; recordedBy: Peter H. Kerr; individualCount: 1; sex: male; lifeStage: Adult; occurrenceID: 9158CA77-4415-5D36-9C64-DBB1470E98F7; **Taxon:** scientificName: *Coelophthiniacurta*; order: Diptera; family: Mycetophilidae; genus: Coelophthinia; specificEpithet: *curta*; scientificNameAuthorship: (Johannsen); **Location:** country: United States; countryCode: USA; stateProvince: California; county: San Bernardino; locality: SBNF: Glass Rd.; verbatimCoordinateSystem: decimal degrees; decimalLatitude: 34.1744; decimalLongitude: -116.8971; **Identification:** identificationID: 20i321; identifiedBy: Peter H. Kerr; dateIdentified: 09/30/2020; **Event:** eventID: CSCA12L344; samplingProtocol: FIT; eventDate: 13–20.v.2006; **Record Level:** type: Preserved Specimen; collectionID: urn:lsid:biocol.org:col:33181; institutionCode: CSCA; basisOfRecord: PreservedSpecimen
- **Type status:** Other material. **Occurrence:** catalogNumber: 20i430; recordedBy: Peter H. Kerr; individualCount: 1; sex: male; lifeStage: Adult; occurrenceID: DFB16E4B-F06D-56A0-AF03-E2645B1C4654; **Taxon:** scientificName: *Coelophthiniacurta*; order: Diptera; family: Mycetophilidae; genus: Coelophthinia; specificEpithet: *curta*; scientificNameAuthorship: (Johannsen); **Location:** country: United States; countryCode: USA; stateProvince: California; county: San Bernardino; locality: SBNF: Glass Rd.; verbatimCoordinateSystem: decimal degrees; decimalLatitude: 34.1744; decimalLongitude: -116.8971; **Identification:** identificationID: 20i430; identifiedBy: Peter H. Kerr; dateIdentified: 10/20/2020; **Event:** eventID: CSCA12L344; samplingProtocol: FIT; eventDate: 13–20.v.2006; **Record Level:** type: Preserved Specimen; collectionID: urn:lsid:biocol.org:col:33185; institutionCode: CSCA; basisOfRecord: PreservedSpecimen
- **Type status:** Other material. **Occurrence:** catalogNumber: 17X552; recordedBy: Peter H. Kerr; individualCount: 1; sex: male; lifeStage: Adult; occurrenceID: 176AE54E-B28A-545F-86BD-8EBE3E685D52; **Taxon:** scientificName: *Coelophthiniacurta*; order: Diptera; family: Mycetophilidae; genus: Coelophthinia; specificEpithet: *curta*; scientificNameAuthorship: (Johannsen); **Location:** country: United States; countryCode: USA; stateProvince: California; county: Santa Clara; locality: Wildegarten; verbatimElevation: 370m; verbatimCoordinateSystem: decimal degrees; decimalLatitude: 37.1007; decimalLongitude: -121.9922; **Identification:** identificationID: 17X552; identifiedBy: Peter H. Kerr; dateIdentified: 08/08/2017; **Event:** eventID: CSCA17L418; samplingProtocol: Malaise trap (6m); eventDate: 13.iv–21.v.2017; **Record Level:** type: Preserved Specimen; collectionID: urn:lsid:biocol.org:col:33180; institutionCode: CSCA; basisOfRecord: PreservedSpecimen
- **Type status:** Other material. **Occurrence:** catalogNumber: 08TTML-1780; recordedBy: Peter H. Kerr; individualCount: 1; sex: male; lifeStage: Adult; occurrenceID: A6940C92-DD05-5EF4-9152-DD067C3425BE; **Taxon:** scientificName: *Coelophthiniacurta*; order: Diptera; family: Mycetophilidae; genus: Coelophthinia; specificEpithet: *curta*; scientificNameAuthorship: (Johannsen); **Location:** country: Canada; countryCode: CAN; stateProvince: Ontario; locality: Puslinch; verbatimLocality: Property of Bob Hanner; verbatimElevation: 335m; verbatimCoordinateSystem: decimal degrees; decimalLatitude: 43.4464; decimalLongitude: -80.2512; **Identification:** identificationID: 08TTML-1780; identifiedBy: Peter H. Kerr; dateIdentified: 04/15/2021; **Event:** eventID: L#TT08-09-11c; samplingProtocol: by hand; eventDate: 2008-09-11; **Record Level:** type: Preserved Specimen; collectionID: urn:lsid:biocol.org:col:33194; institutionCode: BIOUG; basisOfRecord: PreservedSpecimen

#### Description

Male (Fig. 12)

Coloration and general body characteristics as in the genus description. Body length 2.8–3.7 mm. Wing length 2.5–3.0 mm; ratio of length to width 2.8. Sensory organ dorsally on the basal half of mid-tibia elongate oval, 10 times longer than wide, length 0.3 times that of tibial length.

Terminalia. Tergite 9 nearly quadrate in dorsal view, slightly longer than wide, curved like a hood in lateral view. Medial protrusion of tergite 10 short, height approximately 3-4x width in posterior view, densely setose. Gonocoxites in lateral view with a rounded outline, with approximately straight ventral margin. Posterolateral lobe of gonocoxite short and wide, constricted section only approximately 2x apical width, apex more smoothly rounded ventrally, laterally bare for about 1.5x apical width. Spathulate gonocoxal lobe about five times as long as wide, with slightly constricted base, basad of base with 3 long setae not arranged in single line. Dorsal branch of gonostylus small, simple, oblong and semicircular, dorsally setose with some extra long setae. The broad lobe 1 fan-shaped, carrying 7–8 blunt-tipped setae along rim and 4 normal setae subapically on inner side. The narrow, acute tipped lobe 2 with a tiny, practically imperceptible seta subapically; with strong, sharp-tipped seta at approximately its length from the apex of the lobe, followed by one fine seta of similar length and one longer seta in mid-section. Aedeagus 1.2x longer than spathulate gonocoxal lobe in lateral view, with rounded cutout at base of long apical down-curved hook, hook approx. 0.5x length of rest of aedeagus; with a short, inconspicuous sessile projection at the outer side below the curving point of hook.

#### Diagnosis

*Coelophthiniacurta* can be distinguished from other species of the genus by the combination of the following features: Posterolateral lobe of gonocoxite situated along straight ventral margin, broad, nearly as broad as constricted length; spathulate lobe on ventral medial margin of gonocoxite short oblong, mid-tibial organ clearly much longer than width of tibia.

### Coelophthinia cirra

Kerr sp. nov.

EB086745-4440-576B-9ACC-2A3AABCA5E09

10ADBEF3-E2C3-412D-8192-8C2322035BC9

#### Materials

- **Type status:** Holotype. **Occurrence:** catalogNumber: 12J162; recordedBy: Peter H. Kerr; individualCount: 1; sex: male; lifeStage: adult; occurrenceID: 7D202C2D-CD98-5304-B629-4F12A82473A2; **Taxon:** scientificName: *Coelophthiniacirra*; order: Diptera; family: Mycetophilidae; genus: Coelophthinia; specificEpithet: *cirra*; scientificNameAuthorship: Kerr; **Location:** country: United States; countryCode: USA; stateProvince: California; county: Alpine; locality: Grover Hot Springs State Park, nr. Hoffman house; verbatimLocality: Grover Hot Springs State Park; verbatimElevation: 1800m; verbatimCoordinateSystem: decimal degrees; decimalLatitude: 38.6952; decimalLongitude: -119.838; **Identification:** identificationID: 12J162; identifiedBy: Peter H. Kerr; dateIdentified: 6/9/2021; **Event:** eventID: CSCA06LOT476; samplingProtocol: Malaise Trap (2m); eventDate: 14.viii–3.ix.2006; **Record Level:** type: PreservedSpecimen; collectionID: urn:lsid:biocol.org:col:33156; institutionCode: CSCA; basisOfRecord: PreservedSpecimen
- **Type status:** Paratype. **Occurrence:** catalogNumber: 11G757; recordedBy: Peter H. Kerr; individualCount: 1; sex: male; lifeStage: adult; occurrenceID: 7F33526F-B6AA-5F25-BD93-54915F0936EB; **Taxon:** scientificName: *Coelophthiniacirra*; order: Diptera; family: Mycetophilidae; genus: Coelophthinia; specificEpithet: *cirra*; scientificNameAuthorship: Kerr; **Location:** country: United States; countryCode: USA; stateProvince: California; county: Alpine; locality: Grover Hot Springs State Park, forest/meadow edge; verbatimLocality: Grover Hot Springs State Park; verbatimElevation: 1800m; verbatimCoordinateSystem: decimal degrees; decimalLatitude: 38.6996; decimalLongitude: -119.8457; **Identification:** identificationID: 11G757; identifiedBy: Peter H. Kerr; dateIdentified: 6/9/2021; **Event:** eventID: CSCA06LOT275; samplingProtocol: Malaise Trap (2m); eventDate: 11–25.v.2006; **Record Level:** type: PreservedSpecimen; collectionID: urn:lsid:biocol.org:col:33156; institutionCode: CSCA; basisOfRecord: PreservedSpecimen
- **Type status:** Paratype. **Occurrence:** catalogNumber: 21K109; recordedBy: Peter H. Kerr; individualCount: 1; sex: male; lifeStage: adult; occurrenceID: 46672366-0481-5138-BA92-938A553F2952; **Taxon:** scientificName: *Coelophthiniacirra*; order: Diptera; family: Mycetophilidae; genus: Coelophthinia; specificEpithet: *cirra*; scientificNameAuthorship: Kerr; **Location:** country: United States; countryCode: USA; stateProvince: California; county: Alpine; locality: Grover Hot Springs State Park, nr. Hoffman house; verbatimLocality: Grover Hot Springs State Park; verbatimElevation: 1800m; verbatimCoordinateSystem: decimal degrees; decimalLatitude: 38.6952; decimalLongitude: -119.8380; **Identification:** identificationID: 21K10; identifiedBy: Peter H. Kerr; dateIdentified: 6/9/2021; **Event:** eventID: CSCA06LOT275; samplingProtocol: Malaise Trap (2m); eventDate: 11–25.v.2006; **Record Level:** type: PreservedSpecimen; collectionID: urn:lsid:biocol.org:col:33156; institutionCode: CSCA; basisOfRecord: PreservedSpecimen

#### Description

Male. (Fig. 13 Fig. 14)

Coloration and most body characteristics as in the genus description. Body length 2.8 mm. Wing length 2.5 mm; ratio of length to width 2.8. Sensory organ dorsally on the basal half of mid-tibia elongate oval, 3.5 times longer than wide, 0.1 times of tibial length.

Terminalia. Tergite 9 setose, except basal fifth medially, nearly quadrate, slightly longer than wide, medially slightly constricted, basal margin concave, curved like a hood in lateral view. Medial protrusion of tergite 10 a modest bulbous swelling, densely setose. Cerci medially fused except for apical third, setose. Gonocoxites in lateral view with a rounded outline, with straight ventral margin. Posterolateral lobe of gonocoxite long, slender and evenly tapering, about 3-4x longer than wide at apex, rounded apically, laterally bare for about 2x apical width. Gonocoxal lobe spathulate, long and slender, with slightly constricted base, basad of base lacking row of distinctive setae, evenly tapered along dorsoventral margins, external surface bare on apical length approximately 3x apical width, apically rounded. Dorsal branch of gonostylus small, simple, oblong and semicircular, dorsally setose. The broad v br lobe 1 fan-shaped, carrying 6–7 blunt-tipped setae along rim and 4 normal setae on inner side. The narrow, acute tipped v br lobe 2 with a tiny, practically imperceptible seta subapically; with thickened, sharp-tipped seta at approximately its length from the apex of the lobe, followed by one fine seta of similar length and one longer seta near the base of the lobe approximately 1.5x length of v br lobe 2. Aedeagus of same length or slightly longer than spathulate gonocoxal lobe in lateral view, slender throughout, ventral margin entire along length, without rounded cutout at base of hook; with long apical down-curved hook, hook approx. 0.4x length of rest of aedeagus; with a short, inconspicuous sessile projection at the outer side below the curving point of hook.

#### Diagnosis

*Coelophthiniacirra* can be distinguished from other species of the genus by the combination of the following features: mid-tibial organ only slightly longer than width of tibia, gonocoxal lobe situated along straight ventral margin, slender and evenly rounded apically, thinner than constricted length; aedeagus slender throughout, without cutout at base of apical hook.

#### Etymology

Adjective taken from the Latin word *cirrus*, meaning "tendrils" and descriptive of clouds. This species is thin and leggy and found at high elevations, reminiscent of the clouds.

## Identification Keys

### Key to males of Holarctic *Coelophthinia*

**Table:** 

1	Protrusive middle section of tergite 10 long, narrow and curved, elevated well above top of tergite 9 (Figs 7a, 9a).	2
–	Protrusive middle section of tergite 10 short, semicircular, not elevated above top of tergite 9 (eg. Fig. 3a, c).	3
2	Posterolateral lobe of gonocoxite broad, as broad as constricted length; spathulate lobe on ventral medial margin of gonocoxite short oblong; aedeagus short and deeply downcurved (Fig. 9). (Japanese species)	*Coelophthiniaitoae* sp. n.
–	Posterolateral lobe of gonocoxite narrow, much thinner than constricted length; spathulate lobe on ventral medial margin of gonocoxite long with constricted base; aedeagus long and less deeply downcurved (Fig. 7). (Norwegian species)	*Coelophthinialoraasi* sp. n.
3	Posterolateral lobe of gonocoxite situated midway between dorsal and ventral edges, with distinctly convex ventral margin (Figs 5a, 6a). (European species)	4
–	Posterolateral lobe of gonocoxite situated along straight ventral margin (Figs 12a, 13a, 14). (North American species)	5
4	Posterolateral lobe of gonocoxite broad, nearly as broad as constricted length; spathulate lobe on ventral medial margin of gonocoxite short oblong (Fig. 6).	*Coelophthinialata* sp. n.
–	Posterolateral lobe of gonocoxite narrow, much thinner than constricted length; spathulate lobe on ventral medial margin of gonocoxite long with constricted base (Fig. 5).	*Coelophthiniathoracica*
5	Posterolateral lobe of gonocoxite broad, nearly as broad as constricted length; spathulate lobe on ventral medial margin of gonocoxite short oblong (Fig. 12). Mid-tibial organ approx. 10x longer than wide, clearly much longer than width of tibia.	*Coelophthiniacurta*
–	Posterolateral lobe of gonocoxite narrow, much thinner than constricted length; spathulate lobe on ventral medial margin of gonocoxite long with constricted base (Figs 13, 14). Mid-tibial organ 2–3x longer than wide, slightly longer than width of tibia.	*Coelophthiniacirra* sp. n.

## Analysis

### Mitochondrial genome

**Figure 15.**
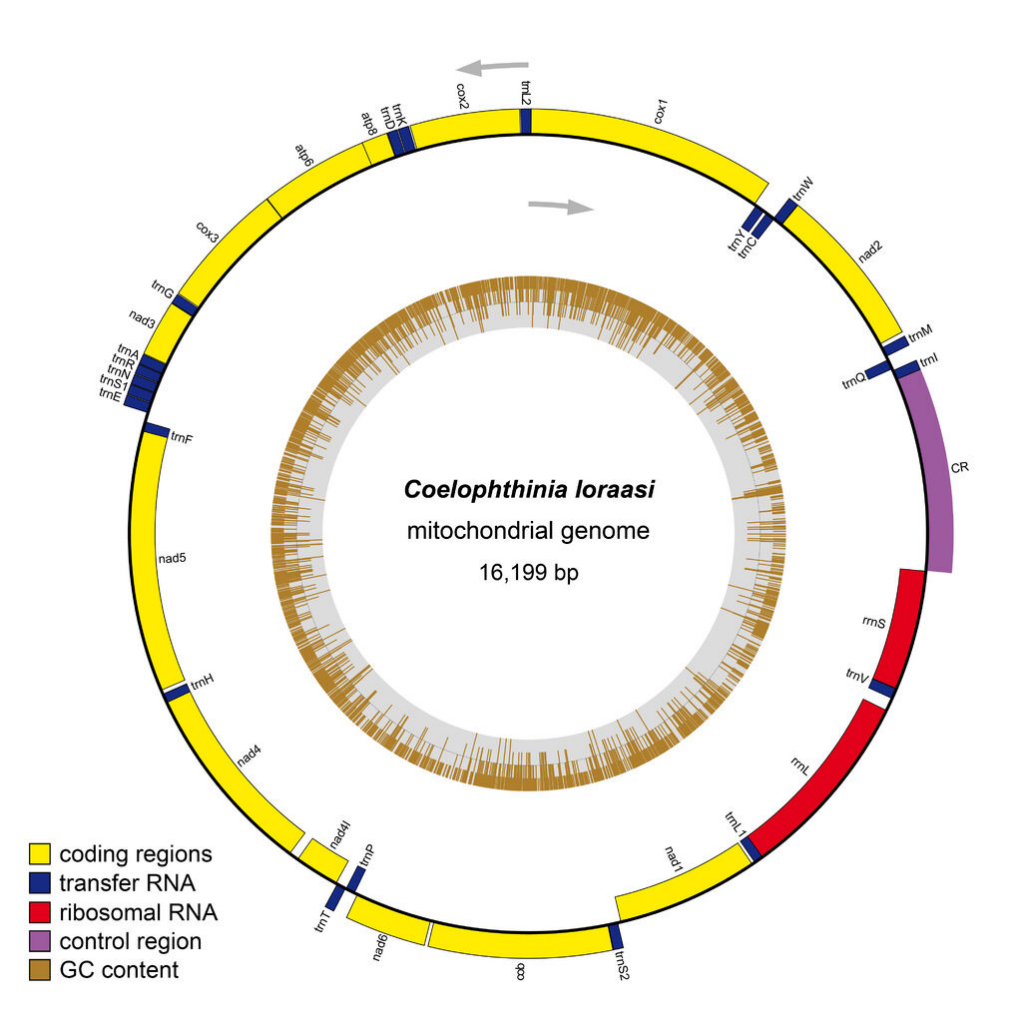
Map of the mitogenome of *Coelophthinialoraasi*. The outer circle displays the genes on the + (outer) and - (inner) strands with coding sequences coloured in yellow, rRNA in red and tRNA in blue. Arrows indicate direction of transcription. The inner circle shows the GC content (brown) as a deviation from the avarage of the total mitogenome.

The mitochondrial genome of *C.loraasi* Kjaerandsen (Fig. 15), comprising 16199 bp, contains the 37 genes (13 Protein, 22 tRNA and two rRNA genes) that are commonly found in animal mitochondria (Boore 1999). The genes are arranged in the same order as the ancestral insect mitochondrial genome (Cameron 2014), as well as the few known mitochondrial genomes of other Mycetophilidae (Wang et al. 2021).

There are ten cases of overlapping genes and 21 cases where intergeneric nucleotides are present (Suppl. material 1). The two largest sequences of intergeneric nucleotides (next to the CR) occur between *rrnL* and *trnV* (81 bp) and between *trnE* and *trnF* (57 bp). The two largest overlaps occur between *trnL1* and *rrnL* (21 bp) and *trnF* and *nad5* (17 bp).

The protein coding genes constitute 11211 bp of the mitochondrial genome. The most frequently encoded amino acids (Suppl. material 2) are Leu, Ser, Ile and Phe and the most frequently used codons are TTA (Leu), ATT (Ile), TTT (Phe) and ATA (Met). Start codons used are ATA, ATC, ATG and ATT, where the most frequently used is ATG. Together the protein coding sequences have A+T content of 74%, AT-skew of -0.1594 and GC-skew of -0,0193, while the entire mitochondrial genome have A+T content of 78.5%, AT-skew of 0.0298 and a GC-skew of -0.2237.

## Discussion

The enormous success of DNA barcoding has accumulated a substantial amount of sequenced insects on BOLD, which is very useful for the kind of new and integrative taxonomic studies that we present here. More than 73,000 specimens belonging to the family Mycetophilidae have been successfully sequenced (Kjærandsen 2022) and, of them, some 11,500 are assigned to the subfamily Gnoristinae. Some 1,300 identified Mycetophilidae species have public barcodes although more than 3,200 different BINs are assigned, this indicating that the majority of the species still remains unidentified beyond the (sub)family level on BOLD. A weakness with the BOLD initiative may be that several of the typically well-funded, large scale DNA barcoding projects, undertaken so far, did not have a focus on, nor adequate resources allocated to, securing high quality morphological identification of the vouchers for the accumulated barcodes. Unfortunately, this critical endeavour of the BOLD archive is largely left to the under-funded and scarce taxonomic expertise to engage in post-sequence work (see Kjærandsen (2022)).

**Figure 16.**
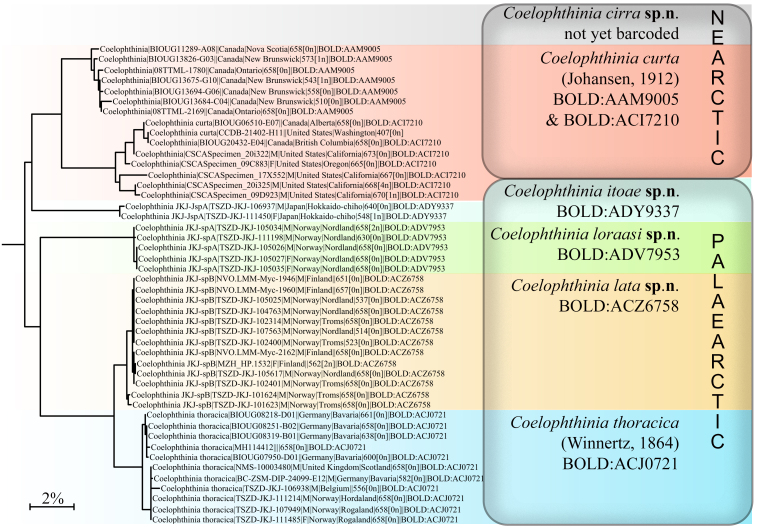
ID-tree (Kimura-2-distance) obtained from BOLD of DNA barcodes of 46 *Coelophthinia* specimens. Species are segregated in coloured boxes and divided by the Palaearctic and Nearctic Regions.

In the Nordic Region, however, strong ties between The Norwegian and Swedish Biodiversity Information Centres, including their Taxonomy Initiatives and NorBOL and FinBOL, are ensuring that the best taxonomic expertise is building up the reference library of the local fauna in the BOLD archive. Hence, the majority of some 15,000 DNA barcoded fungus gnats (Sciaroidea) from the Nordic Region have been identified to species level upon submission and the reference library is profoundly and repeatedly quality-checked and curated after barcodes and BINs are assigned. This has resulted in a high-quality reference library, now covering about 84% of the known Mycetophilidae fauna including more than 150 additional species considered to be new to science (Kjærandsen and Søli 2020, Kjærandsen 2022). An ID-tree search with all private and public sequences of species (10,418 specimens) belonging to the subfamily Gnoristinae on BOLD placed all 46 *Coelophthinia* sequences together in one "monophyletic" clade (Fig. 16), with representatives from *Coelosia*, *Docosia* and *Synapha* as most genetically similar. The maximum genetic spread within the genus is 11.45%. Within species, spread could not be properly calculated as some public sequences have discordant names. Surprisingly, we found that all studied materials of specimens in BINs BOLD:AAM9005 and BOLD:ACI7210 conform with *Coelophthiniacurta*. Yet, *Coelophthiniathoracica* and the new species described here are all confined to a single BIN each. The large genetic spread of *Coelophthiniacurta* with a reciprocal nearest neighbour distance between the two BINs of 3.59% is noteworthy. Taken together with the spit into eastern (BOLD:AAM9005) and western (BOLD:ACI7210) populations, the genetic evidence is, indeed, indicating two cryptic species involved. We refrain, however, from segregating new species based on genetic data only, without any substantial morphological corroboration. This is in rather stark contrast to the clear morphological segregation found among the three Nordic species where the genetic nearest neighbour distance between *Coelophthiniathoracica* and *Coelophthinialata* is only 2%.

The male terminalia in species of *Coelophthinia* are quite similar in general appearance, yet uniquely shaped for the genus. This has probably contributed to the diversity of species previously being overlooked only to be revealed when DNA barcoding started to split them into distinct clades. The morphological differences are, nevertheless, clear and unambiguous when the taxa were re-examined in detail. Morphological differences were just simply overlooked previously due to a convenient lumping of specimens that formed a unique taxon widely different from other species. For this reason, we consider these species pseudocryptic. They were morphologically recognised as distinct only after other methods unveiled their existence. Our concept here is in agreement with Lajus et al. (2015) who stated: “*The large number of cryptic species suggests that the resolution of traditional morphological techniques may be insufficient for taxonomical research. However, some species now considered to be cryptic may, in fact, be designated pseudocryptic after close morphological examination. Thus the “cryptic or pseudocryptic” dilemma speaks to the resolution of morphological analysis and its utility for identifying species*“ .

A 180-degree torsion is always seen where the ventral side of the terminalia is turned to the dorsal side in live specimens (see Fig. 1). The gonostyli of the terminalia are small and retracted within the extended gonocoxites and, unlike in most Mycetophilidae, do not vary considerably between the species, except in the Japanese *Coelophthiniaitoae*. Instead, the outline of the gonocoxites as viewed from the lateral side and the shape of tergite 10 turned out to be reliable characters for separating the species. The female terminalia of the three species with associated females are very similar and no decisive characters were found to segregate them beyond vague differences observed in the few examined specimens. For the time being, DNA-barcoding remains the only safe way to identify females of *Coelophthinia*.

Kaspřák et al. (2019) found genetic support for treating *Coelophthinia* as a sister group to the entire subfamily Mycetophilinae. We find it interesting to note that *Coelophthinia* shares a derived character with all members of the tribe Exechiini, one of two tribes making up the subfamily Mycetophilinae. In *Coelophthinia*, just like in all Exechiini genera, but unlike in all the Mycetophilini genera, the frontal furrow is greatly reduced and only remnants are present as a short, sclerotized line close to the tip of the frontal tubercle and close to the median ocellus. In all other genera of the subfamily Gnoristinae that we have examined, the frontal furrow is complete from the median ocellus to the frontal tubercle.

## Supplementary Material

XML Treatment for Coelophthinia

XML Treatment for Coelophthinia thoracica

XML Treatment for Coelophthinia lata

XML Treatment for Coelophthinia loraasi

XML Treatment for Coelophthinia itoae

XML Treatment for Coelophthinia curta

XML Treatment for Coelophthinia cirra

639F7EE8-F240-5CA3-B032-BFFC0C1BB34C 10.3897/BDJ.11.e98741.suppl1 Supplementary material 1 Mitochondrial gene arrangement Data type genomi Brief description Order of the 37 genes on the Mitochondrial genome of *Coelophthinialoraasi* sp. nov., their affiliation to the forward (+) or reverese (-) strands, start and end positions (begin/end), size in base pairs (size), start codon (start cd) and number of intergeneric nucleotides (inc). A negative intergeneric nucleotide number indicates overlap between the genes. File: oo_584615.pdf https://binary.pensoft.net/file/584615 Jon P. Lindemann

02AFDDD3-CA41-5186-93CD-2E99432D2C46 10.3897/BDJ.11.e98741.suppl2 Supplementary material 2 Codon usage barplot Data type genomic Brief description Barplot of the relative frequencies of codons used in the mitochondrial genome of *C.loraasi* sp. nov., with their encoded amino acids. File: oo_585344.jpg https://binary.pensoft.net/file/585344 Jon P. Lindemann

## Figures and Tables

**Figure 2a.**
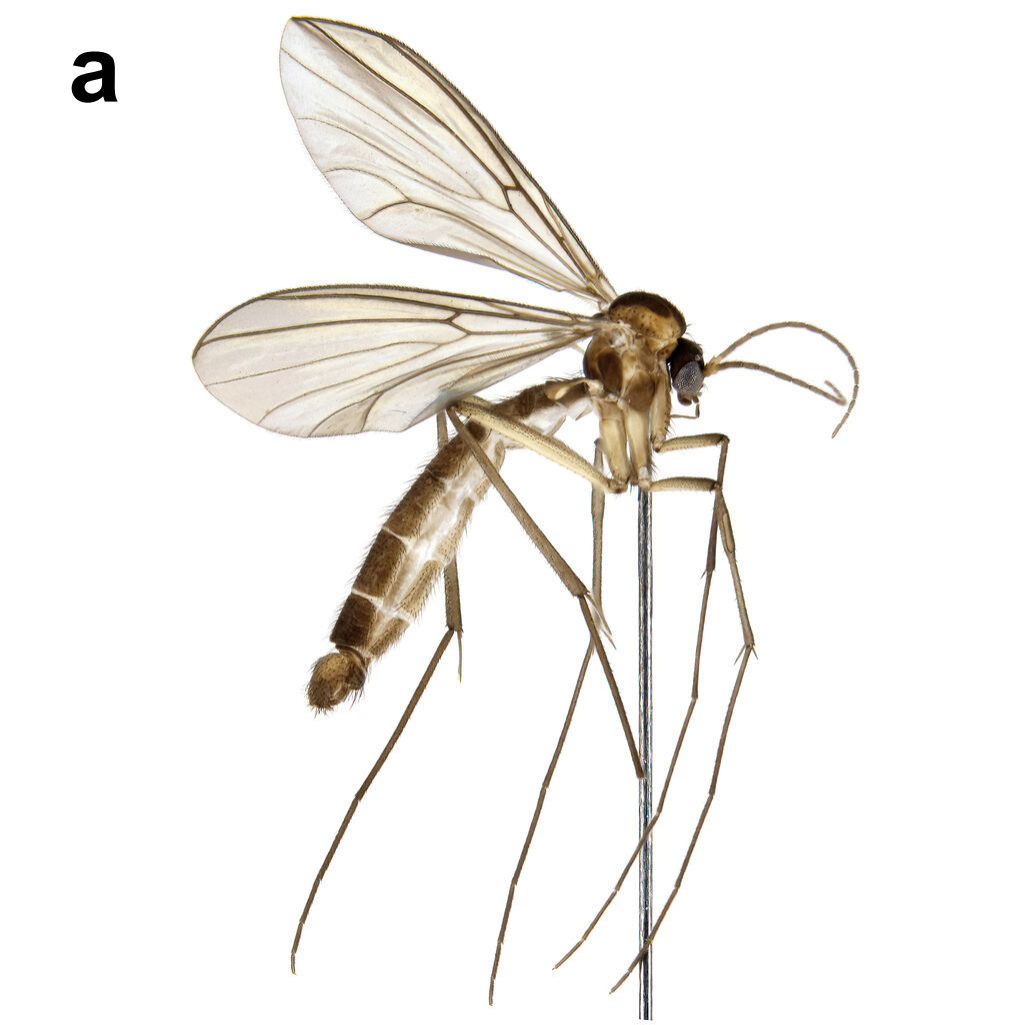
Habitus of HMDS-dried and pinned holotype

**Figure 2b.**
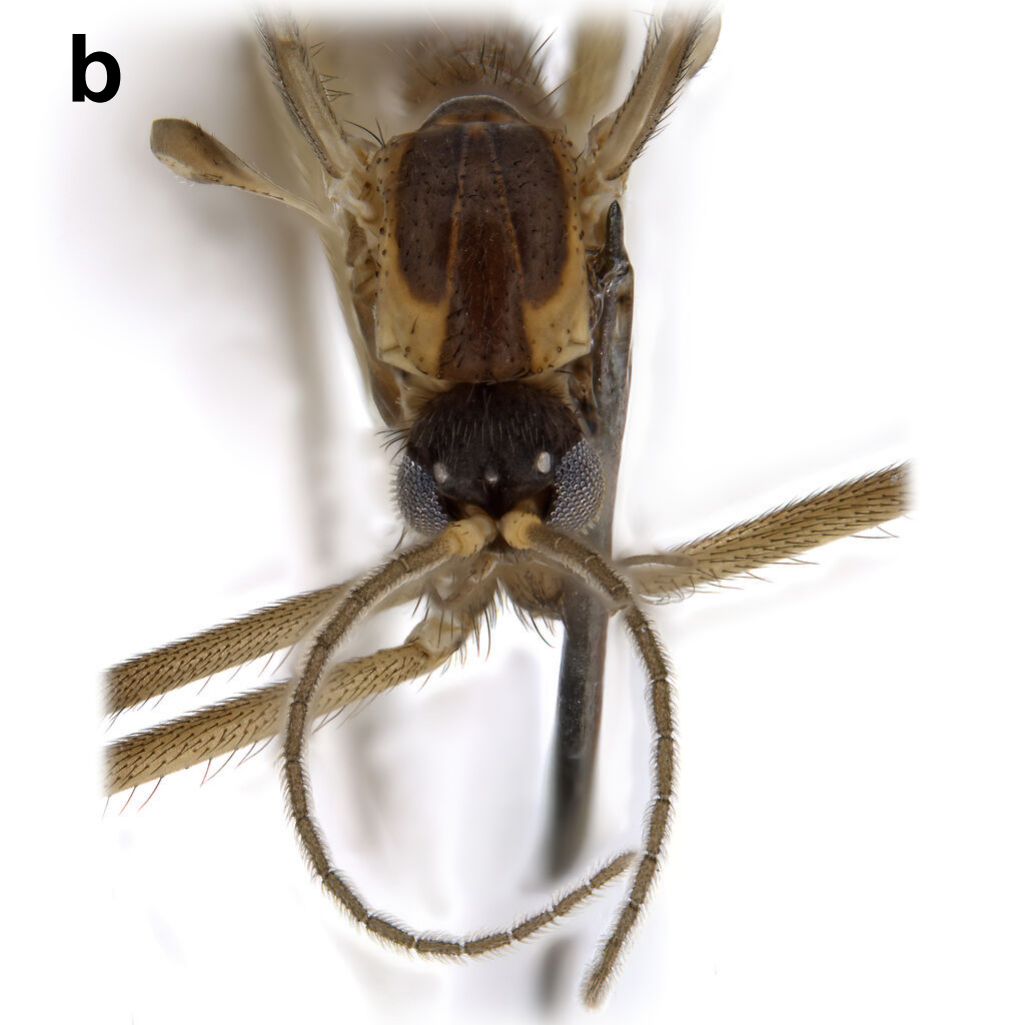
Head and thorax of holotype in dorsal view

**Figure 2c.**
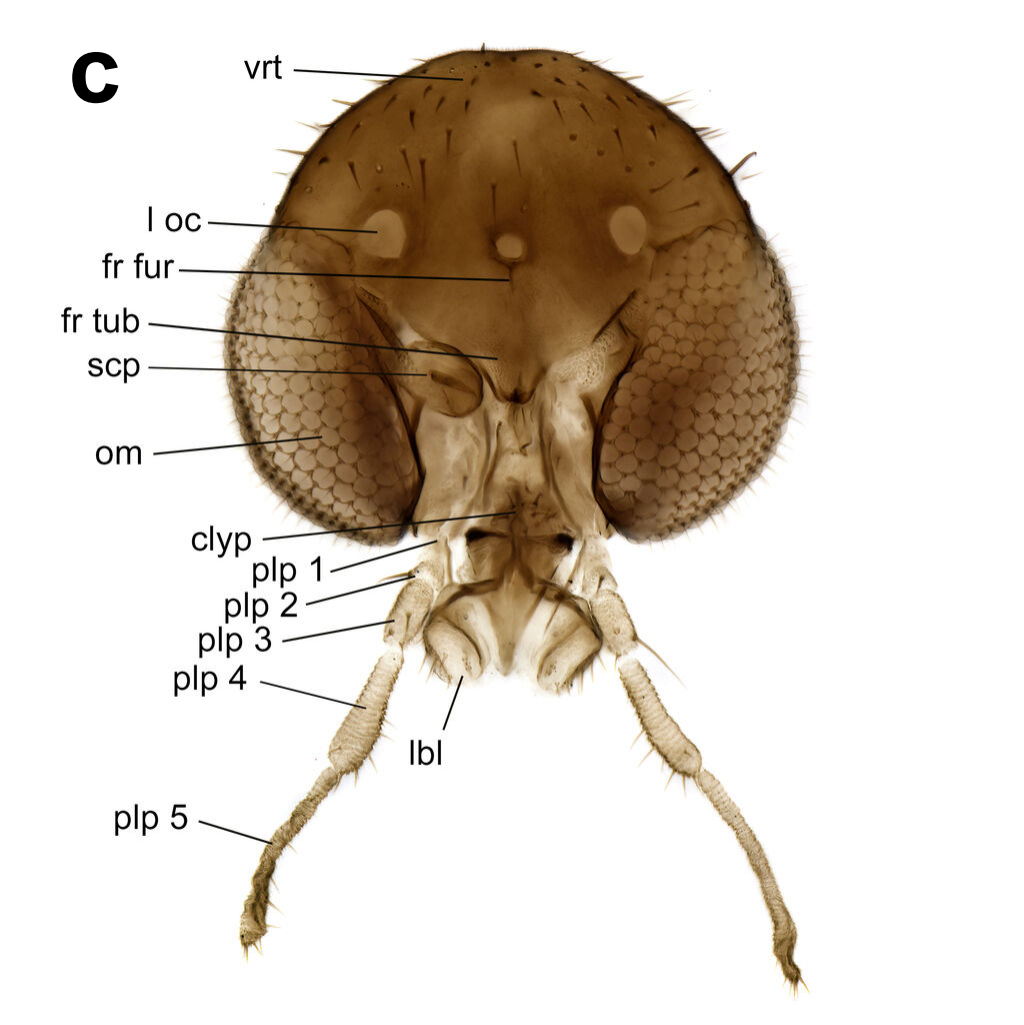
Slide mounted head of paratype (TSZD-JKJ-207664), frontal view

**Figure 2d.**
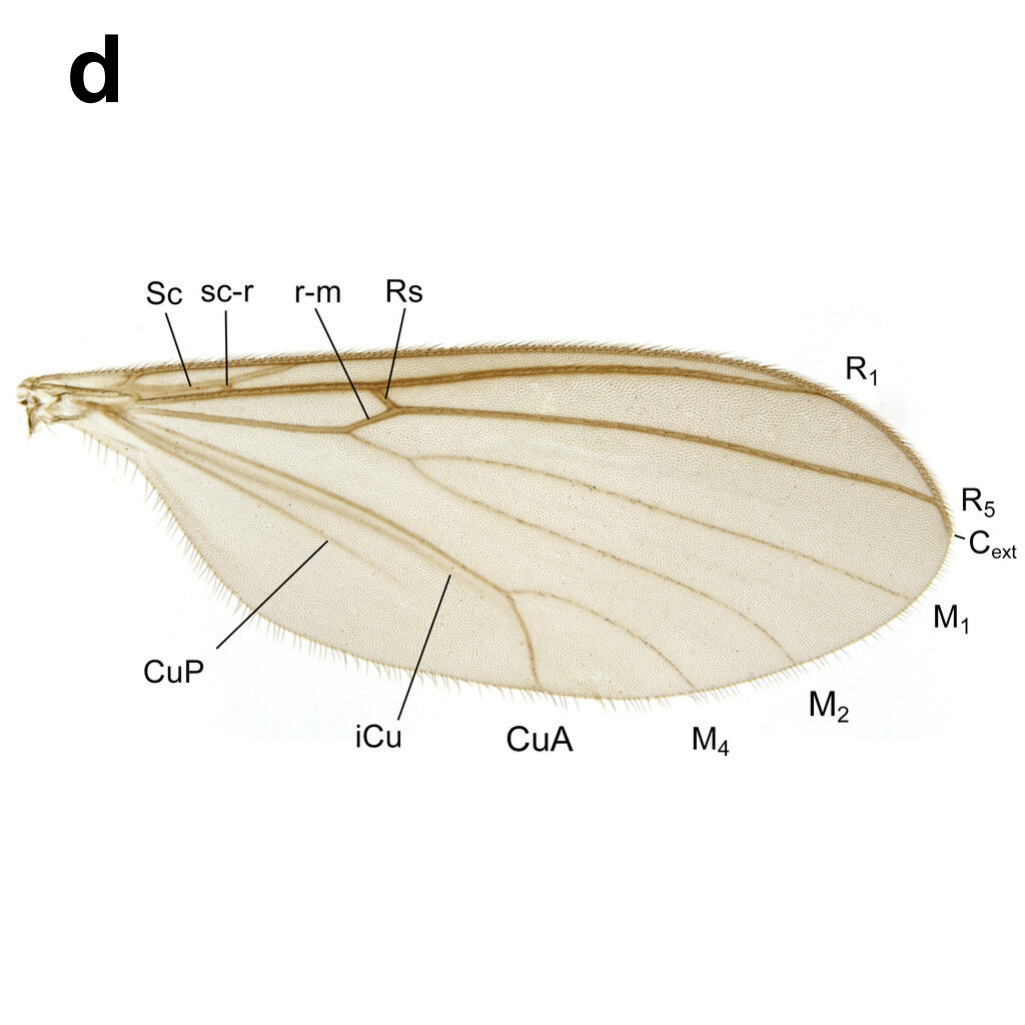
Slide-mounted wing of paratype (TSZD-JKJ-207664)

**Figure 2e.**
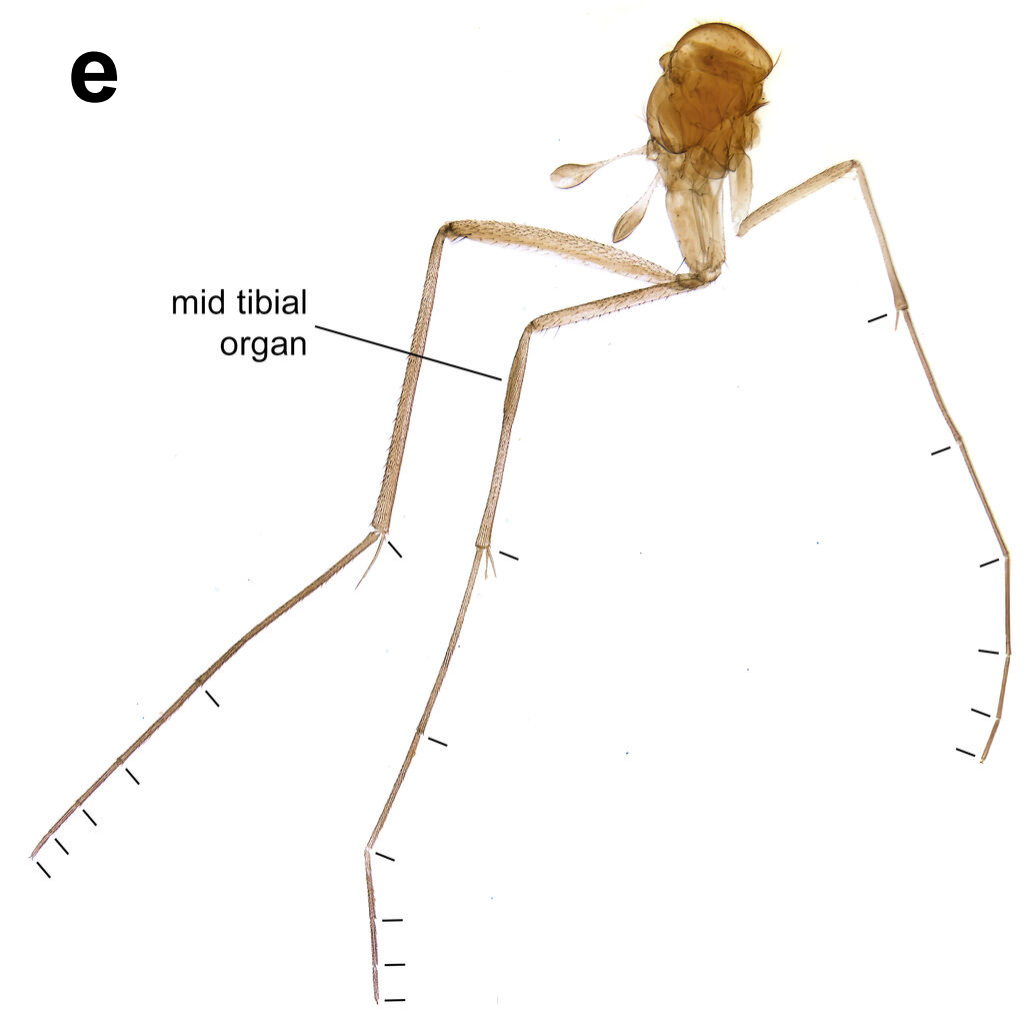
Slide mounted thorax with legs of paratype (TSZD-JKJ-207664). The start and end of each tarsal segment is marked with black pointers for clarity

**Figure 2f.**
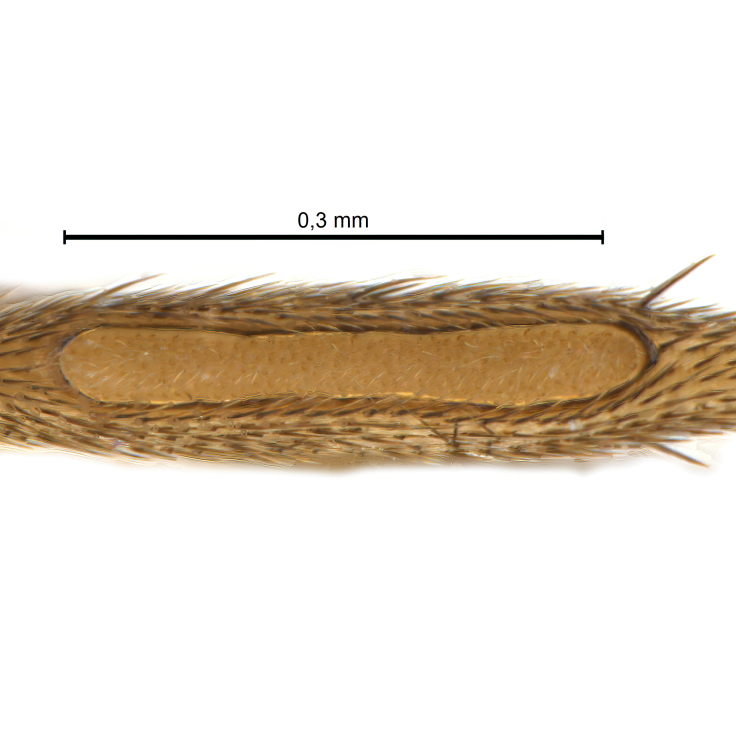
Mid-tibial organ of pinned holotype enlarged and scaled.

**Figure 3a.**
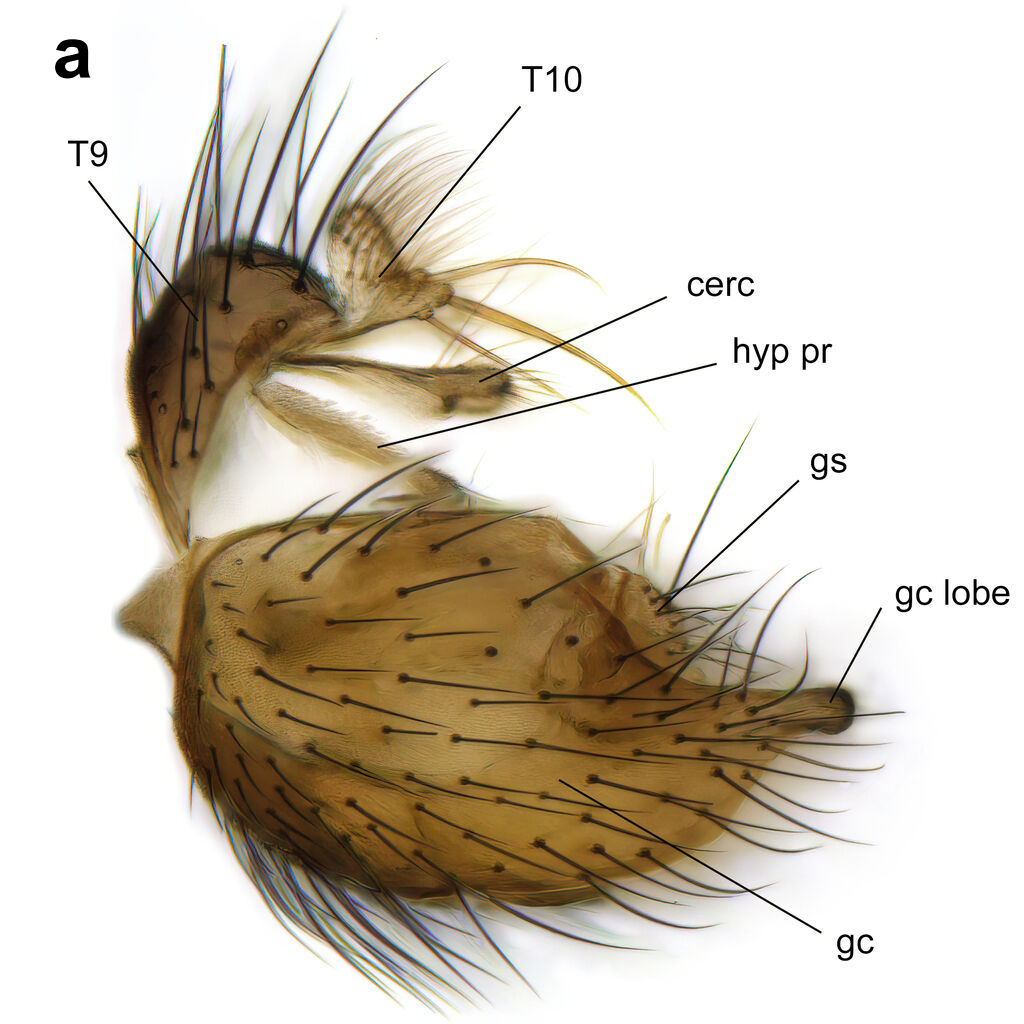
*Coelophthiniathoracica*, male terminalia, lateral view

**Figure 3b.**
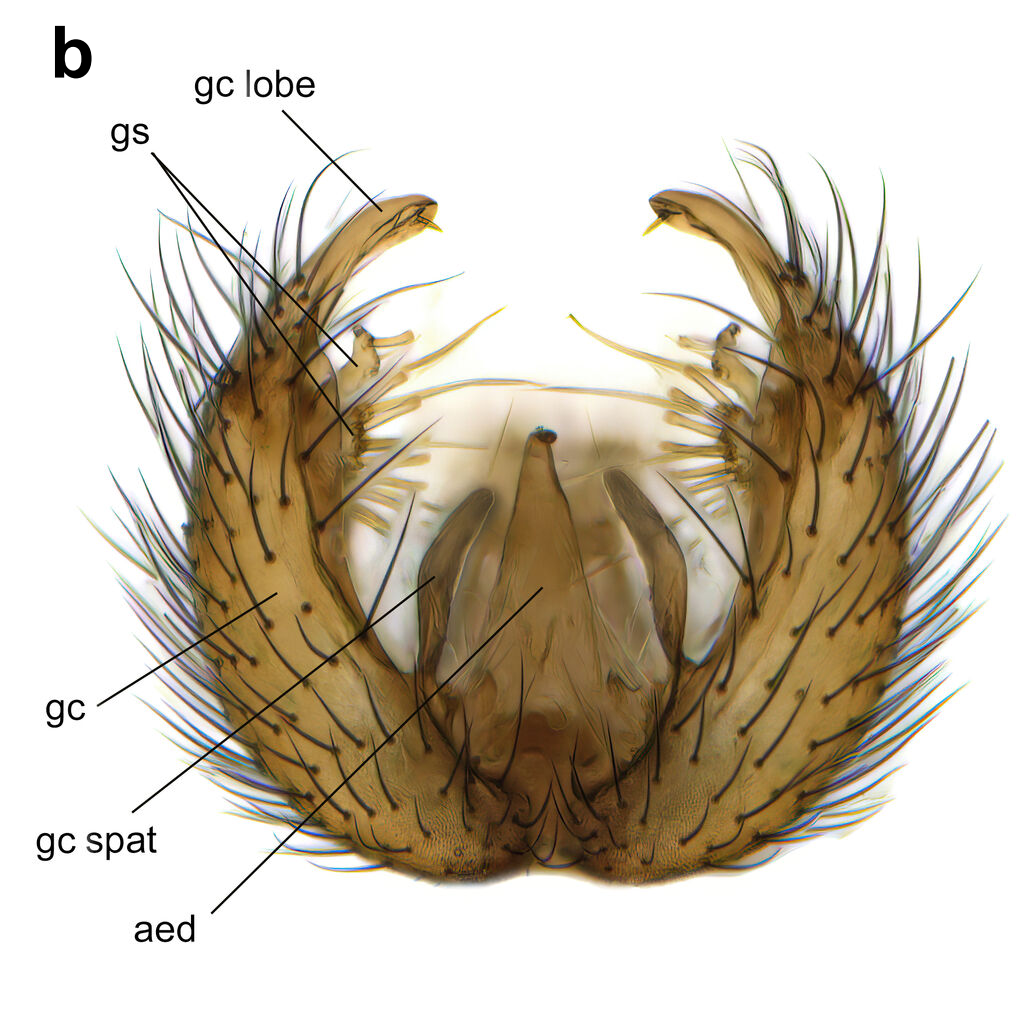
*Coelophthiniathoracica*, male terminalia, ventral view

**Figure 3c.**
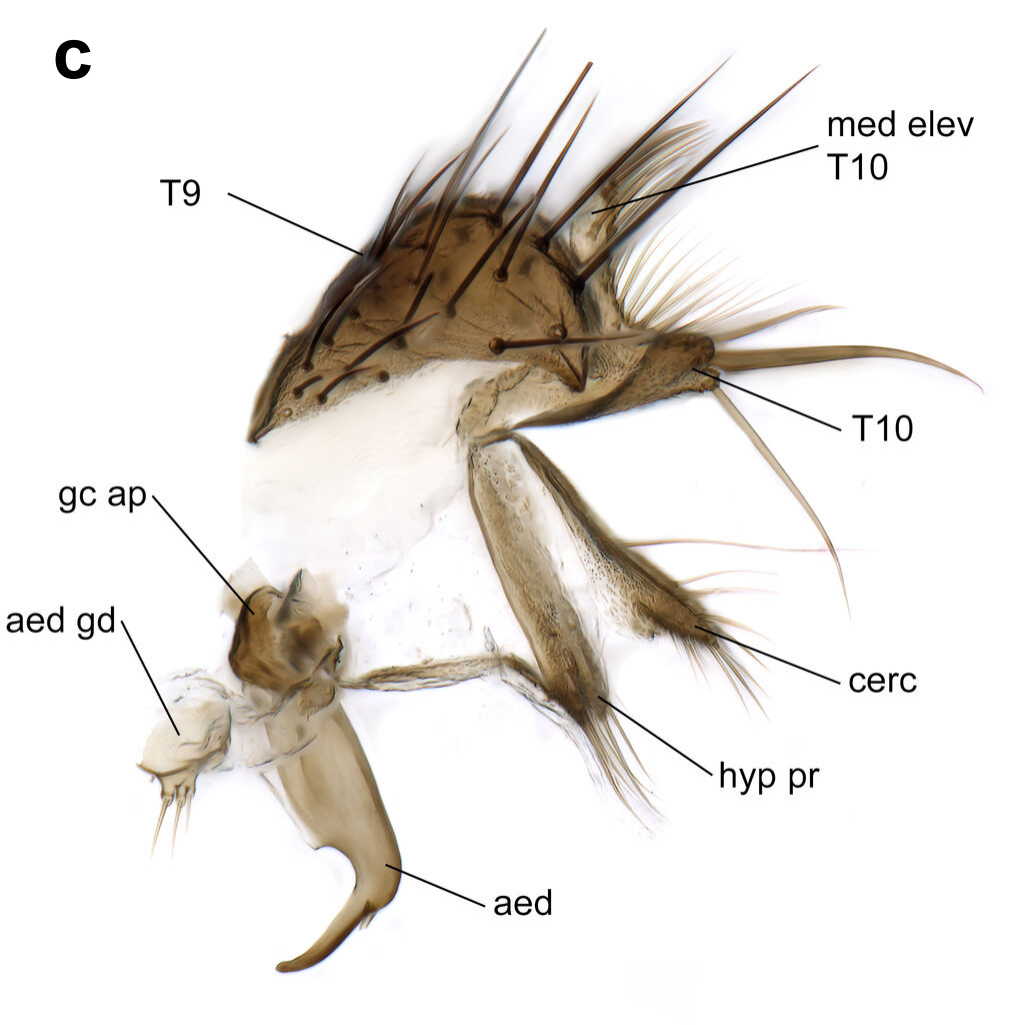
*Coelophthinialoraasi* sp. n., tergal segments and internal organs, lateral view

**Figure 3d.**
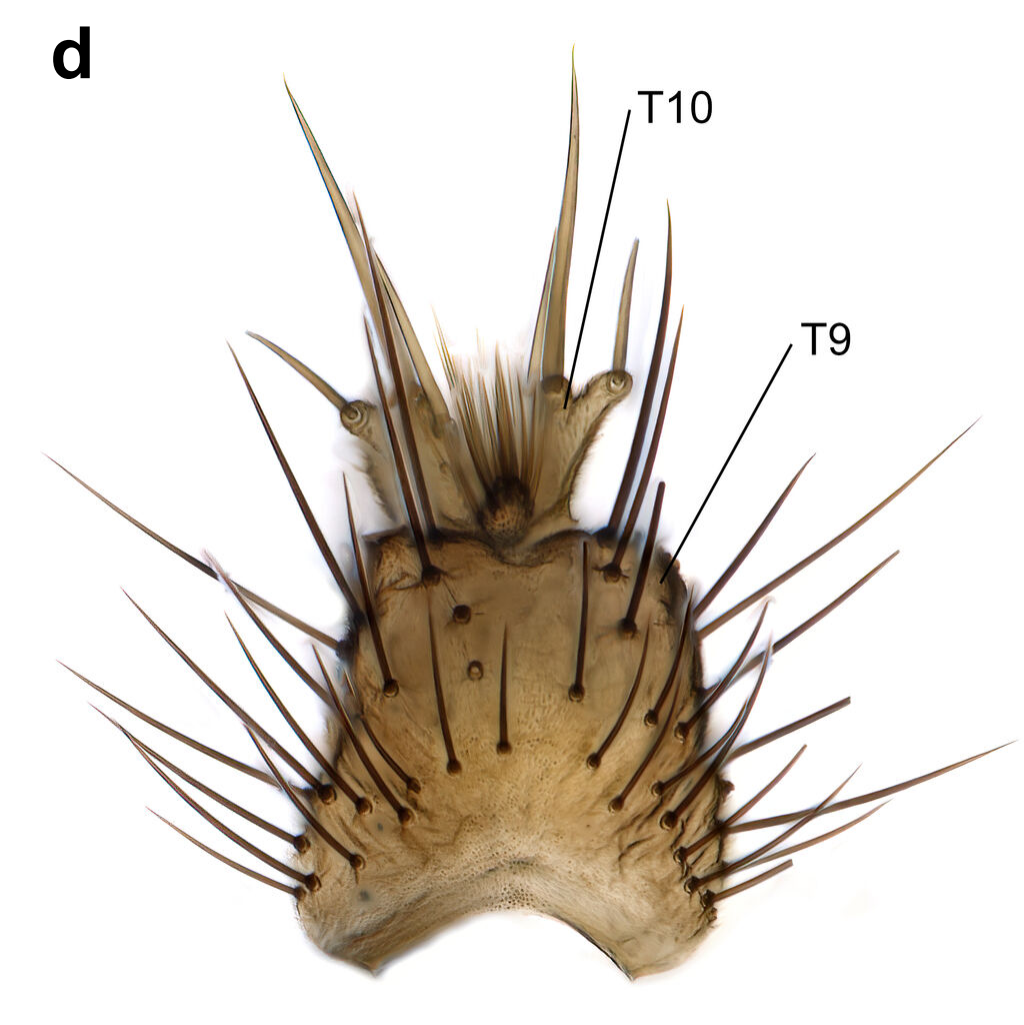
*Coelophthinialoraasi* sp. n., tergal segments, dorsal view

**Figure 3e.**
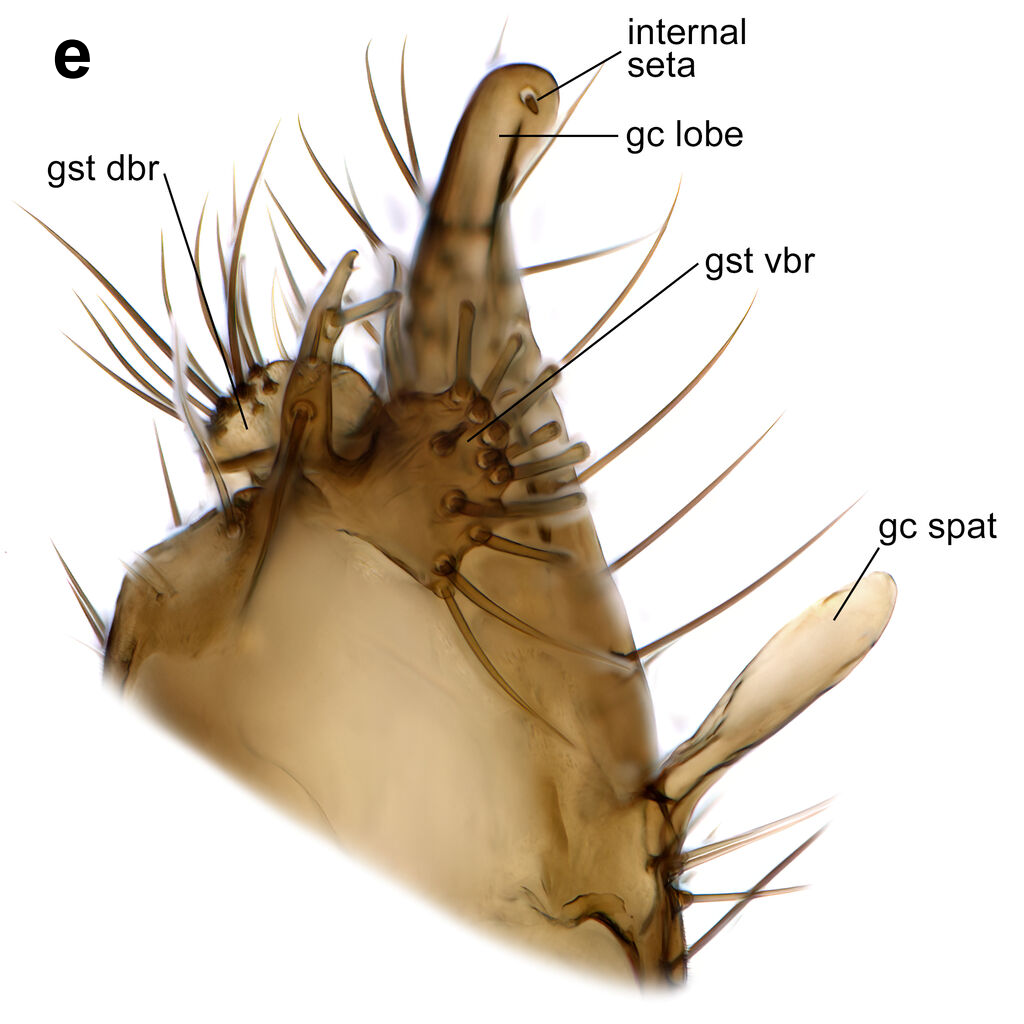
*Coelophthinialoraasi* sp. n., gonocoxite and gonostylus, internal view

**Figure 3f.**
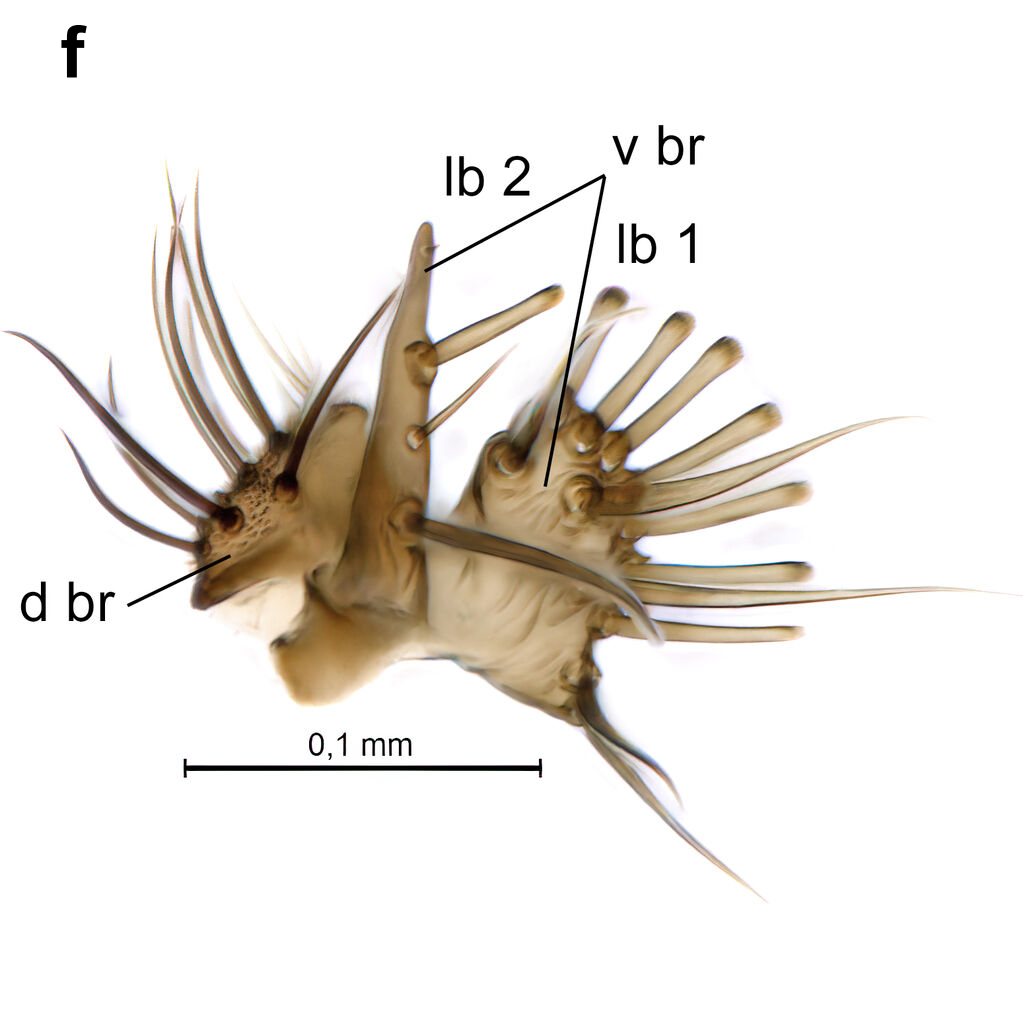
*Coelophthinialoraasi* sp. n., gonostylus, internal view.

**Figure 4a.**
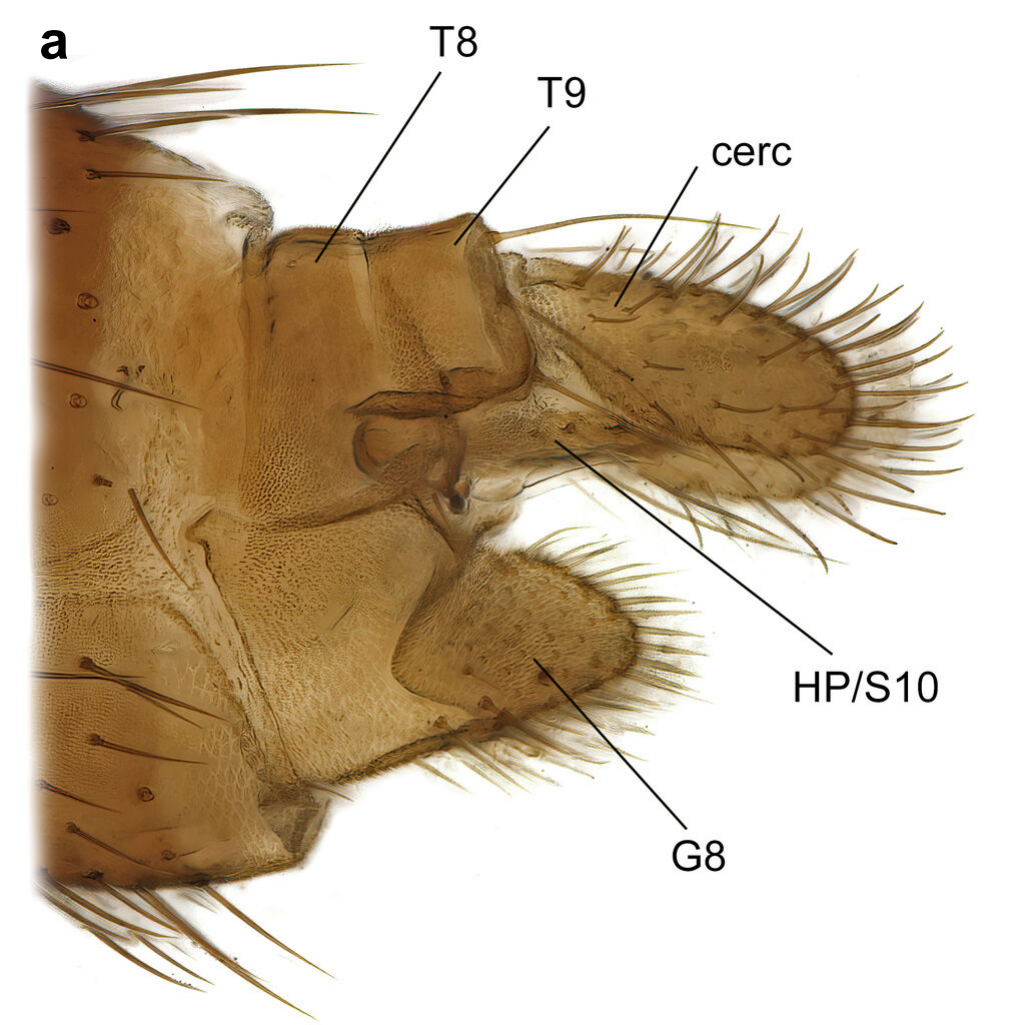
Terminalia, lateral view

**Figure 4b.**
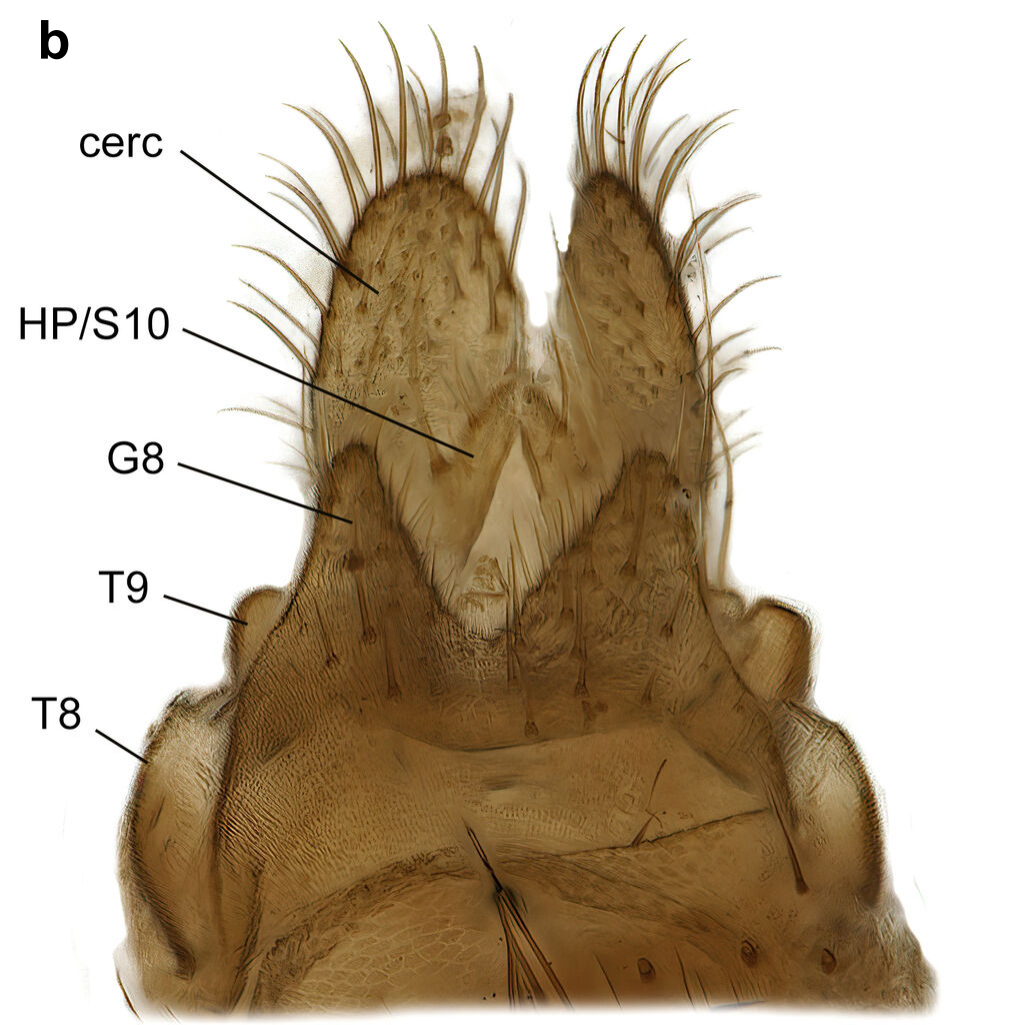
Terminalia, ventral view.

**Figure 5a.**
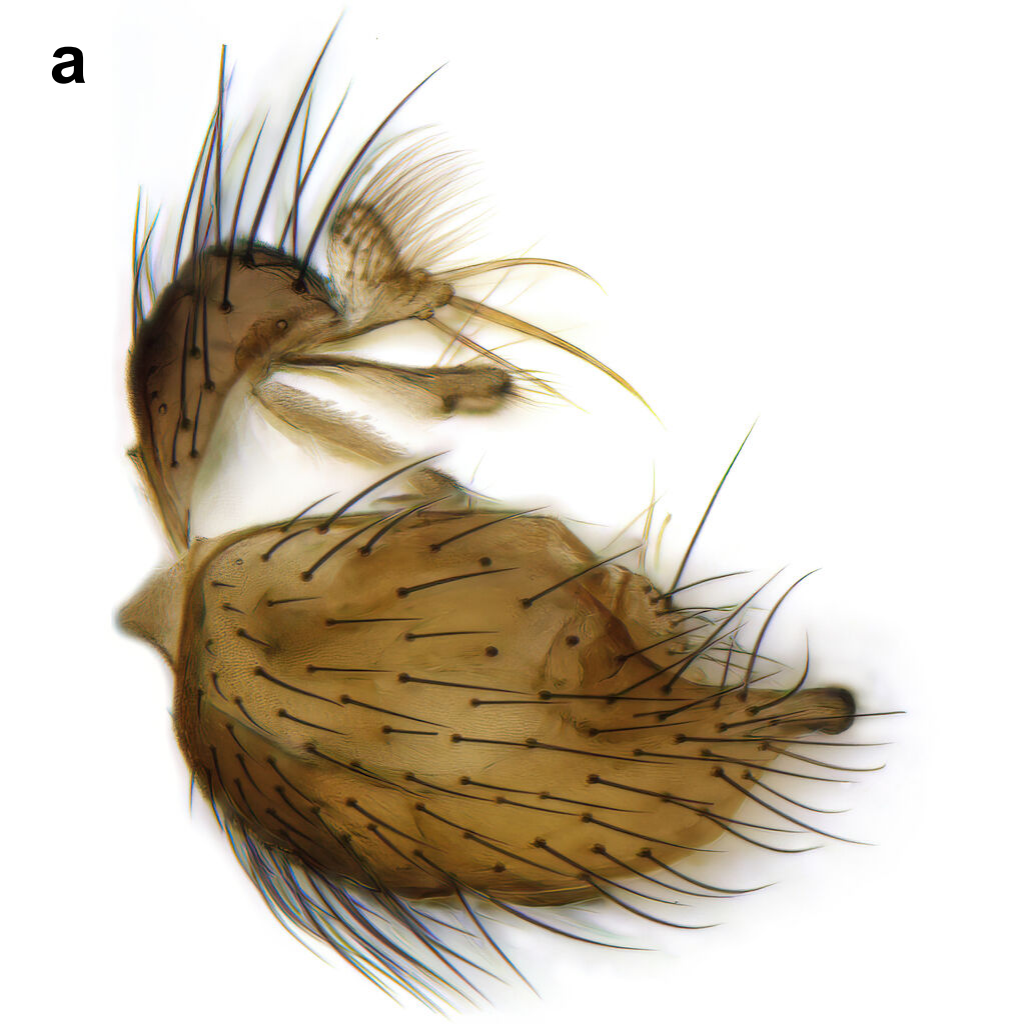
Lateral view

**Figure 5b.**
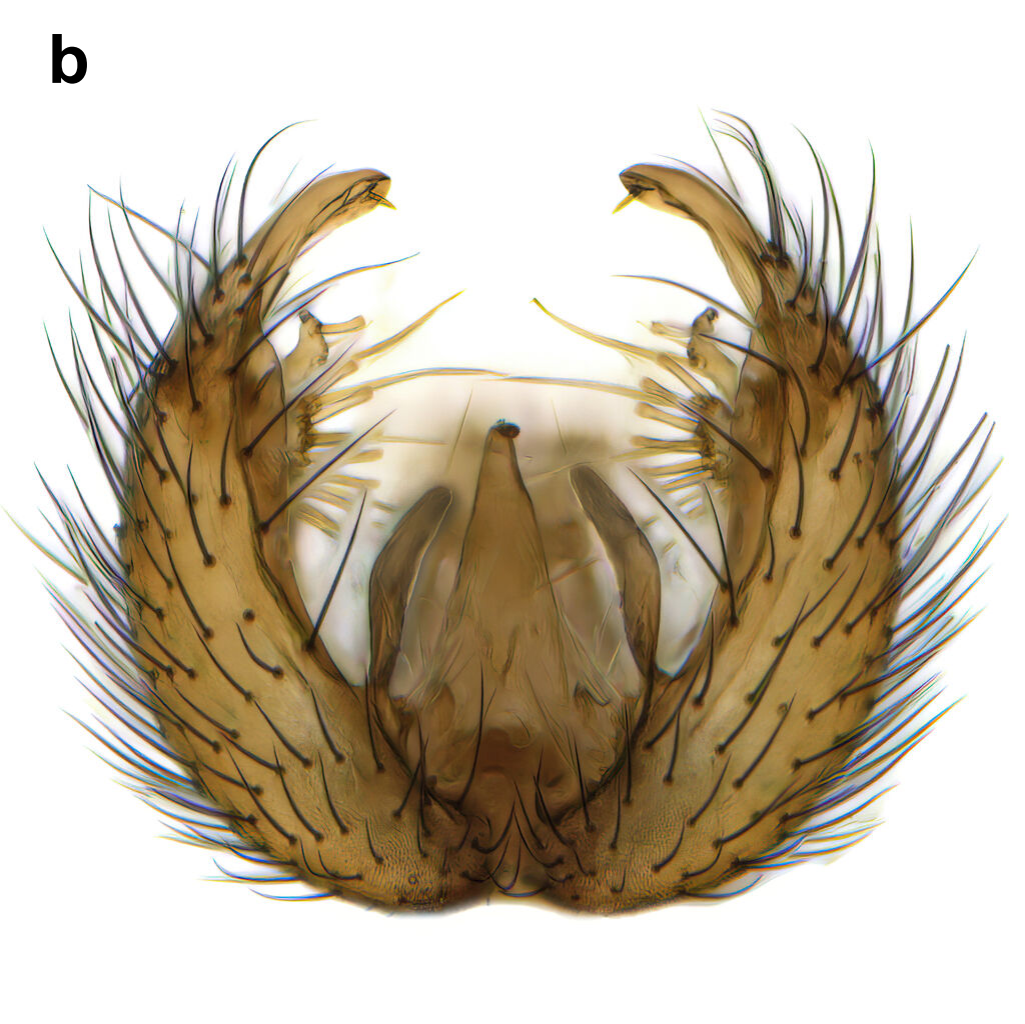
Ventral view

**Figure 5c.**
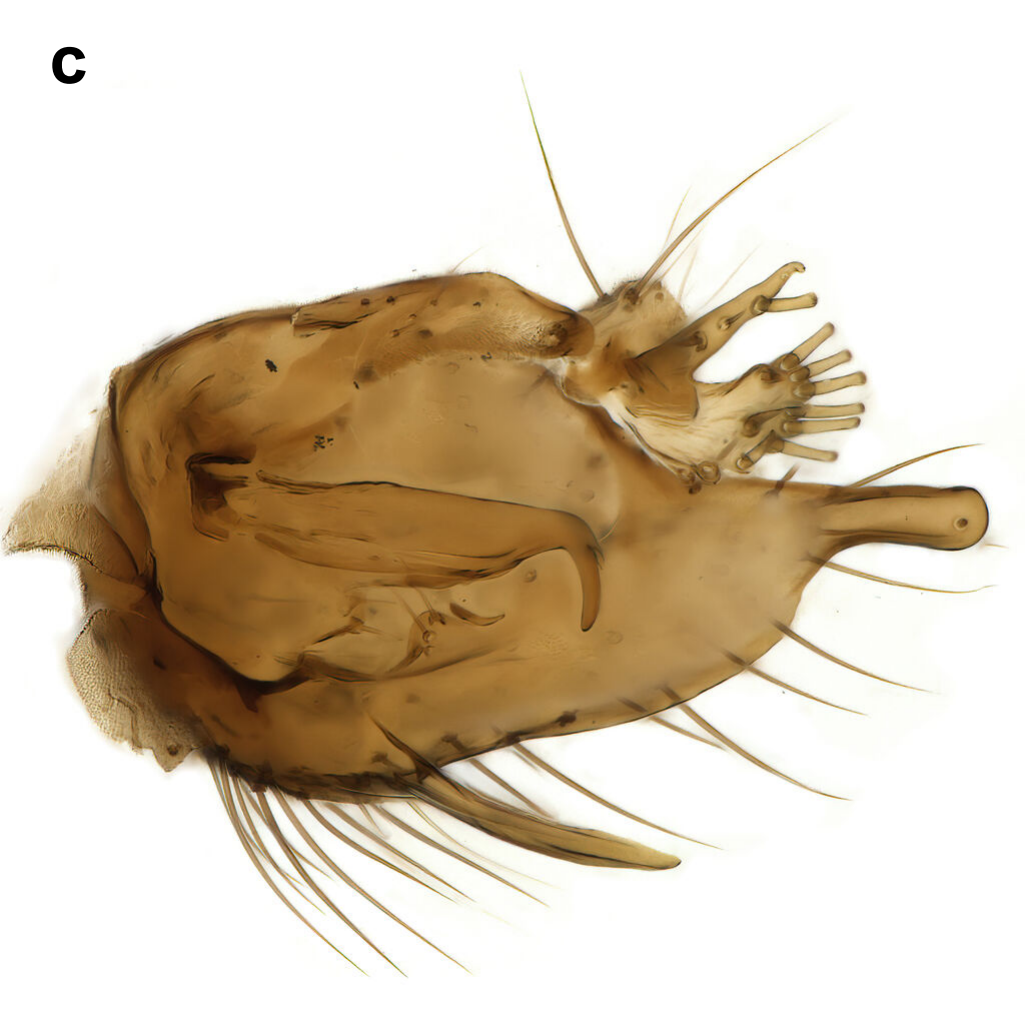
Internal, lateral view

**Figure 5d.**
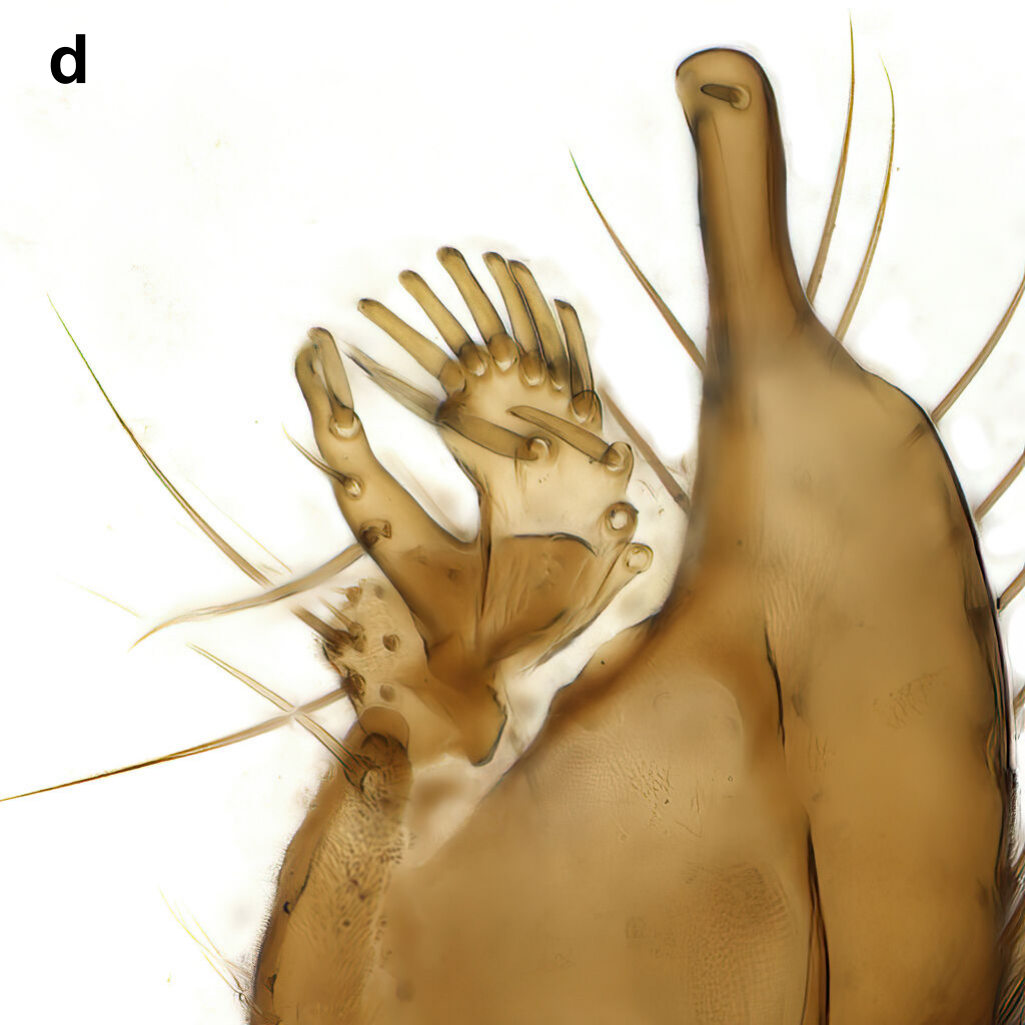
Gonostylus, enlarged

**Figure 5e.**
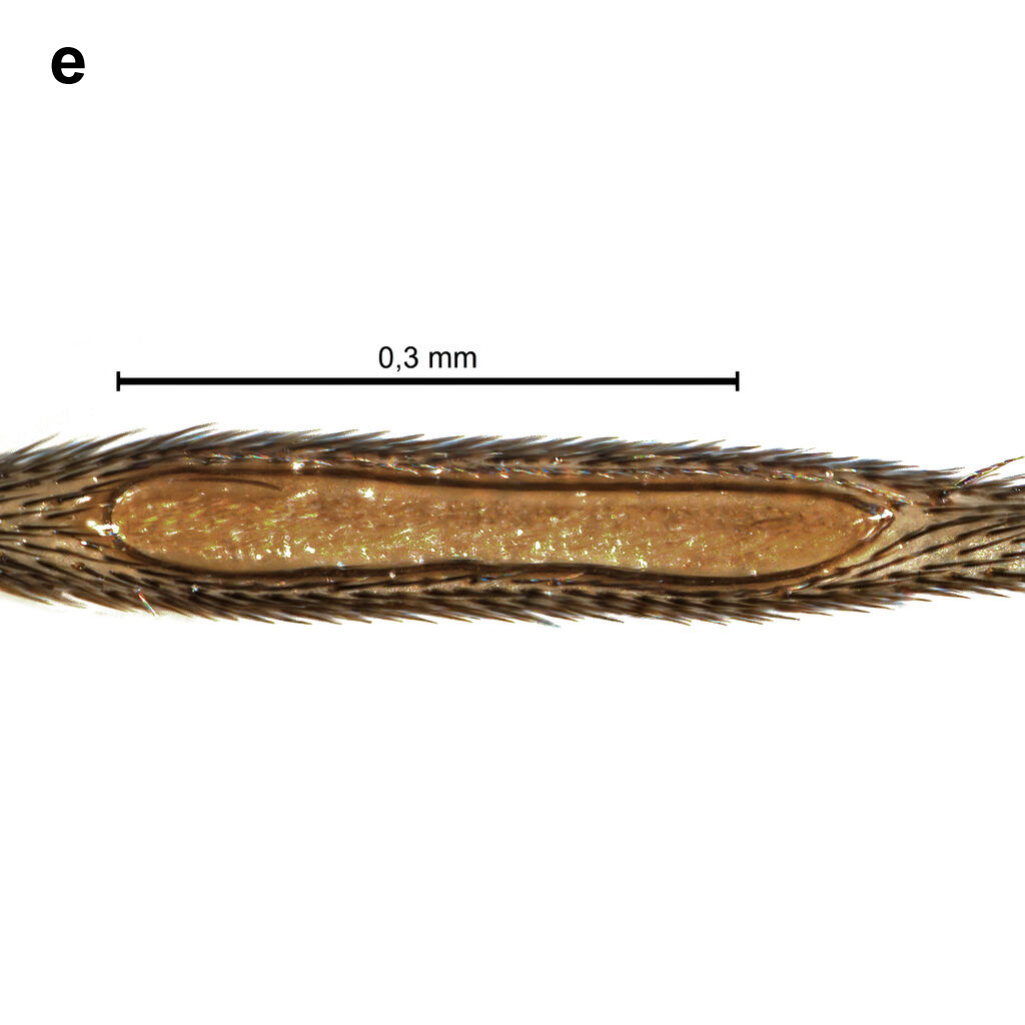
Mid-tibial organ.

**Figure 6a.**
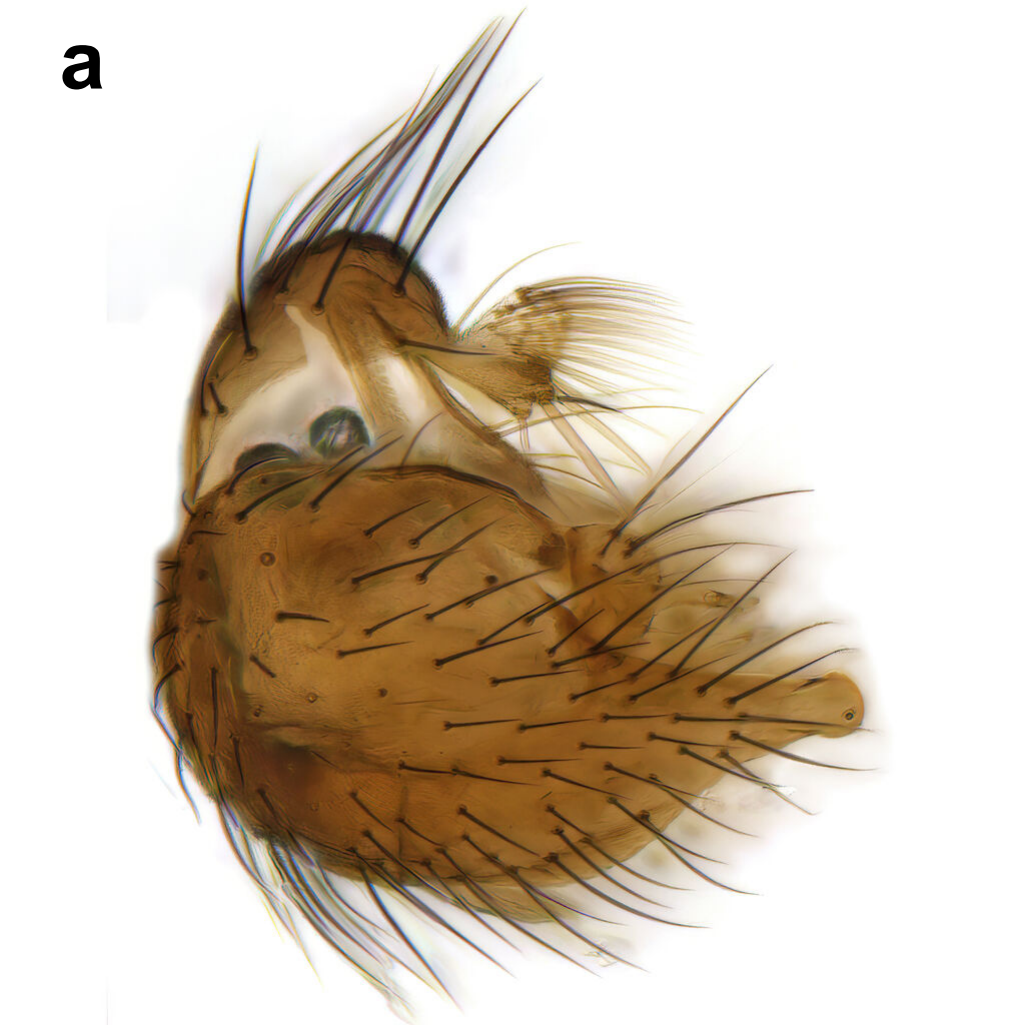
Lateral view

**Figure 6b.**
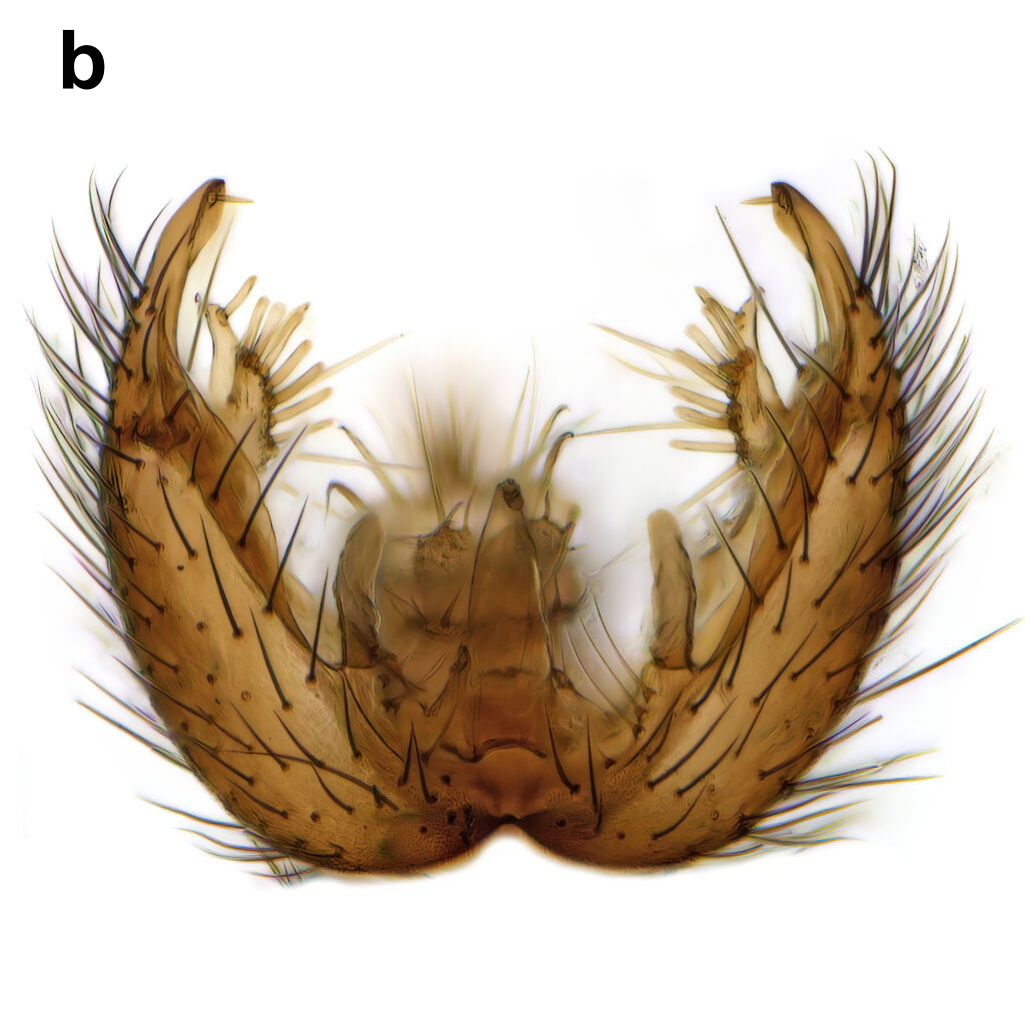
Ventral view

**Figure 6c.**
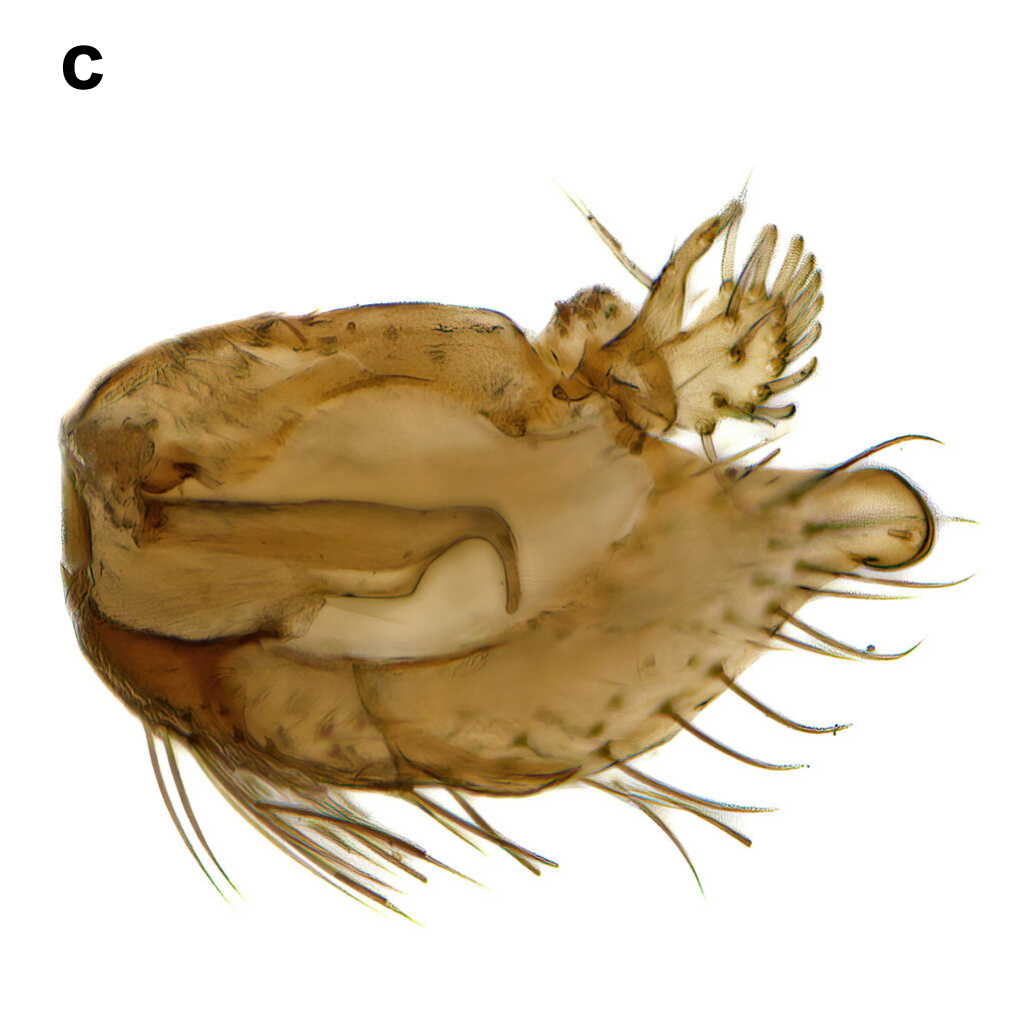
Internal, lateral view

**Figure 6d.**
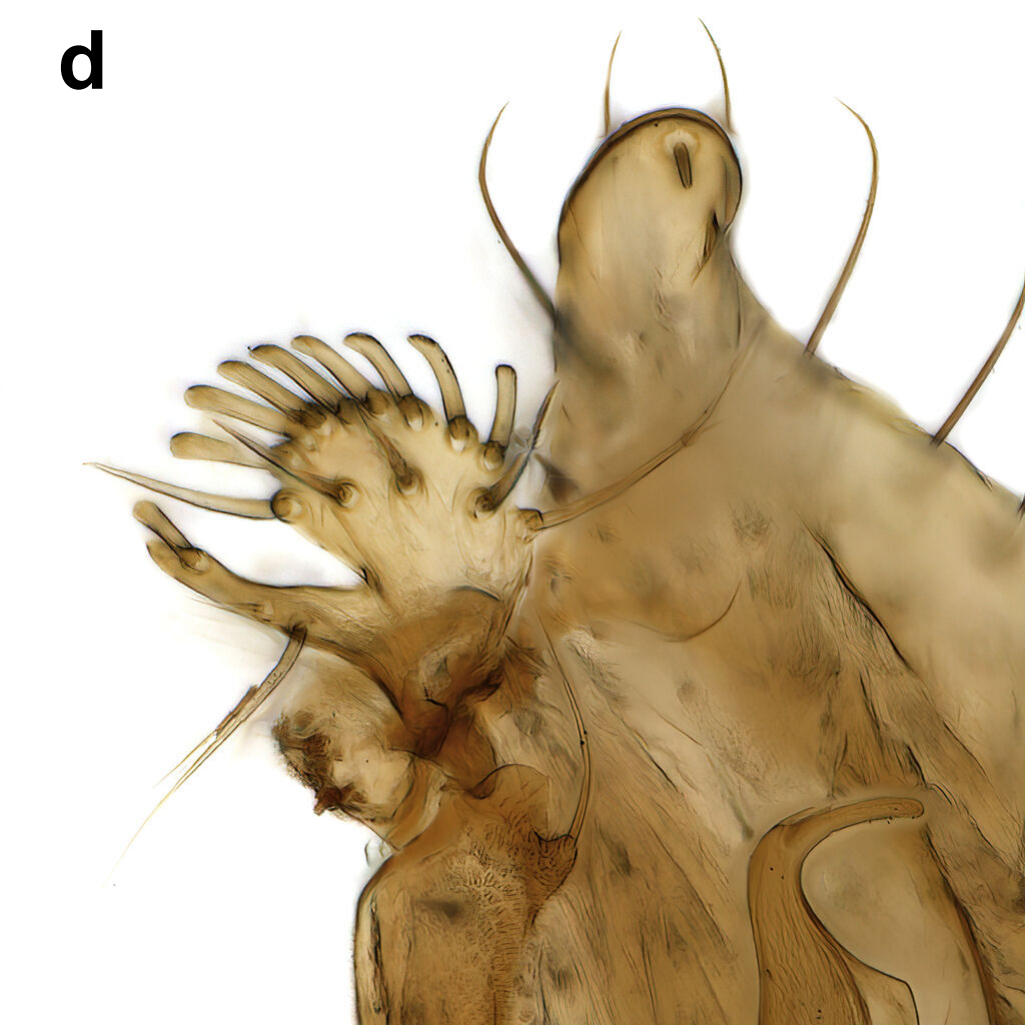
Gonostylus, enlarged, internal view

**Figure 6e.**
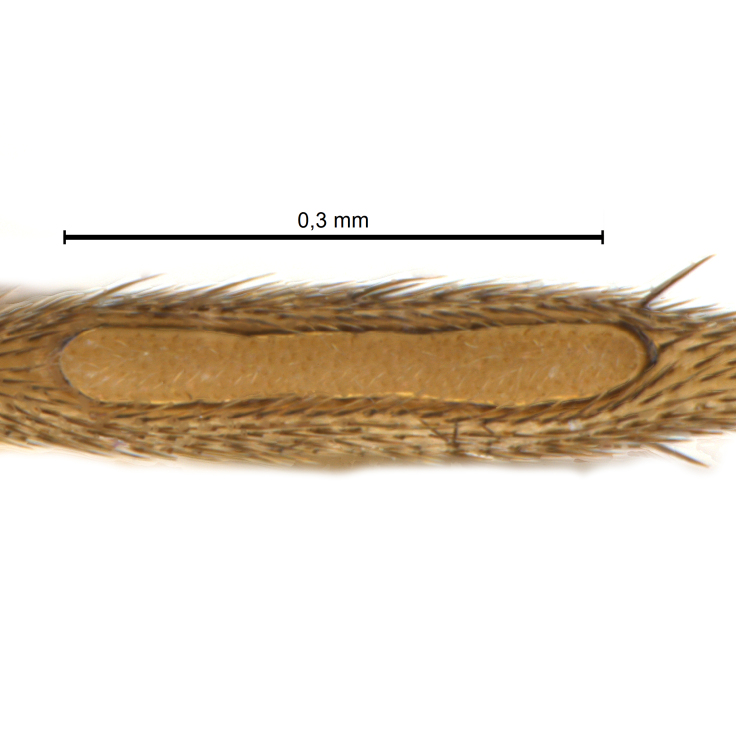
Mid-tibial organ.

**Figure 7a.**
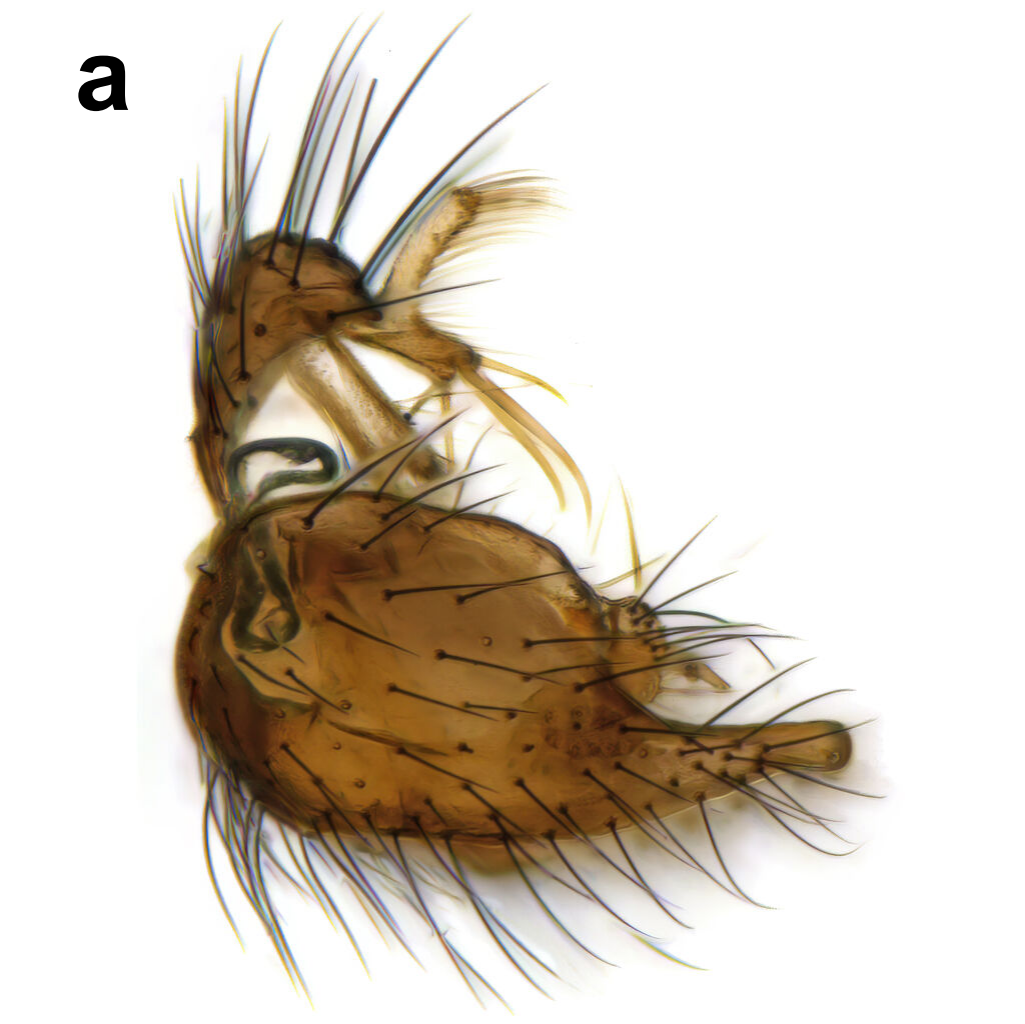
Lateral view

**Figure 7b.**
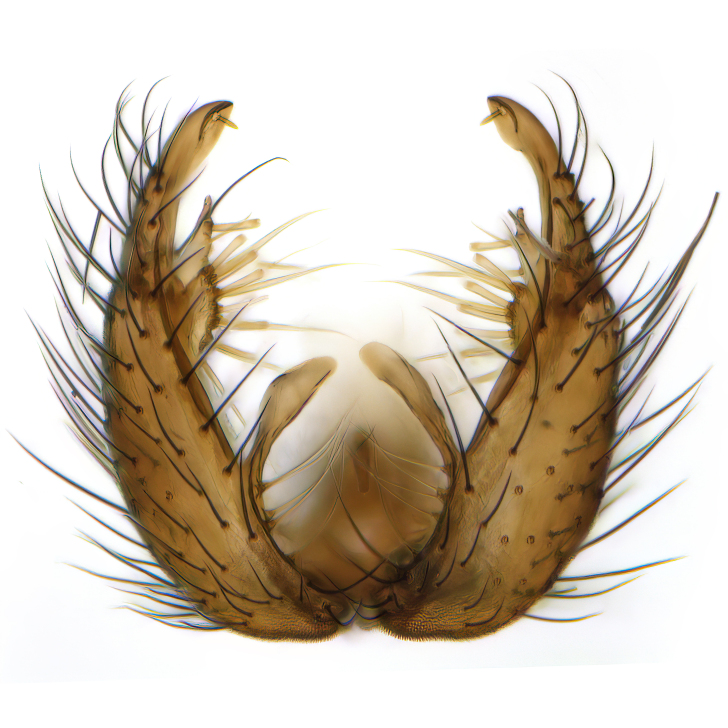
Ventral view

**Figure 7c.**
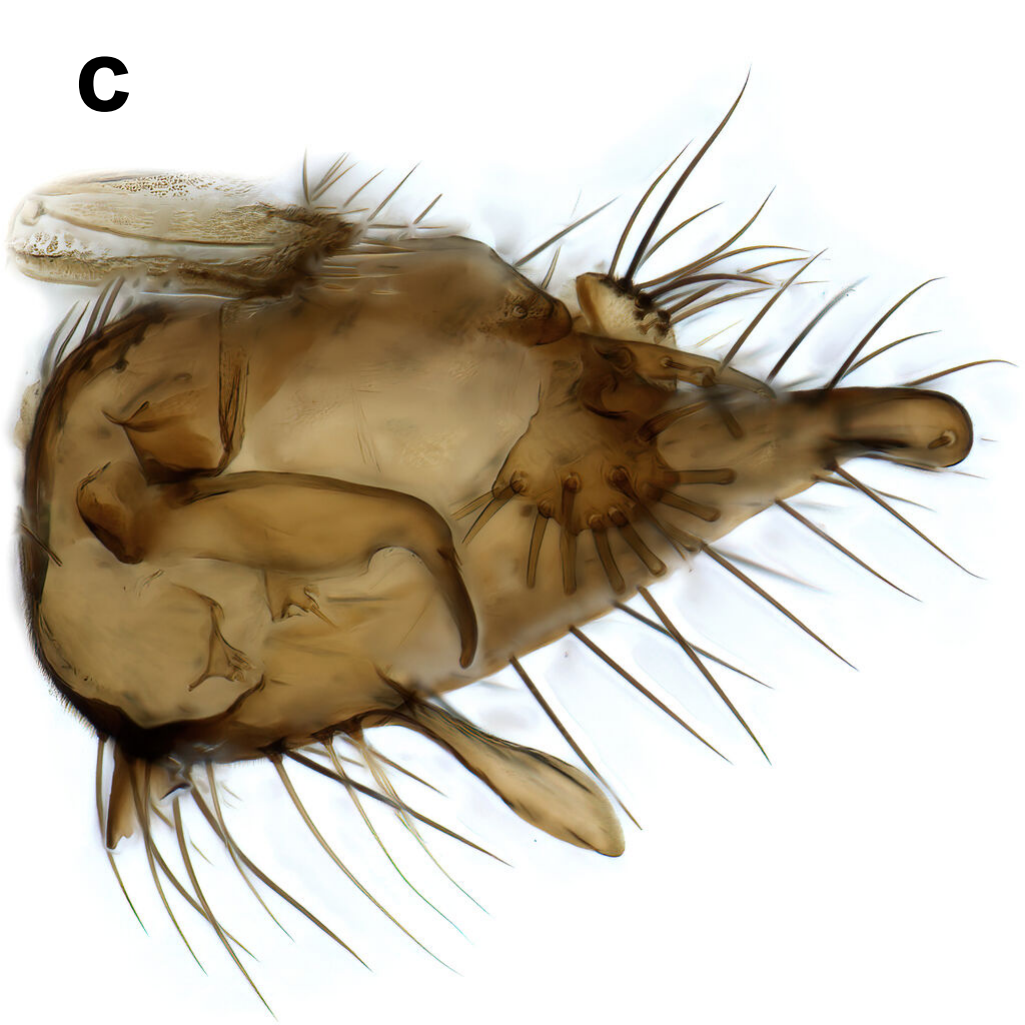
Internal, lateral view

**Figure 7d.**
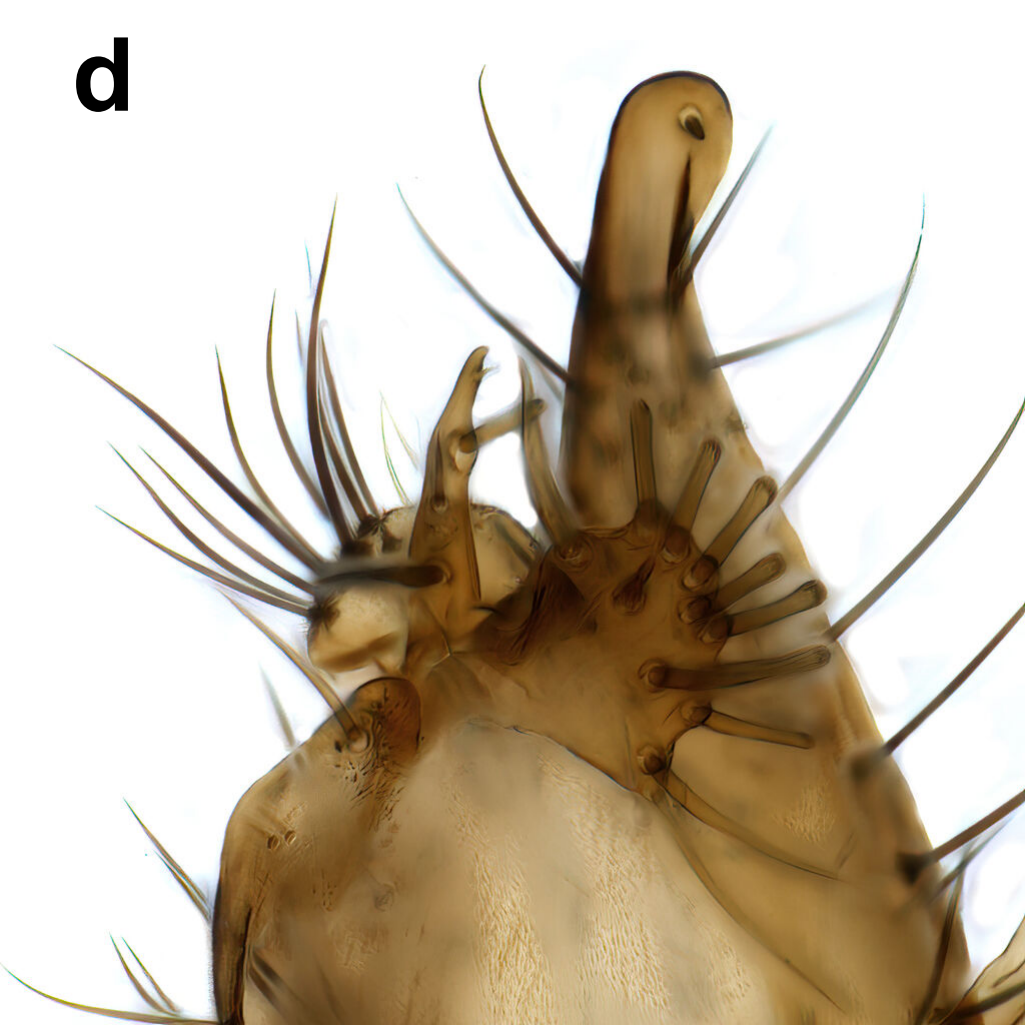
Gonostylus, enlarged, internal view

**Figure 7e.**
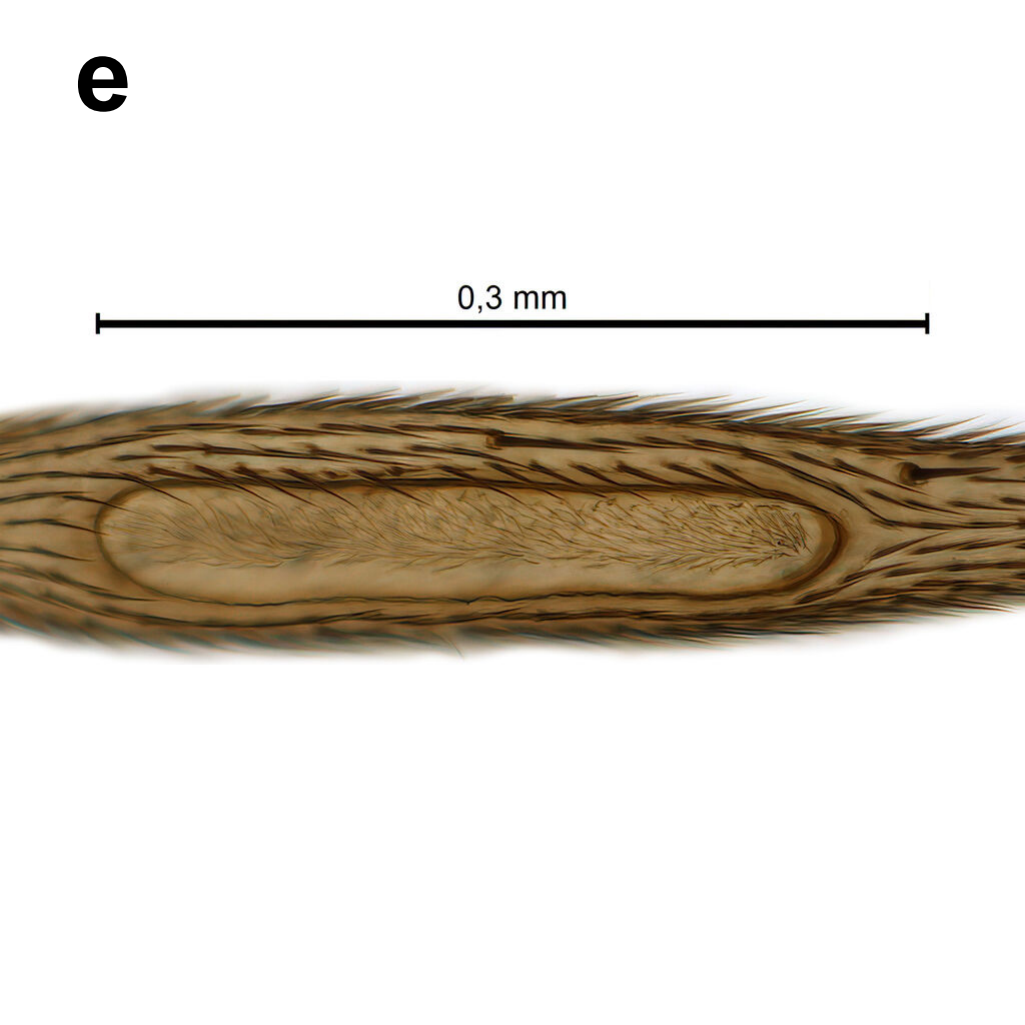
Mid-tibial organ.

**Figure 8a.**
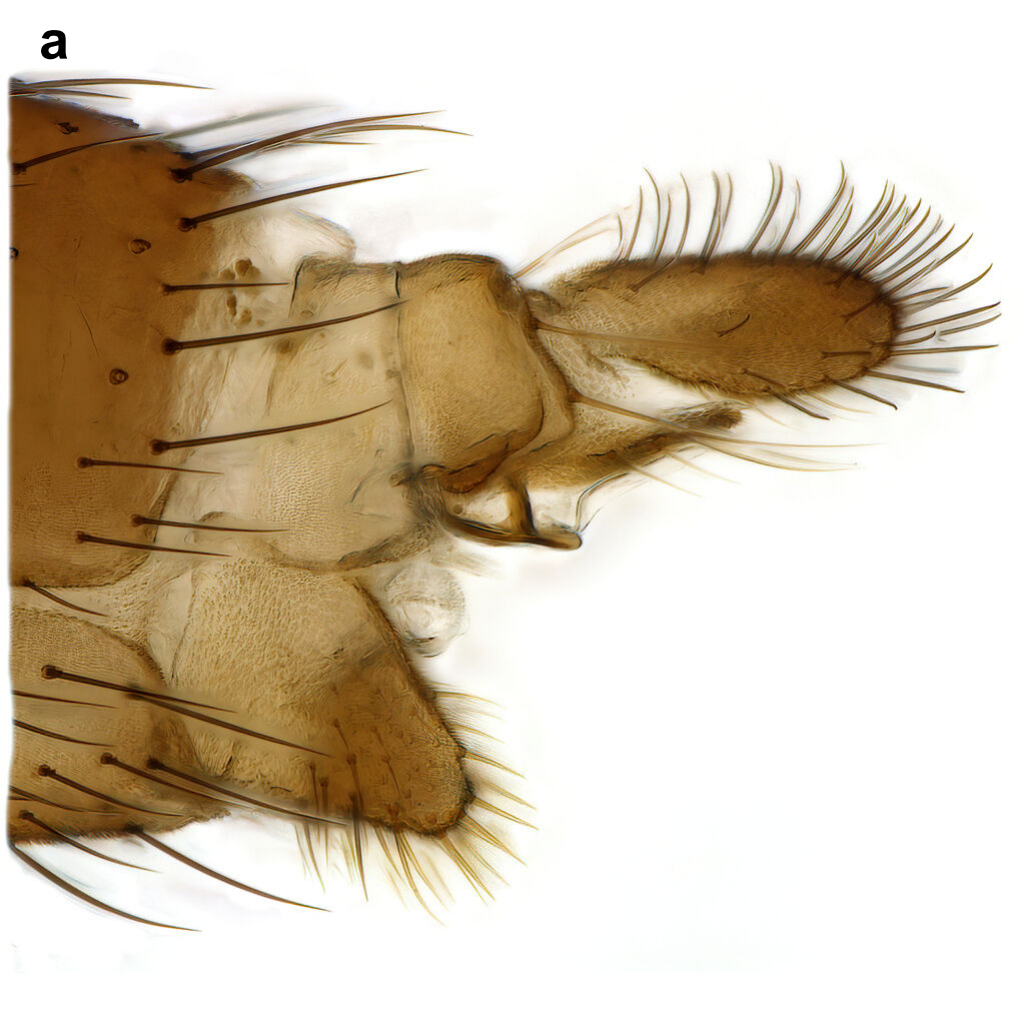
Lateral view

**Figure 8b.**
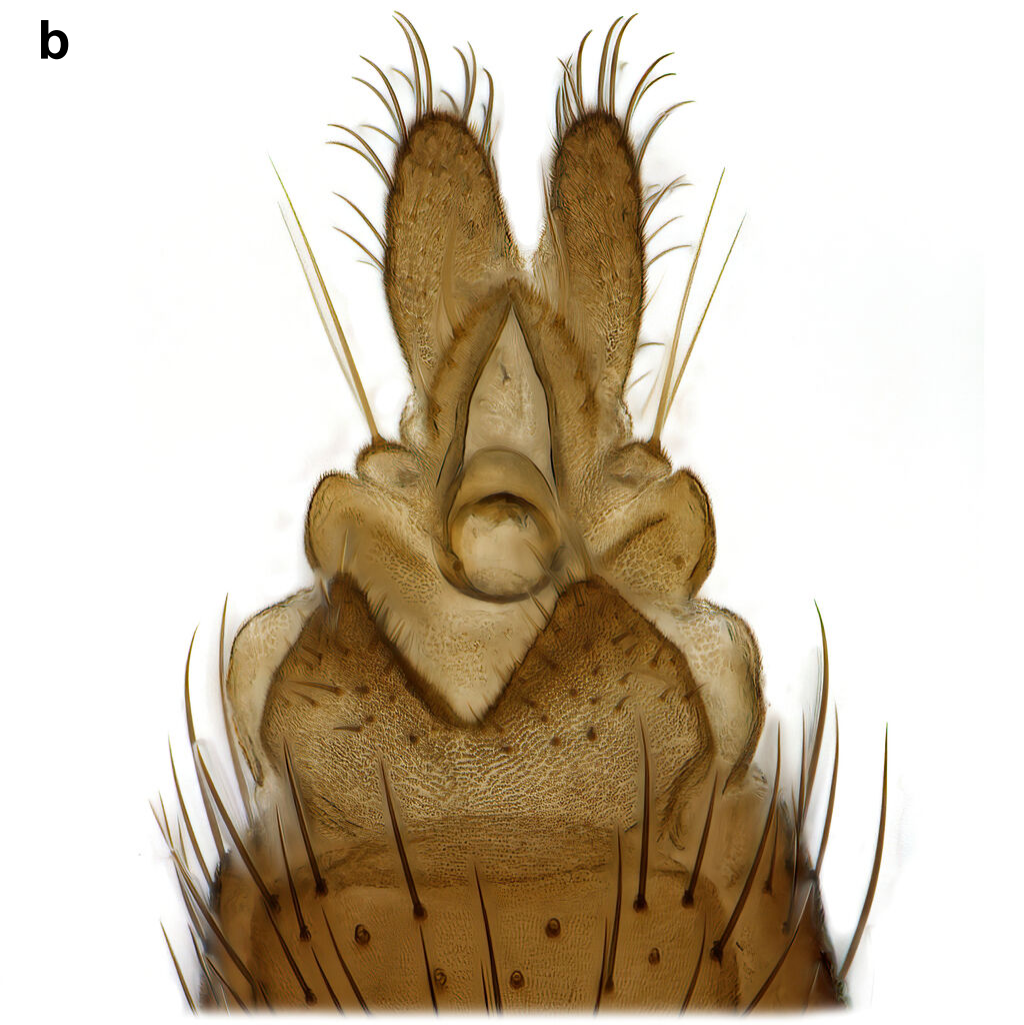
Ventral view

**Figure 8c.**
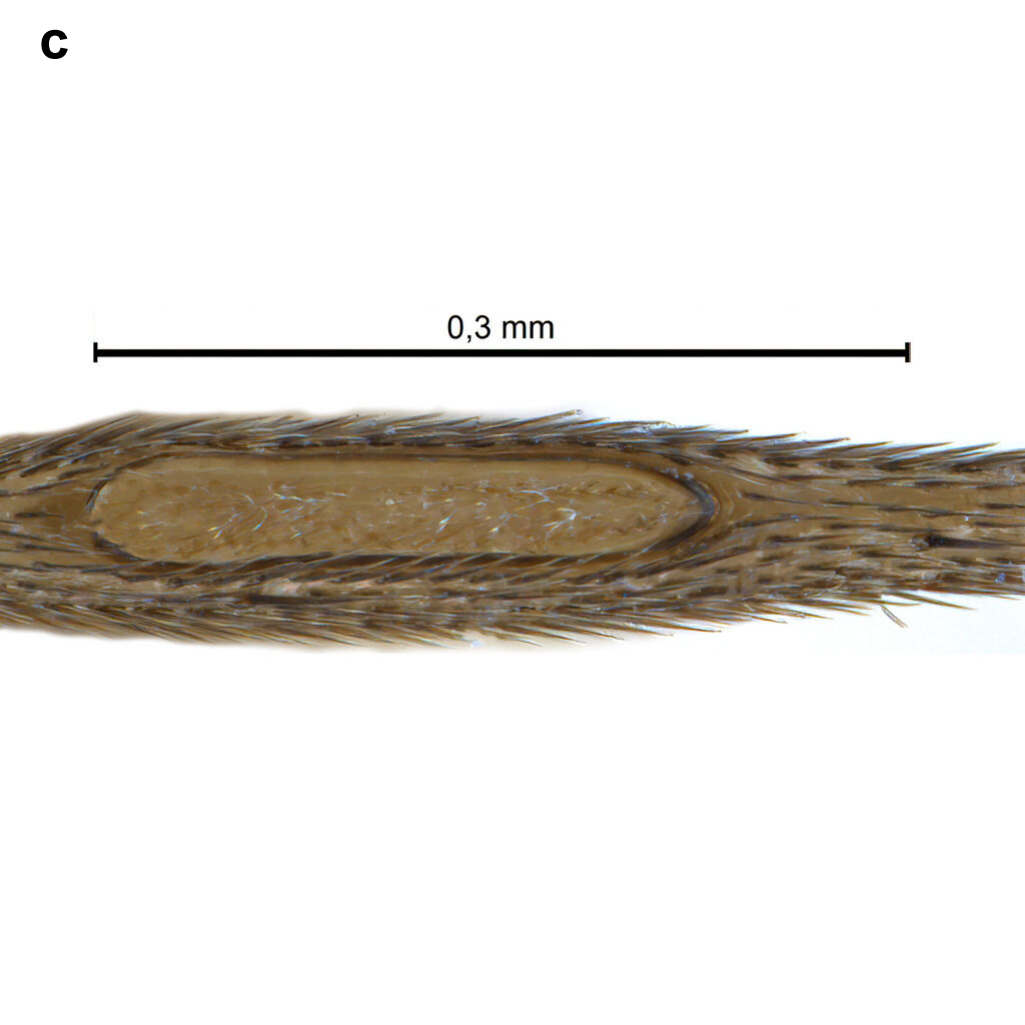
Mid-tibial organ.

**Figure 9a.**
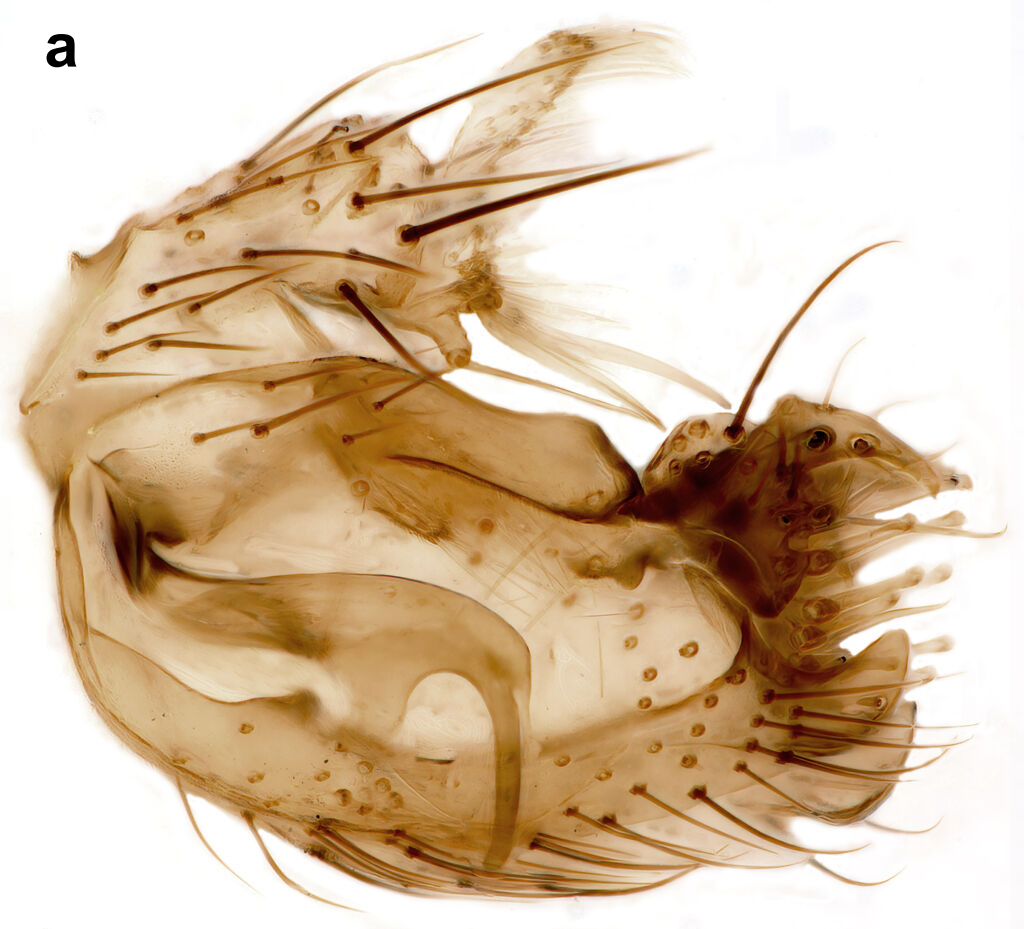
Lateral view

**Figure 9b.**
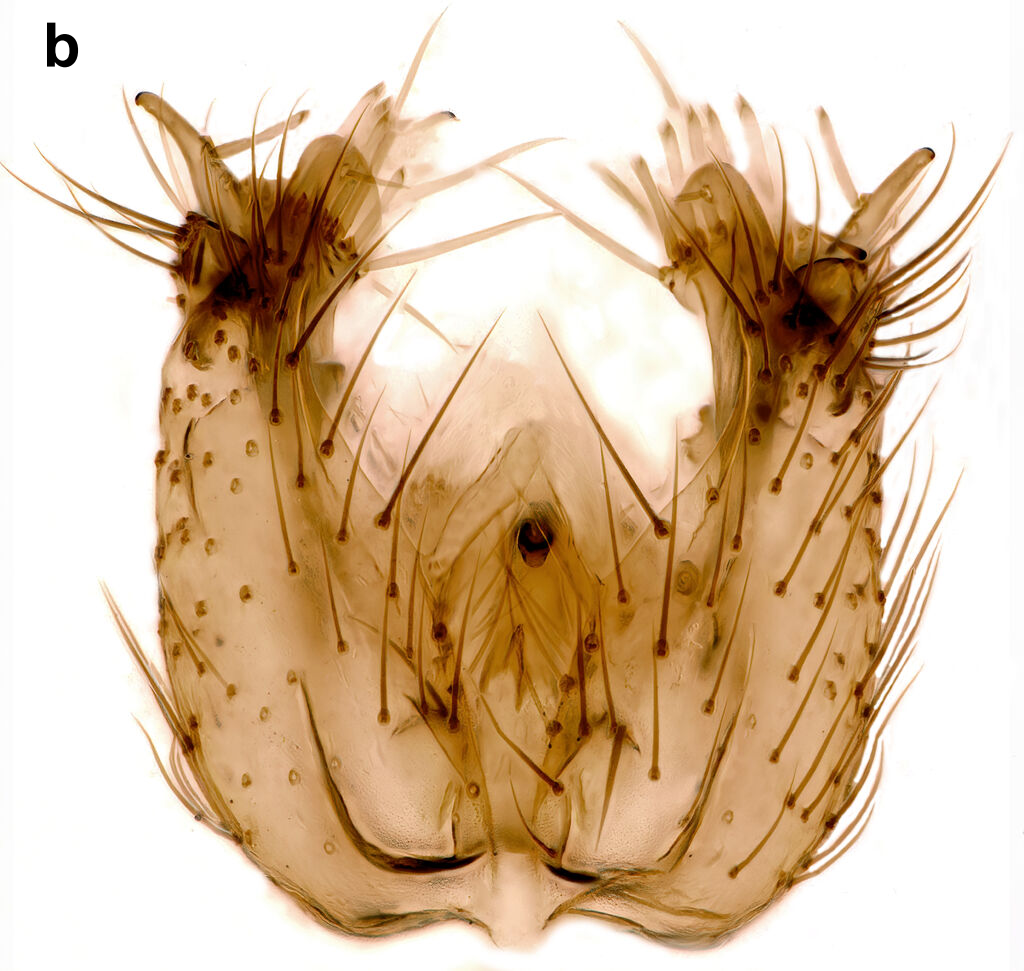
Ventral view

**Figure 9c.**
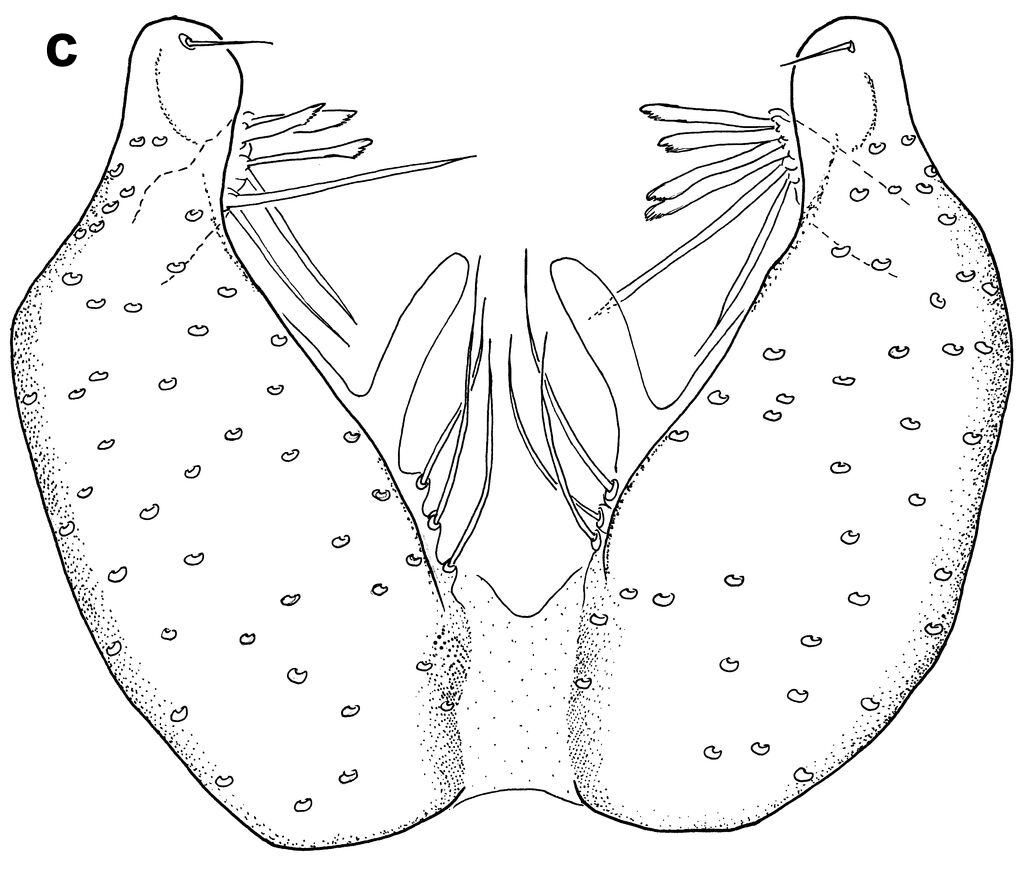
Ventral view

**Figure 9d.**
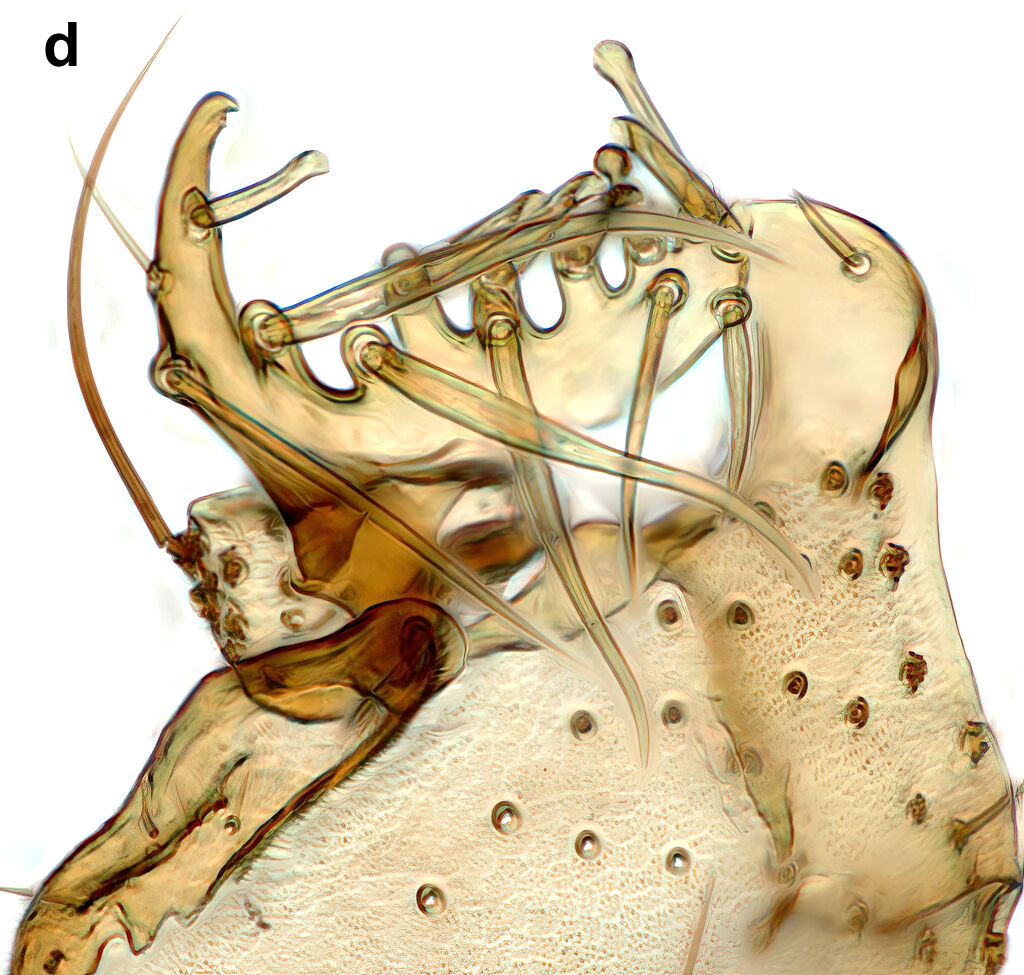
Gonostylus, enlarged, internal view

**Figure 9e.**
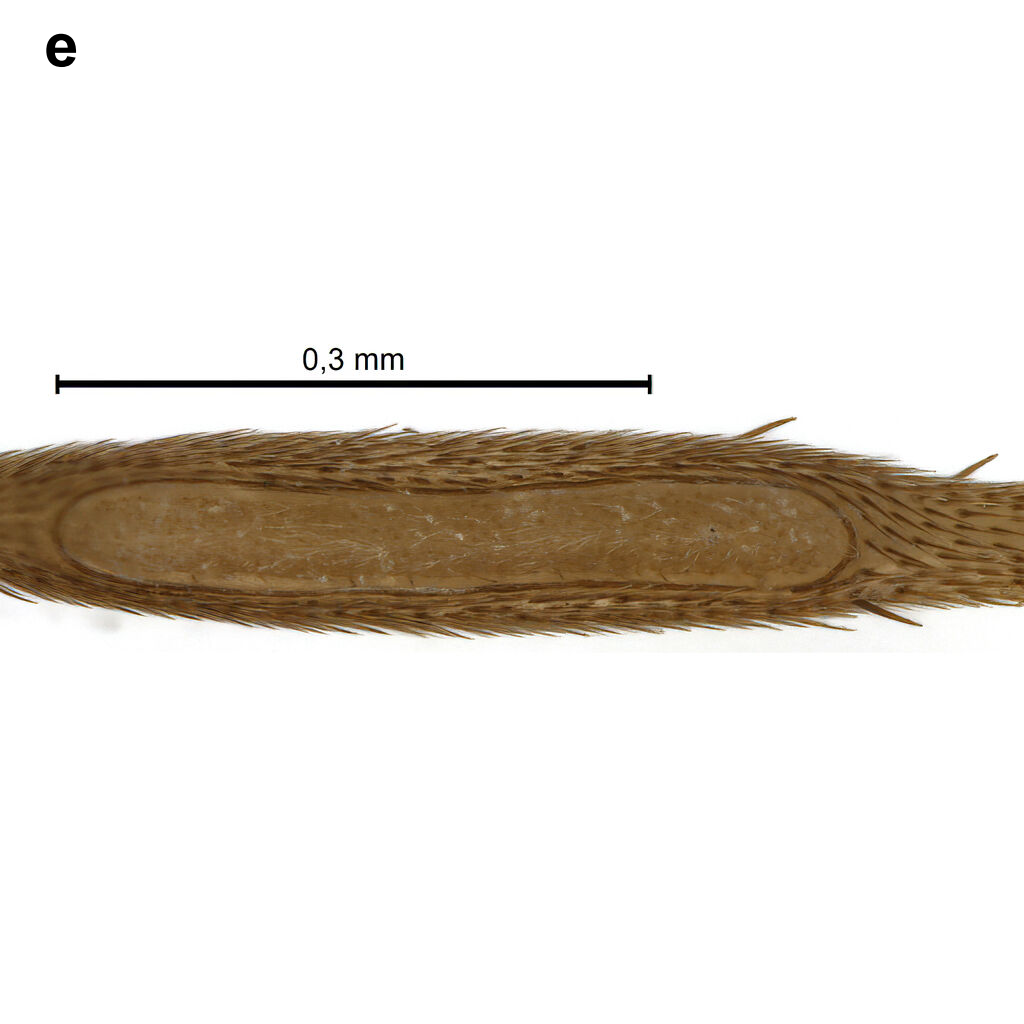
Mid-tibial organ.

**Figure 10.**
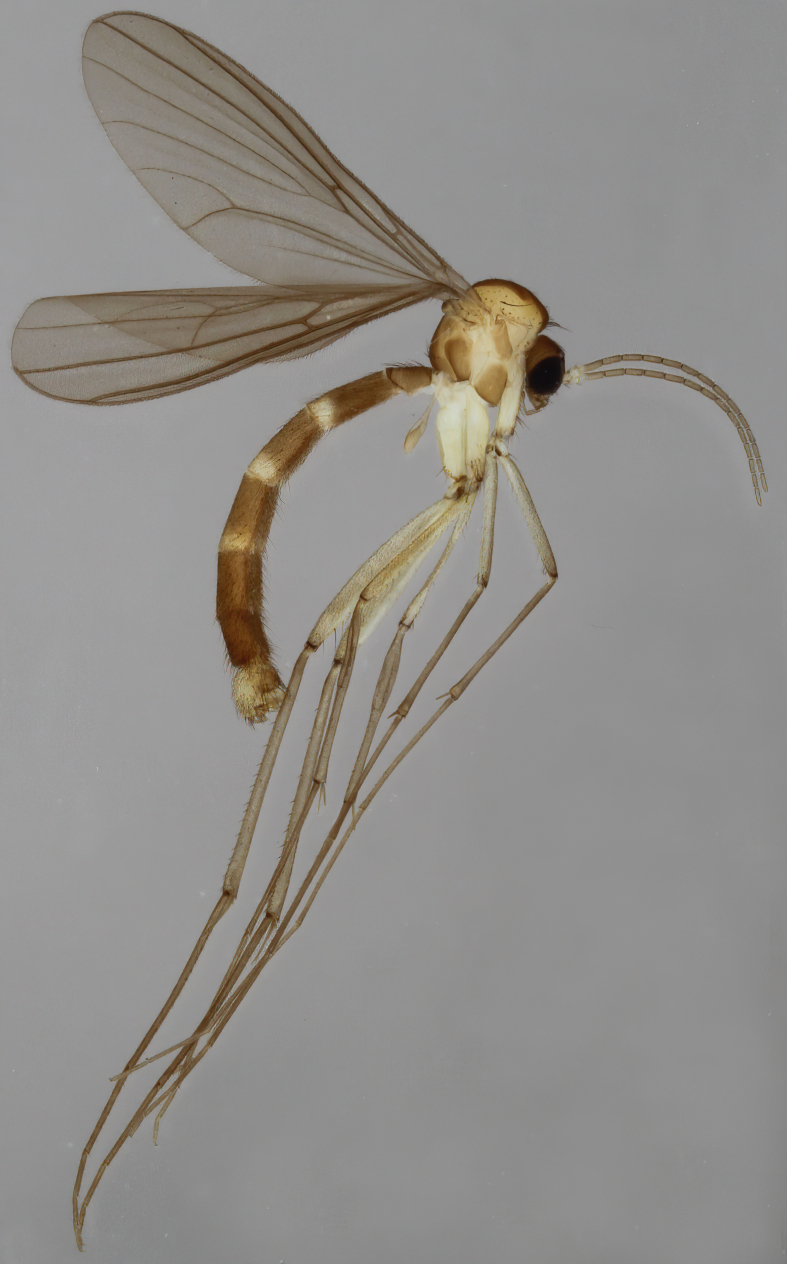
*Coelophthiniaitoae* sp. n., habitus (IZBE0251605: Akan-cho, Japan).

**Figure 11a.**
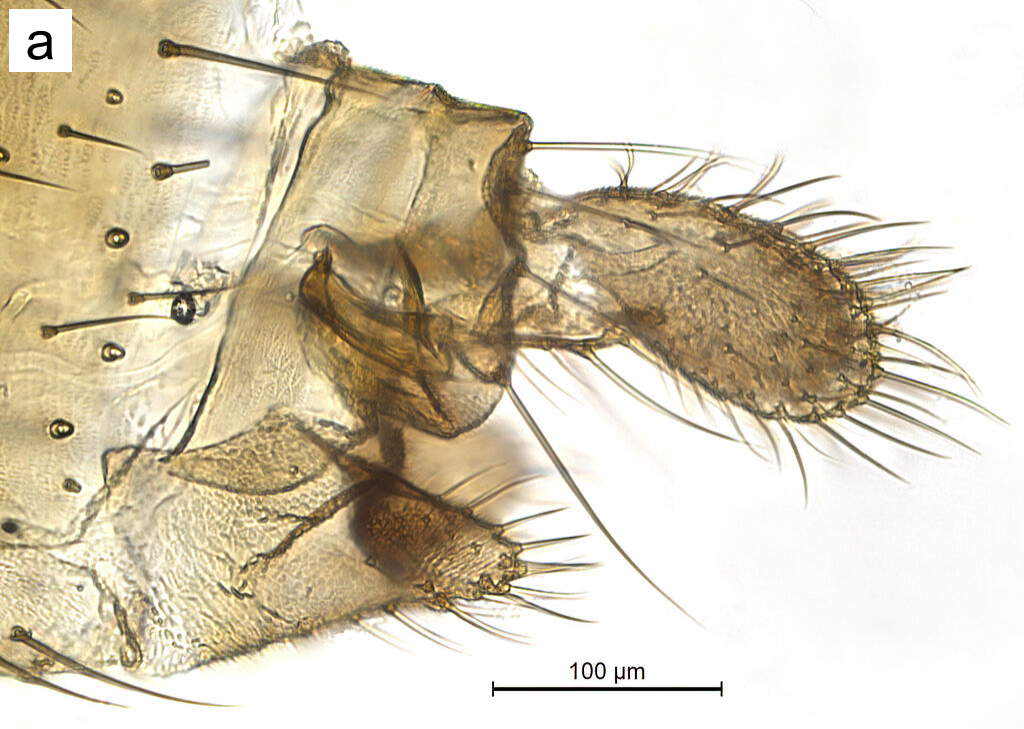
Lateral view

**Figure 11b.**
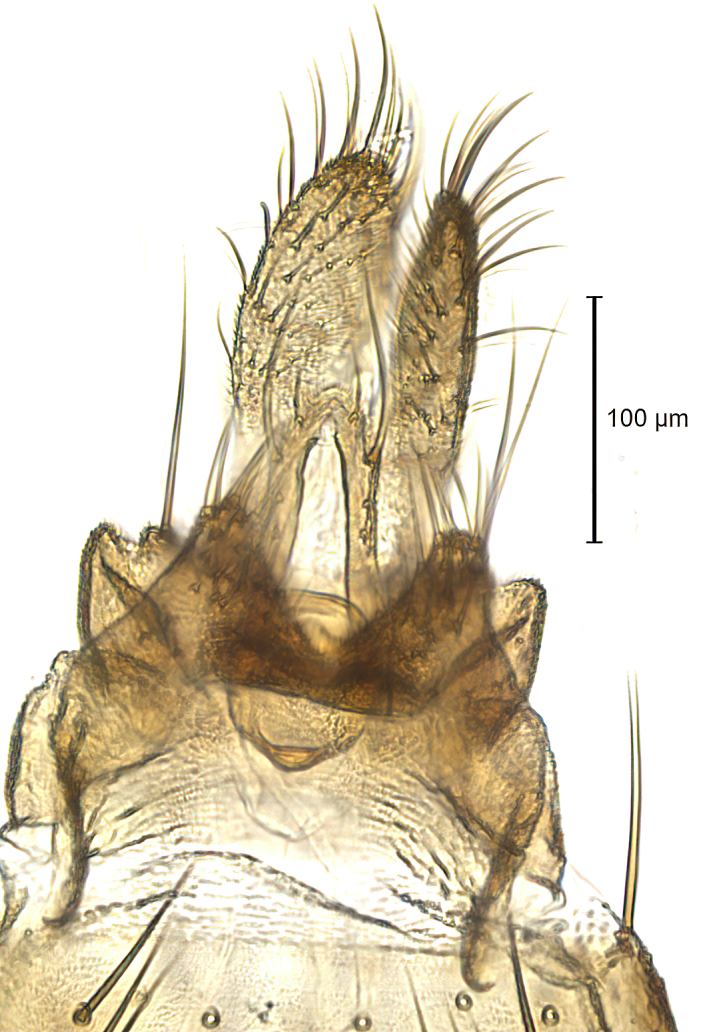
Ventral view

**Figure 11c.**
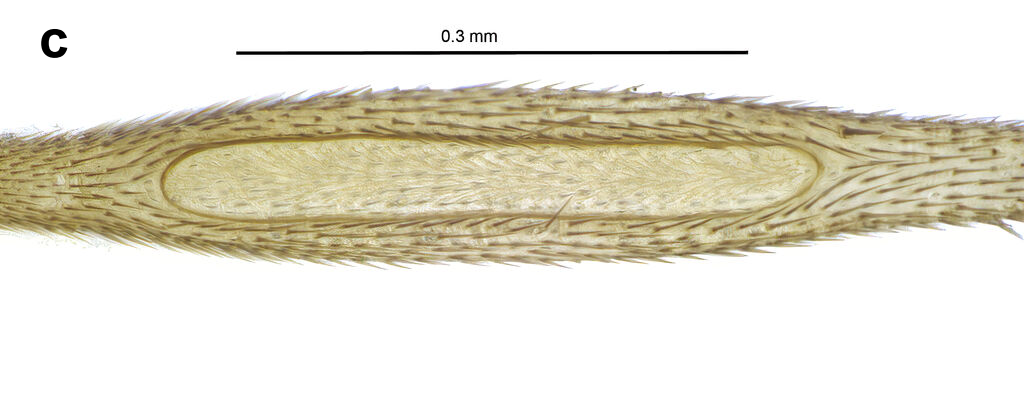
Mid-tibial organ.

**Figure 12a.**
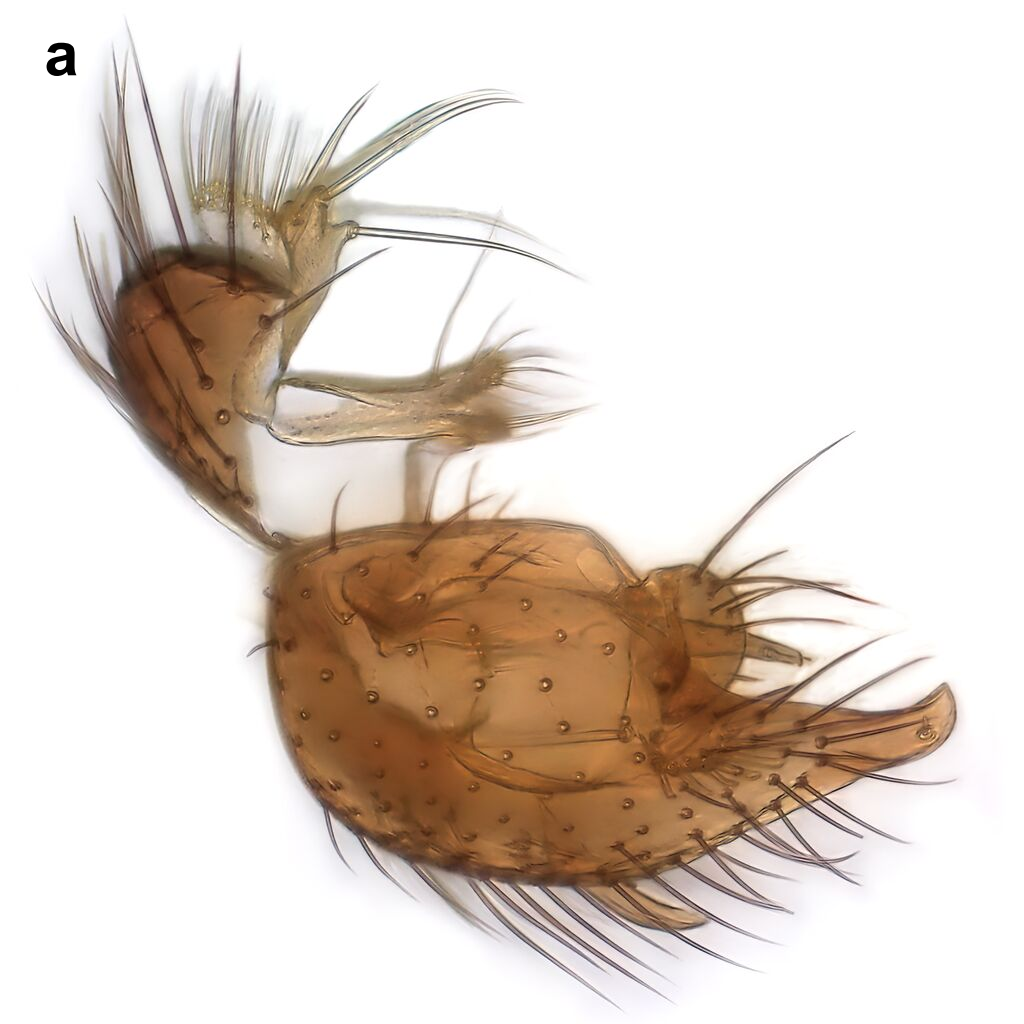
Lateral view

**Figure 12b.**
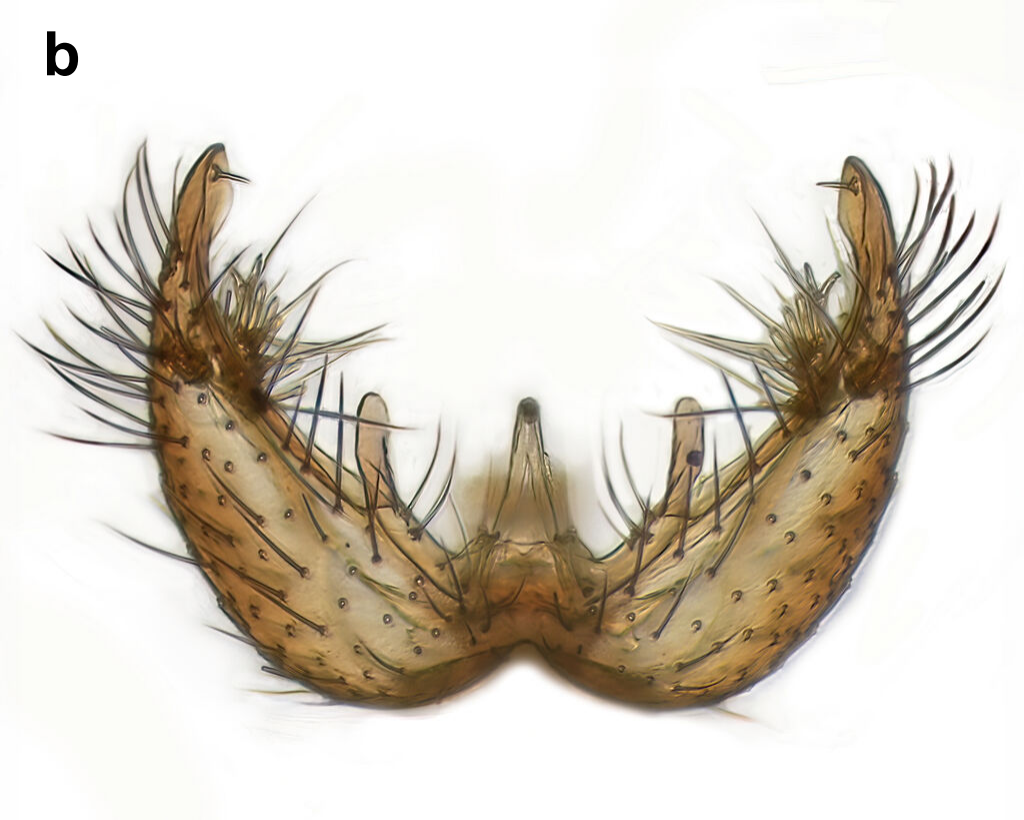
Ventral view

**Figure 12c.**
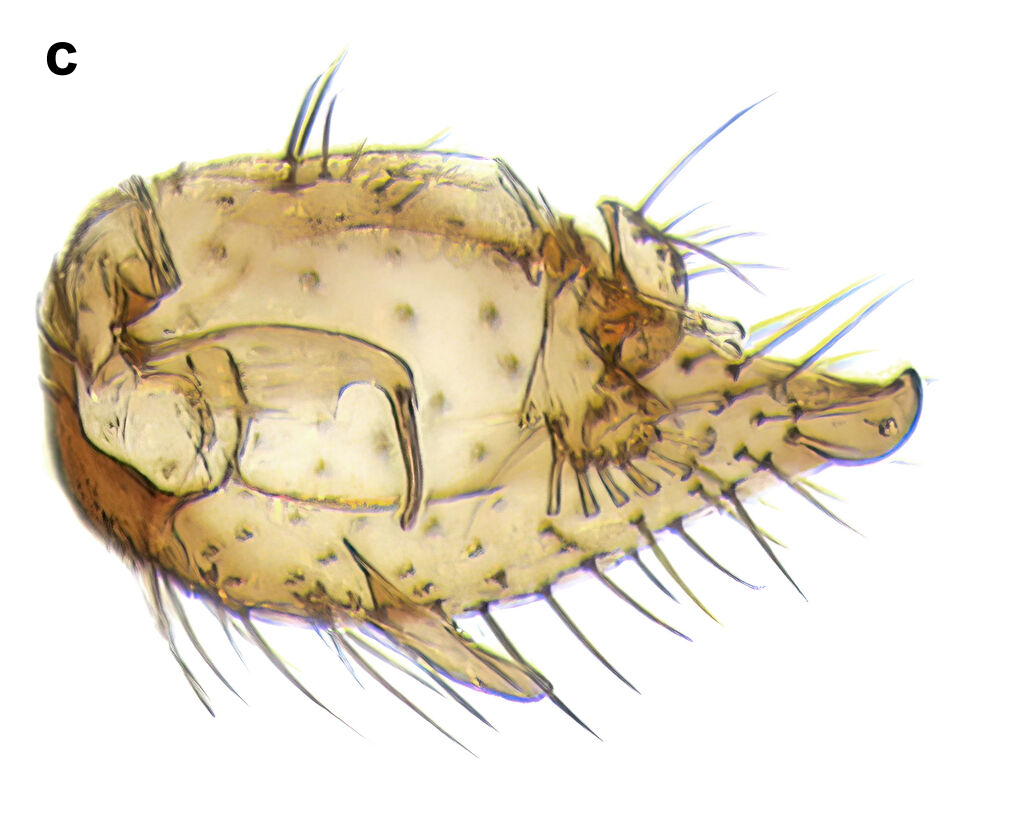
Internal, lateral view

**Figure 12d.**
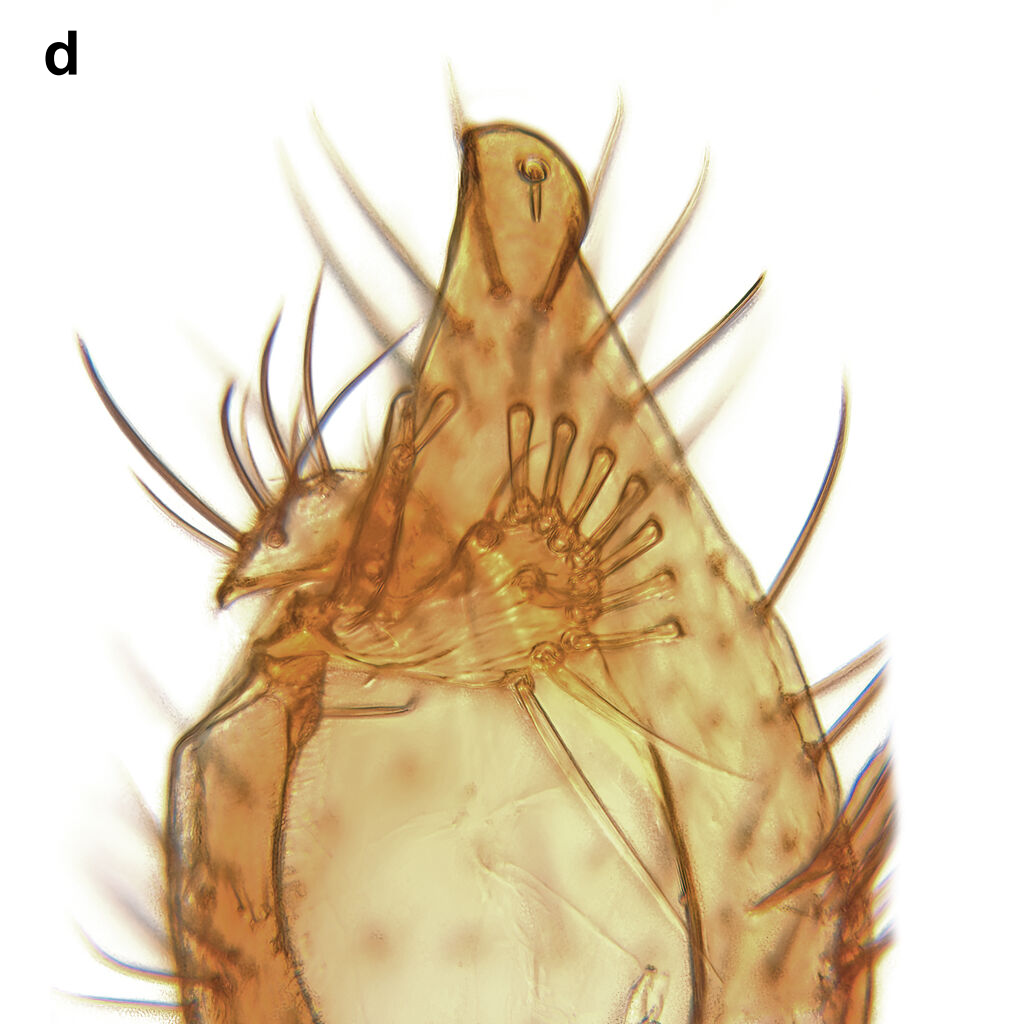


**Figure 12e.**
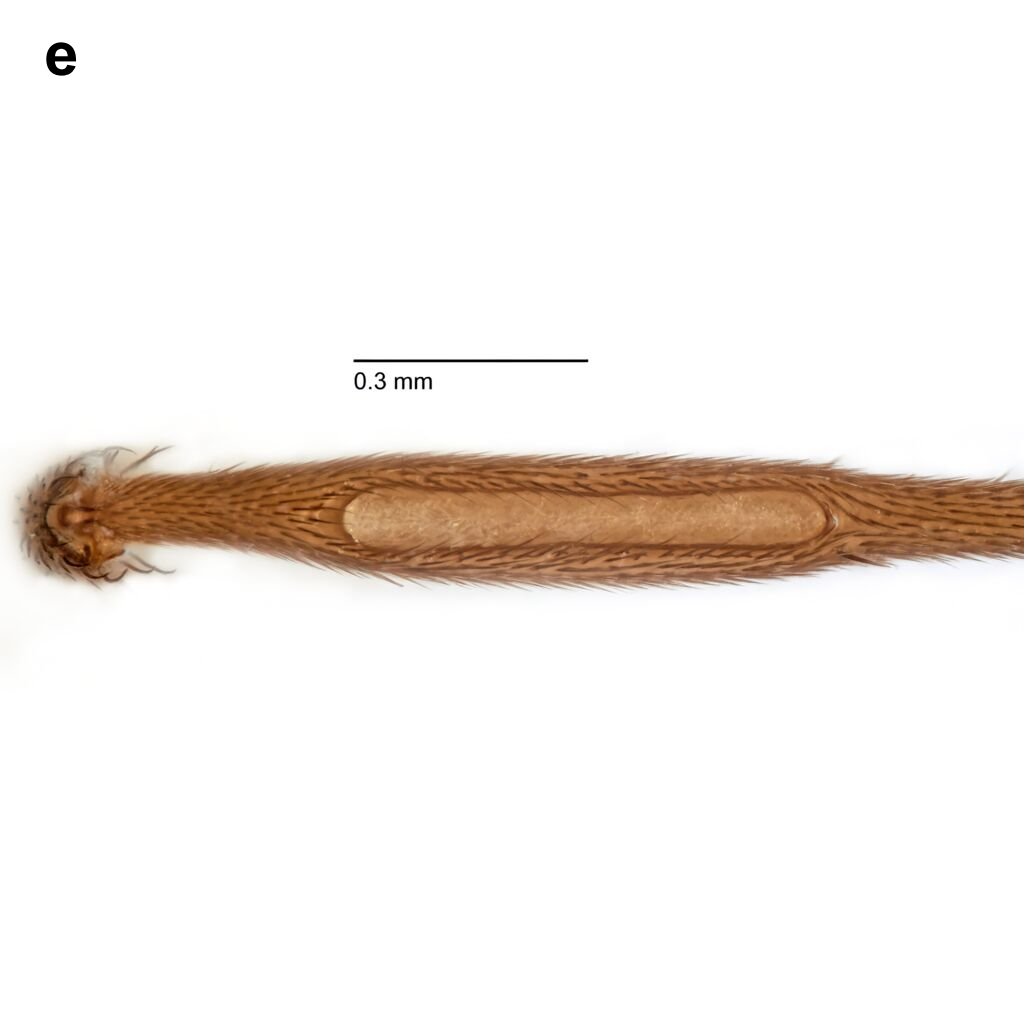
Mid-tibial organ.

**Figure 12f.**
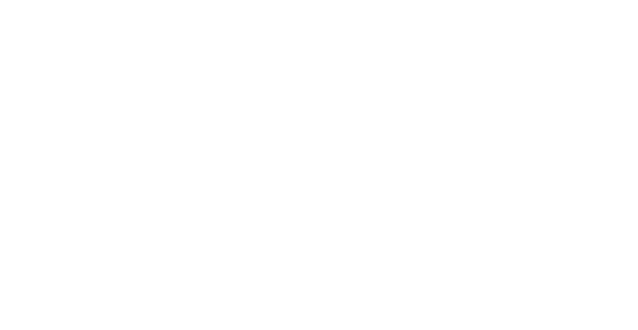


**Figure 13a.**
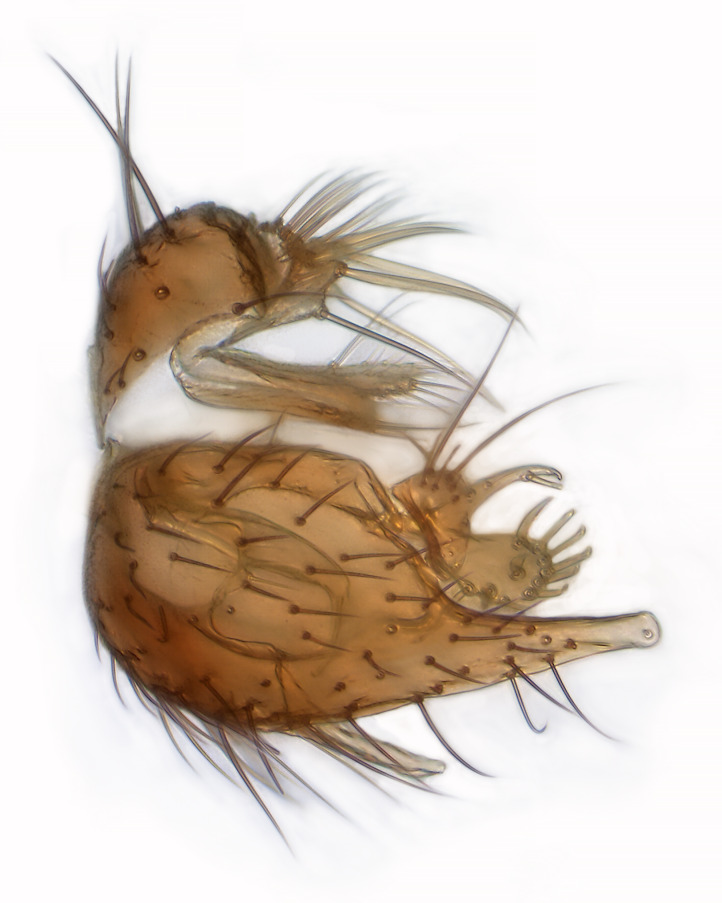
Lateral view

**Figure 13b.**
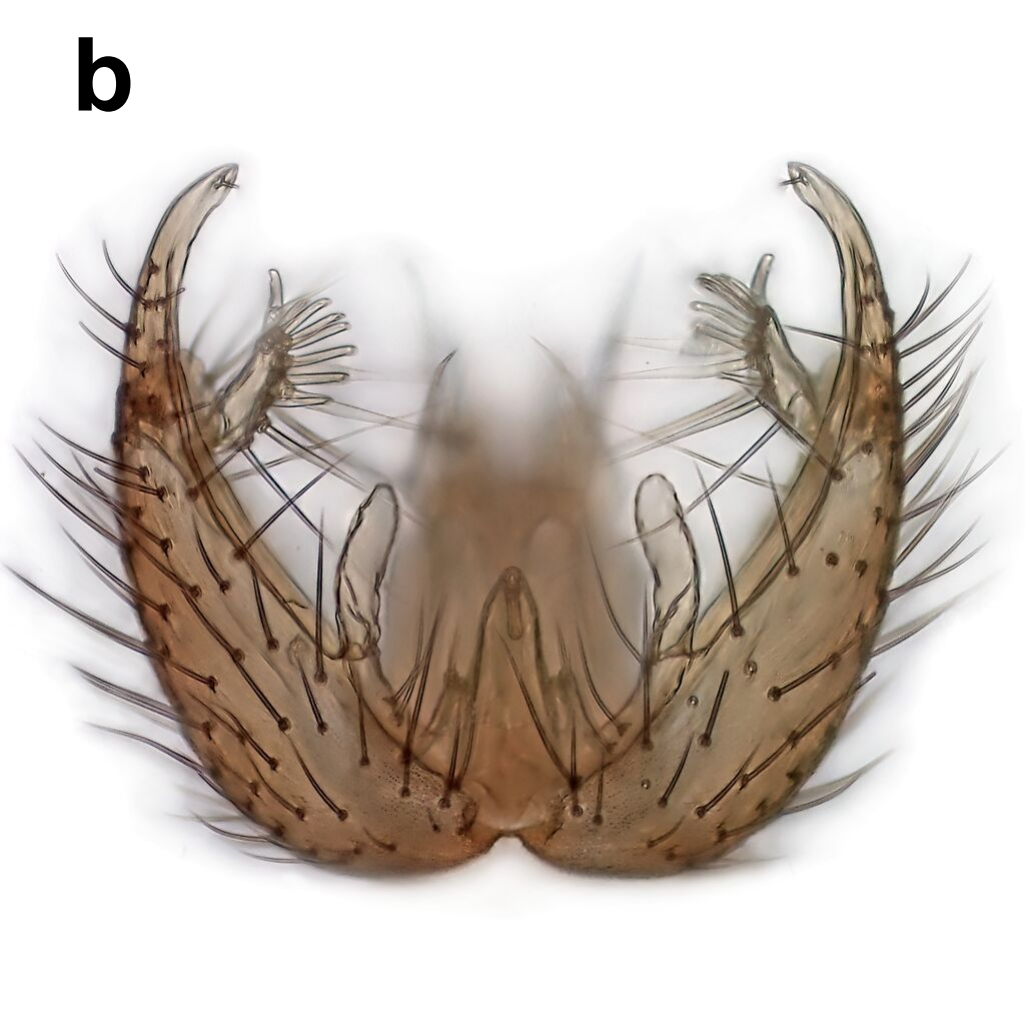
Ventral view

**Figure 13c.**
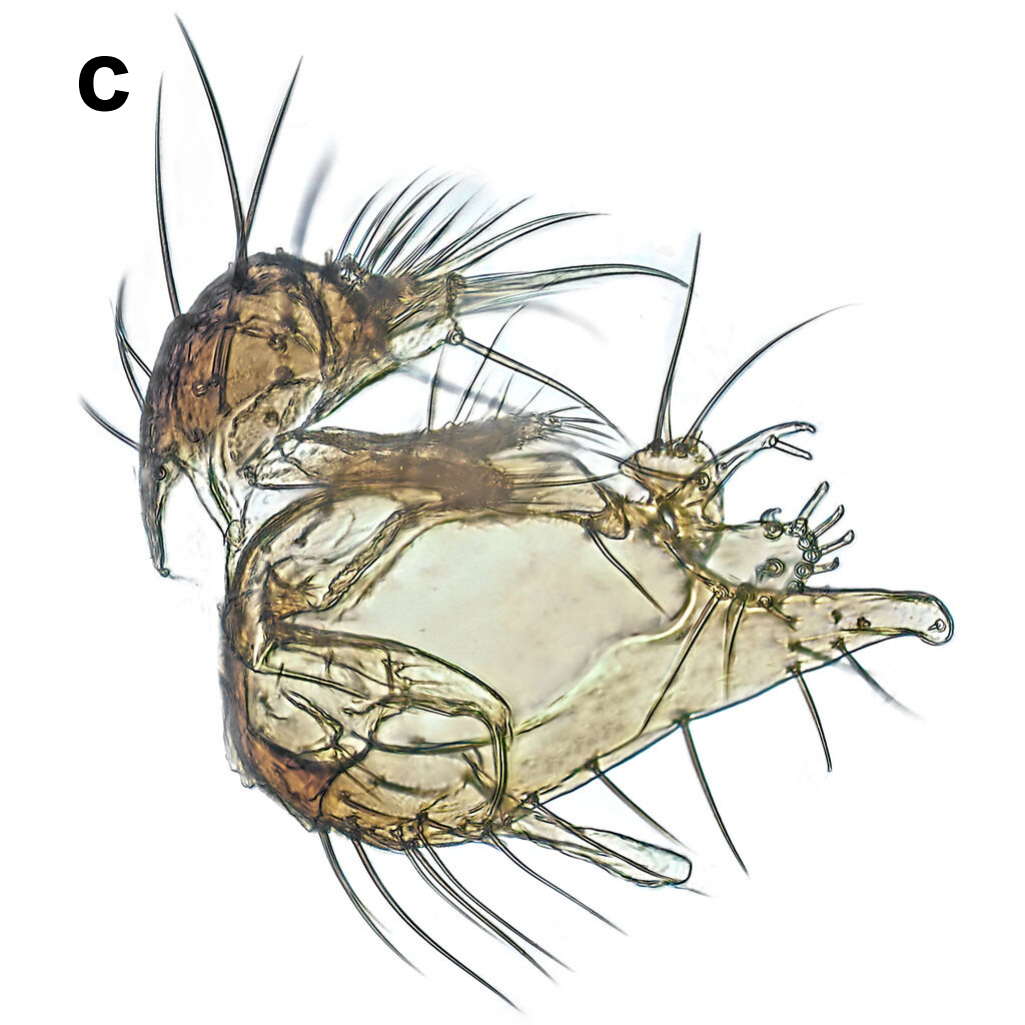
Internal, lateral view

**Figure 13d.**
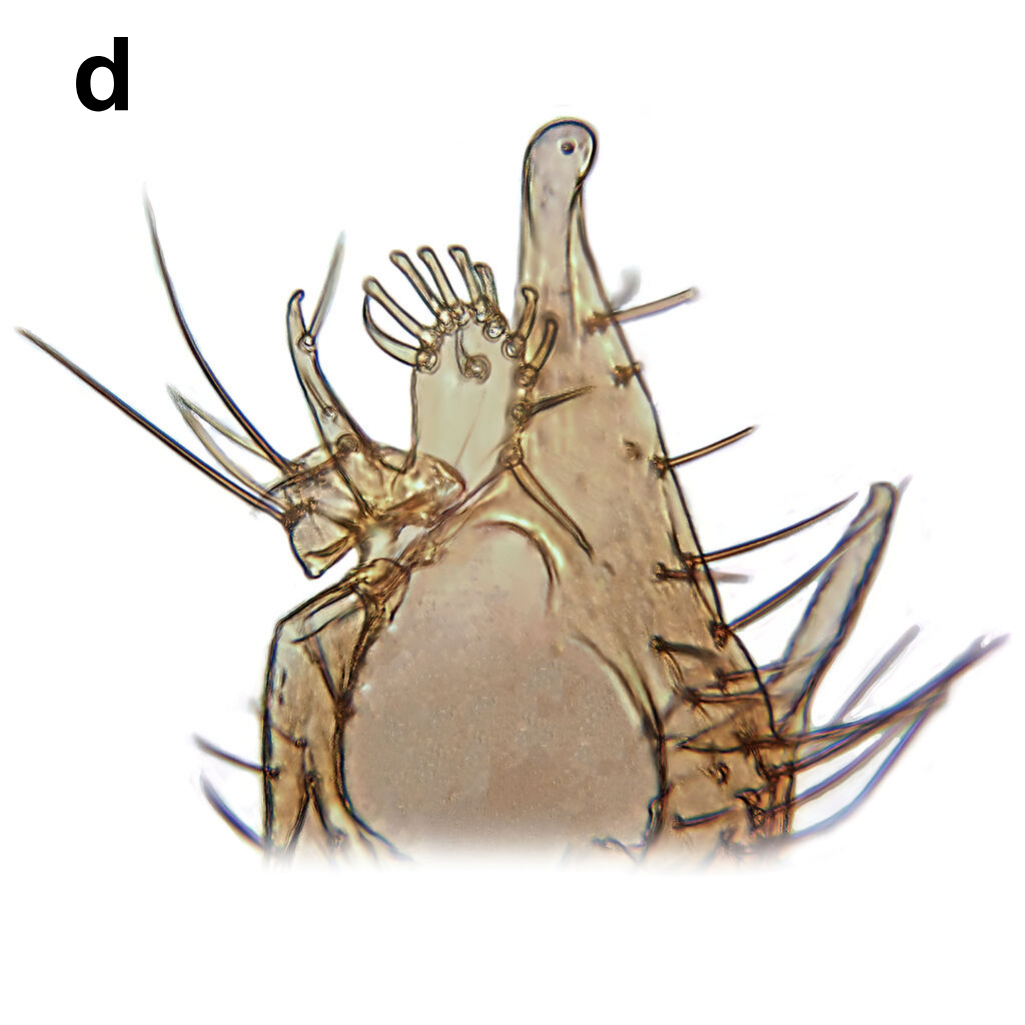
Gonostylus, enlarged, internal view

**Figure 13e.**
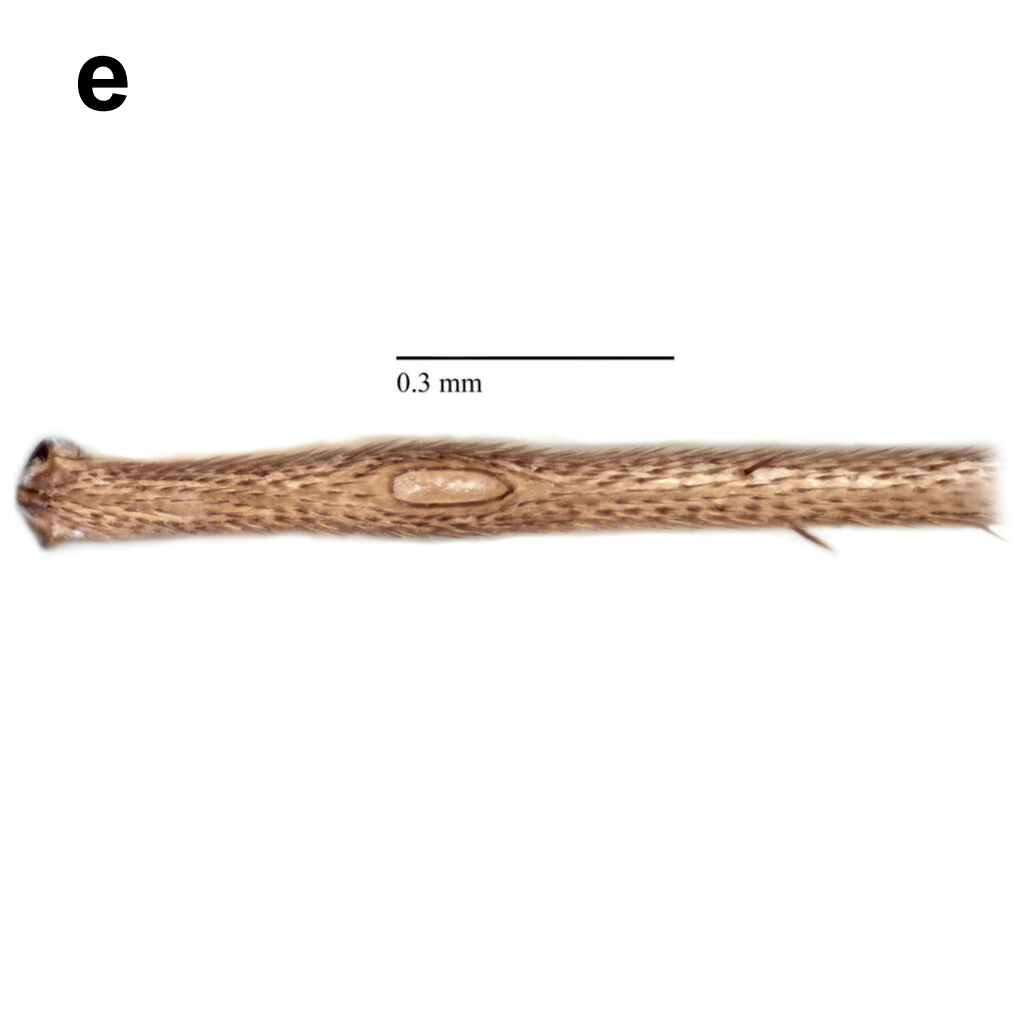
Mid-tibial organ, dorsal view.

**Figure 14a.**
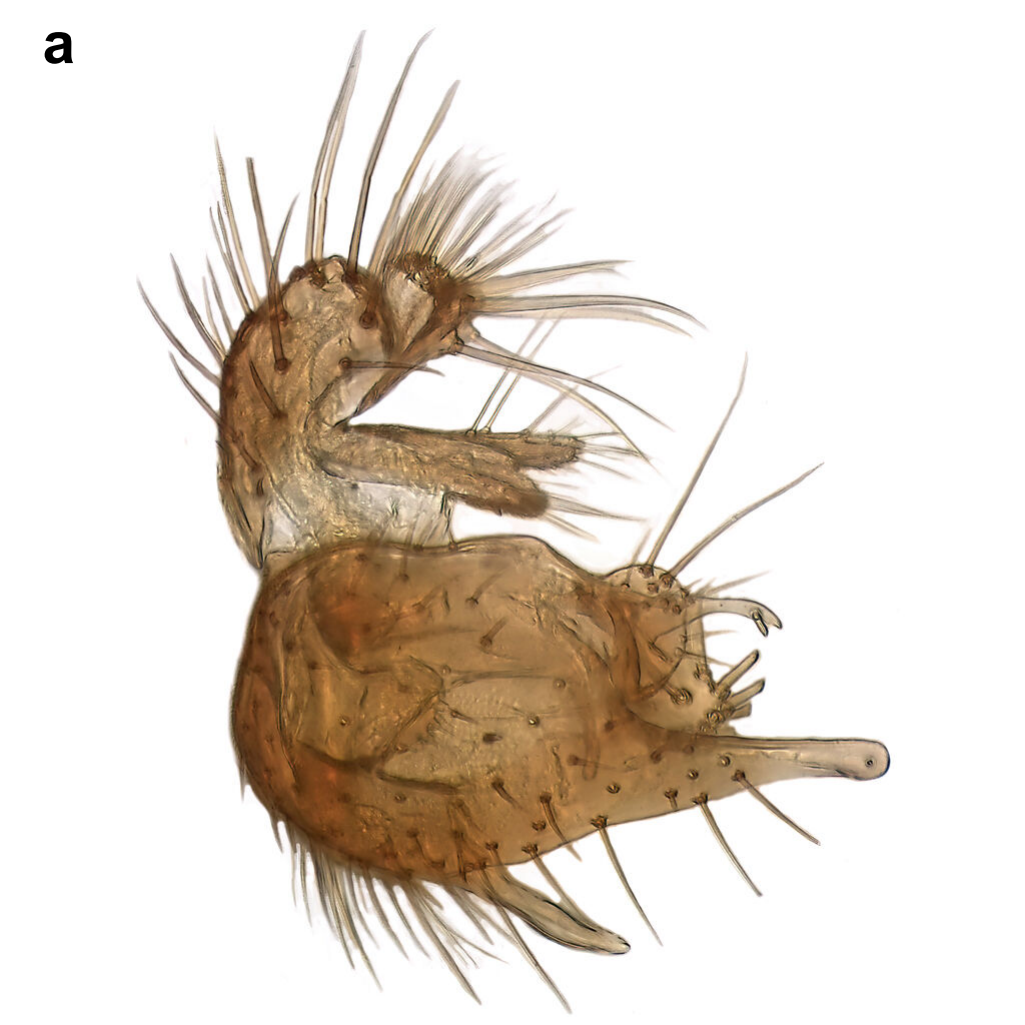
Lateral view

**Figure 14b.**
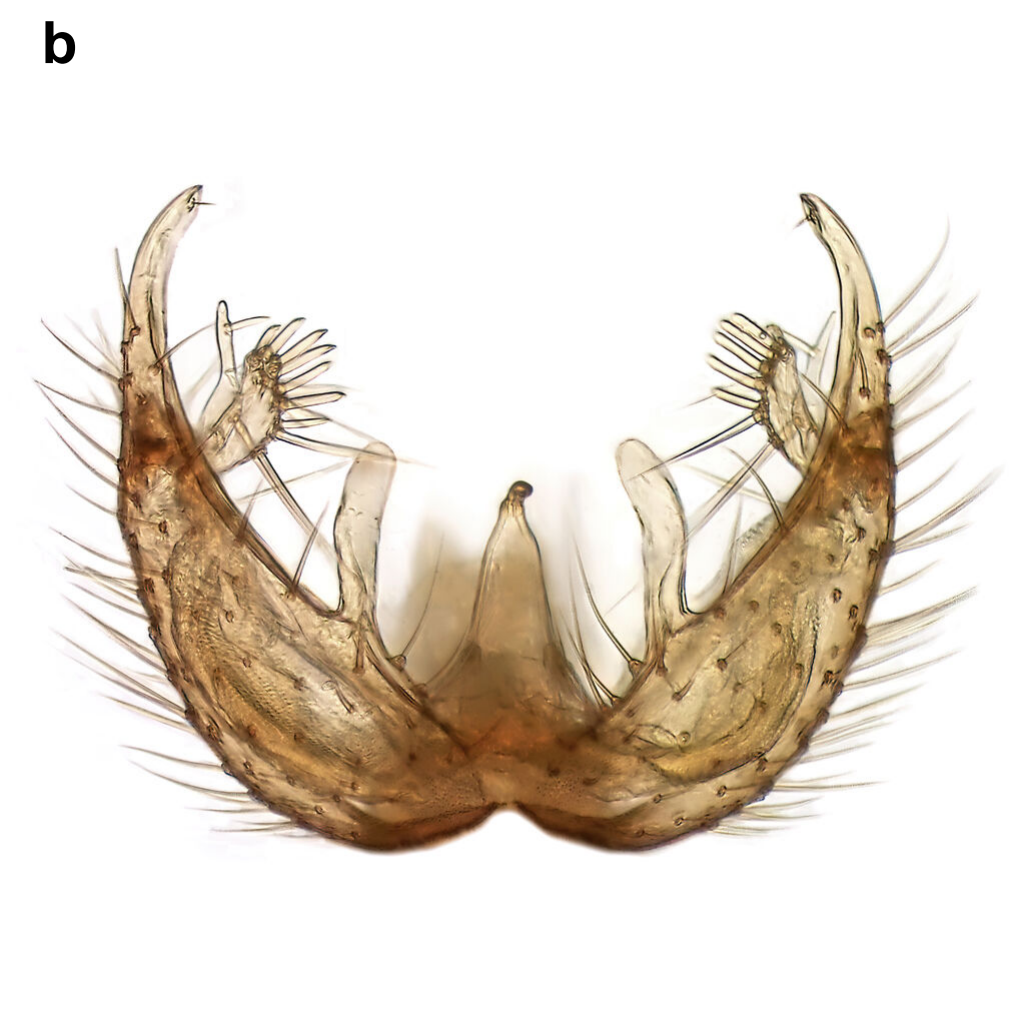
Ventral view

**Figure 14c.**
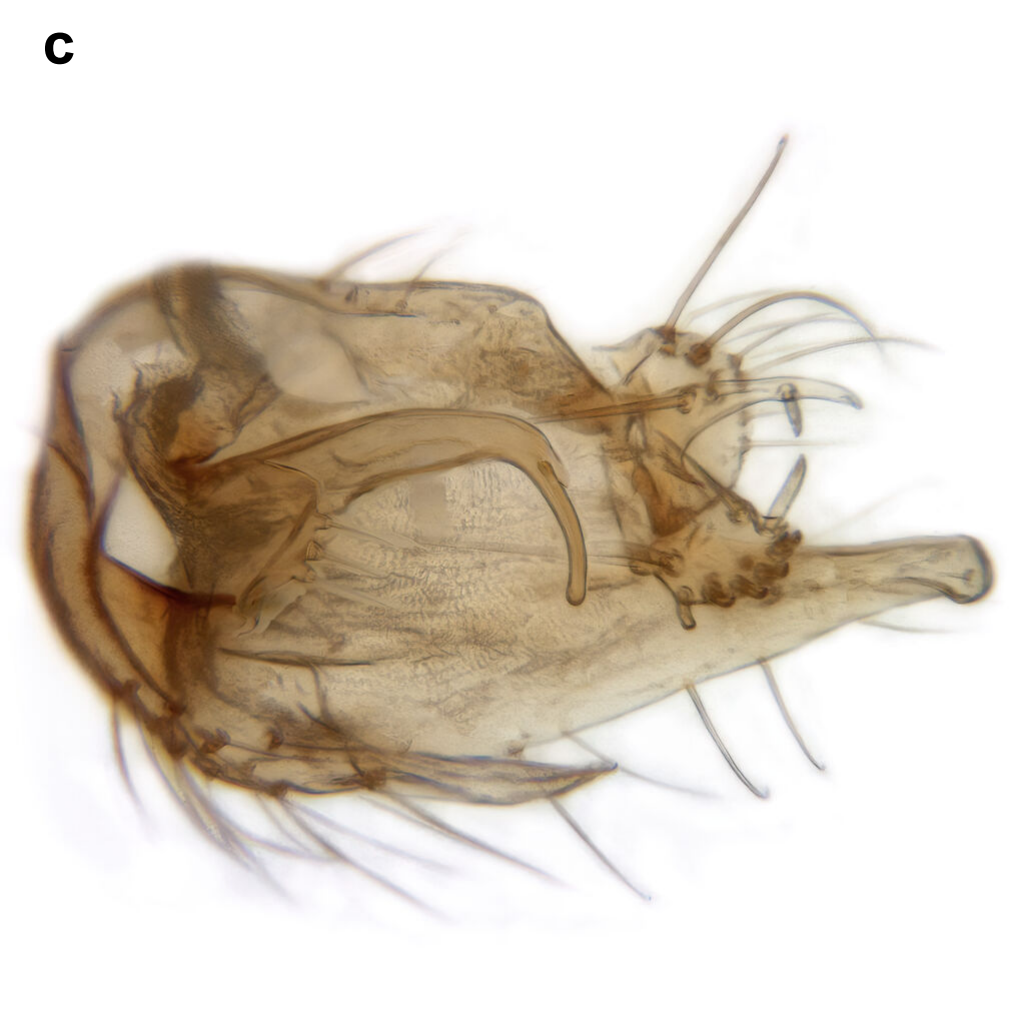
Internal, lateral view

**Figure 14d.**
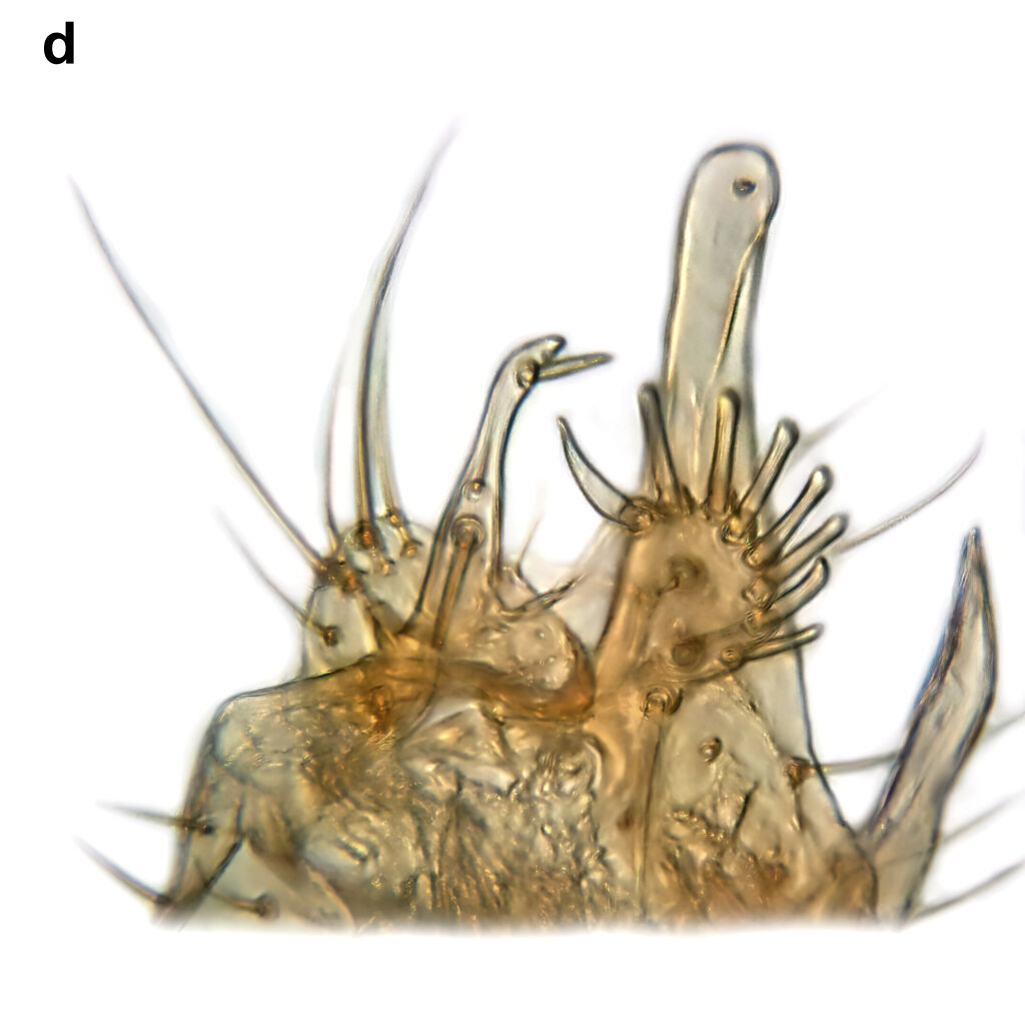
Gonostylus, enlarged, internal view.
