# Supplementary material for: When details matter: Integrative revision of Holarctic Coelophthinia Edwards (Diptera, Mycetophilidae), including mapping of its mitogenome, leads to the description of four new pseudocryptic species
Source: Biodivers Data J. 2023 Feb 14;11:e98741. doi: 10.3897/BDJ.11.e98741 (PMC10848816; doi:10.3897/BDJ.11.e98741)
Supplement: Supplementary material 1 — Mitochondrial gene arrangement [file bdj-11-e98741-s001.pdf]

gene	strand	begin	end	size	start cd	inc
CR I		1	1020	1020		0
trnI	+	1021	1086	66		8
trnQ	-	1095	1163	77		20
trnM	+	1184	1249	86		24
nad2	+	1274	2281	1032	ATC	-2
trnW	+	2280	2347	66		-8
trnC	-	2340	2404	57		18
trnY	-	2423	2487	83		10
cox1	+	2498	4036	1549	ATG	-5
trnL2	+	4032	4097	61		2
cox2	+	4100	4780	683	ATG	5
trnK	+	4786	4856	76		2
trnD	+	4859	4924	68		0
atp8	+	4925	5089	165	ATA	-7
atp6	+	5083	5760	671	ATA	-1
cox3	+	5760	6551	791	ATG	3
trnG	+	6555	6619	68		0
nad3	+	6620	6973	354	ATT	1
trnA	+	6975	7039	66		0
trnR	+	7040	7104	65		1
trnN	+	7106	7171	67		0
trnS1	+	7172	7238	67		1
trnE	+	7240	7308	70		57
trnF	-	7366	7432	124		-17
nad5	-	7416	9140	1708	ATT	24
trnH	-	9165	9228	88		4
nad4	-	9233	10564	1336	ATG	-7
nad4l	-	10558	10854	290	ATA	5
trnT	+	10860	10923	69		0
trnP	-	10924	10988	65		17
nad6	+	11006	11512	524	ATA	18
cob	+	11531	12667	1155	ATG	-2
trnS2	+	12666	12732	65		17
nad1	-	12750	13685	953	ATT	16
trnL1	-	13702	13766	81		-21
rrnL	-	13746	15018	1252		81
trnV	-	15100	15171	153		-2
rrnS	-	15170	15958	787		0
CR II		15959	16199	241		0
