# Supplementary figures and images for: When details matter: Integrative revision of Holarctic Coelophthinia Edwards (Diptera, Mycetophilidae), including mapping of its mitogenome, leads to the description of four new pseudocryptic species
Source: Biodivers Data J. 2023 Feb 14;11:e98741. doi: 10.3897/BDJ.11.e98741 (PMC10848816; doi:10.3897/BDJ.11.e98741)

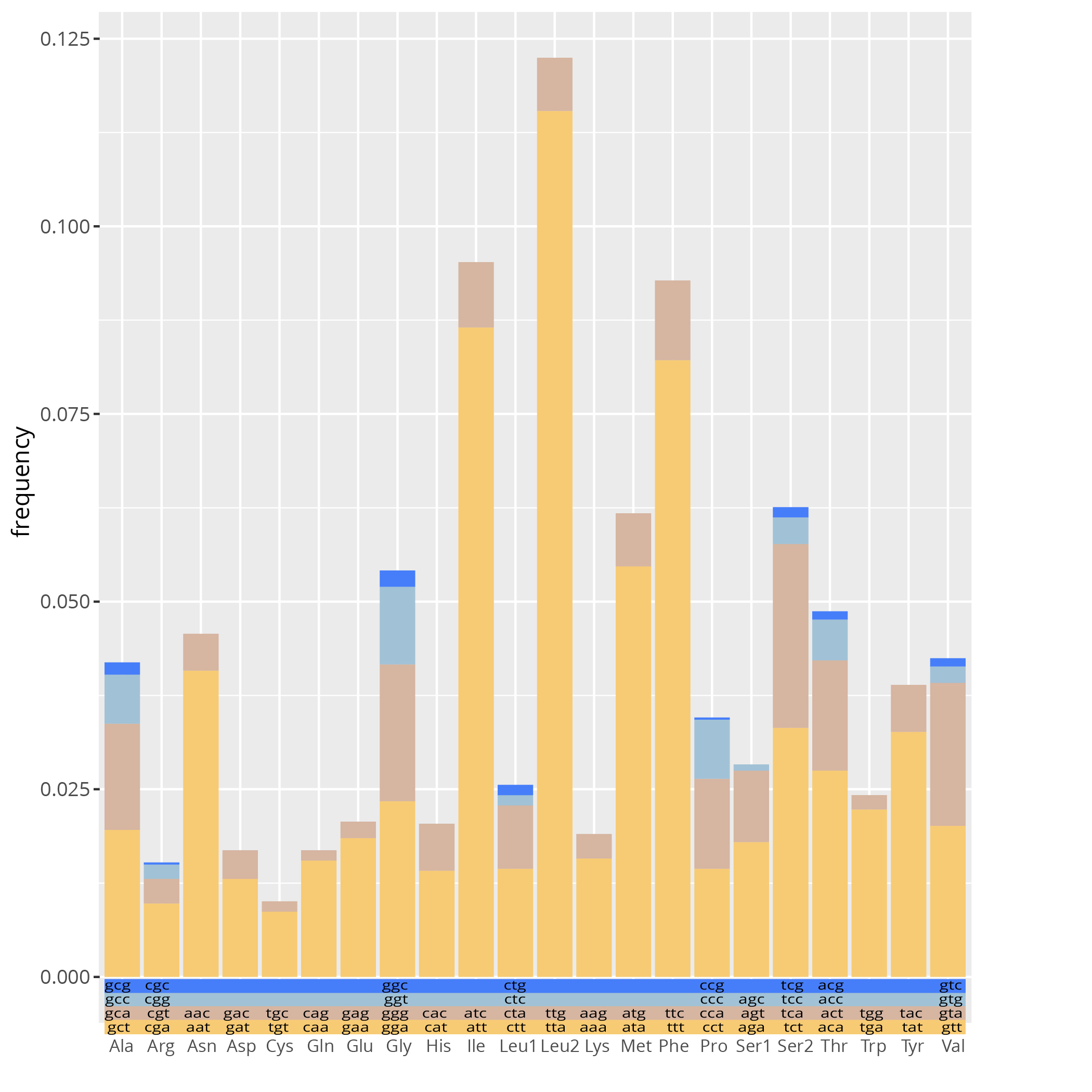

Supplement: Supplementary material 2 — Codon usage barplot [file bdj-11-e98741-s002.jpg]
